# A Diverse Array of Large Capsules Transform in Response to Stimuli

**DOI:** 10.1021/jacs.3c02491

**Published:** 2023-05-16

**Authors:** Kai Wu, Tanya K. Ronson, Leonard Goh, Weichao Xue, Andrew W. Heard, Pingru Su, Xiaopeng Li, Mladen Vinković, Jonathan R. Nitschke

**Affiliations:** † Yusuf Hamied Department of Chemistry, University of Cambridge, Cambridge, CB2 1EW, U.K.; ‡ Astex Pharmaceuticals, 436 Cambridge Science Park, Cambridge CB4 0QA, U.K.; § College of Chemistry and Environmental Engineering, Shenzhen University, Shenzhen, Guangdong 518055, China

## Abstract

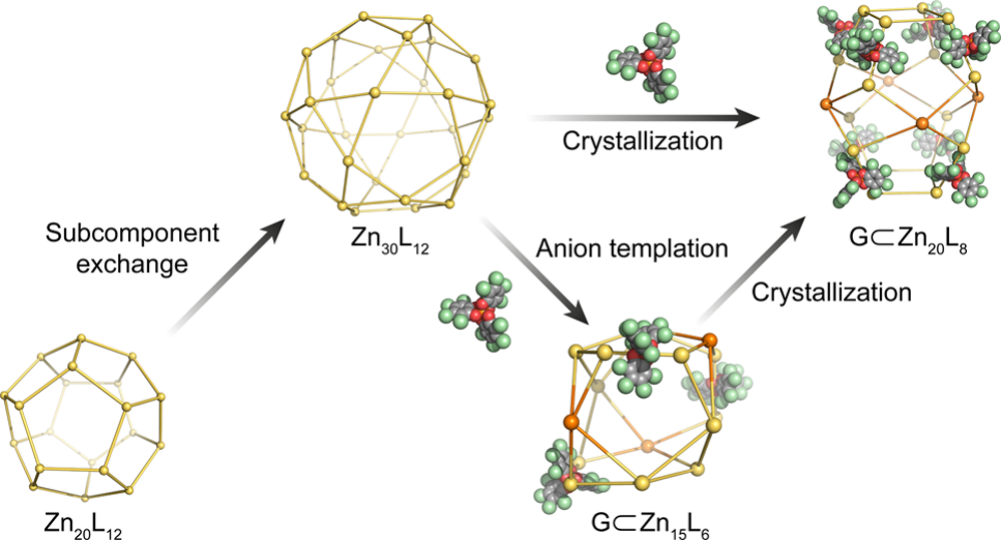

The allosteric regulation of biomolecules, such as enzymes, enables them to adapt and alter their conformation to fit specific substrates, expressing different functionalities in response to stimuli. Different stimuli can also trigger synthetic coordination cages to change their shape, size, and nuclearity by reconfiguring the dynamic metal–ligand bonds that hold them together. Here we demonstrate an abiological system consisting of different organic subcomponents and Zn^II^ metal ions, which can respond to simple stimuli in complex ways. A Zn^II^_20_L_12_ dodecahedron transforms to give a larger Zn^II^_30_L_12_ icosidodecahedron through subcomponent exchange, as an aldehyde that forms bidentate ligands is displaced in favor of one that forms tridentate ligands together with a penta-amine subcomponent. In the presence of a chiral template guest, the same system that produced the icosidodecahedron instead gives a Zn^II^_15_L_6_ truncated rhombohedral architecture through enantioselective self-assembly. Under specific crystallization conditions, a guest induces a further reconfiguration of either the Zn^II^_30_L_12_ or Zn^II^_15_L_6_ cages to yield an unprecedented Zn^II^_20_L_8_ pseudo-truncated octahedral structure. The transformation network of these cages shows how large synthetic hosts can undergo structural adaptation through the application of chemical stimuli, opening pathways to broader applications.

## Introduction

Self-assembled metal–organic cages possessing well-defined cavities find an increasing range of applications, in molecular recognition,^1^ chemical separation,^2^ stabilization of reactive species,^3^ and catalysis,^4^ to give recent examples. In analogy to biological systems,^5^ metal–organic cages can also transform between structures that assemble from a common set of subcomponents. The dynamic nature of such cages, with different cavity sizes and shapes, could lead to different functionalities.^6^

The reversible formation of the coordination bonds that knit cages together enables them to reconfigure in response to an external stimulus.^7^ Different stimuli have been employed to trigger structural transformations, including light,^8^ temperature,^9^ guest templates,^10^ solvents,^11^ redox,^12^ pH,^13^ variation of concentration^14^ and added subcomponents.^15^ The application of one of these stimuli leads to a shift in the thermodynamic balance of the system, triggering bond breaking and reformation in order to reach a new equilibrium. Moreover, structural transformations triggered by different stimuli also provide a means to form cages with increased structural complexity,^16^ which are not accessible through direct coordination-driven self-assembly. Such structural transformations have involved sequences of two or three distinct transformations,^14,15b,17^ leaving room for the development of more complex rearrangements.

## Results and Discussion

**Figure 1:**
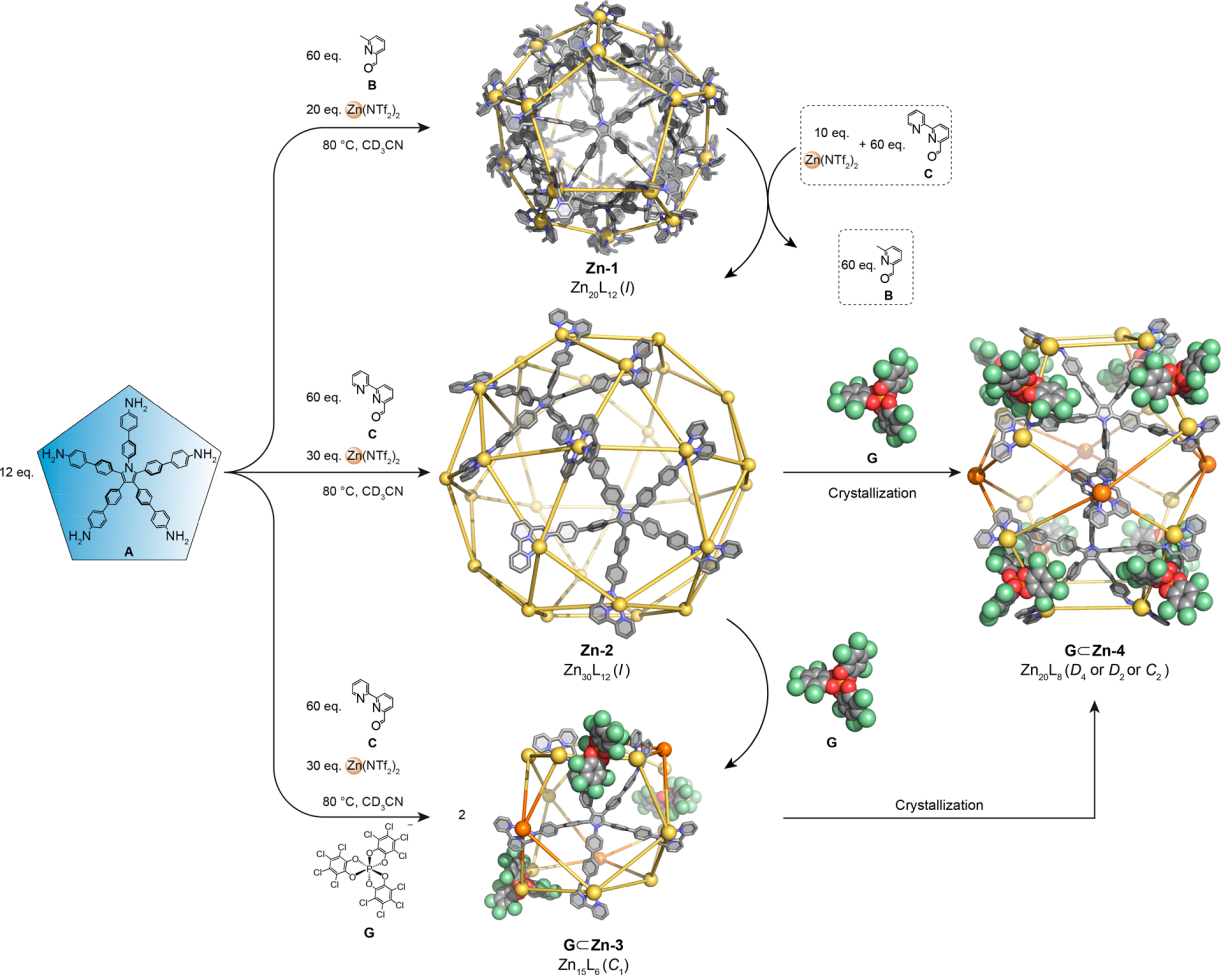
A transformation network showing the effects of chemical stimuli on cages. **Zn-1** is dodecahedral, **Zn-2** is icosidodecahedral, **Zn-3** is a truncated rhombohedron, and **Zn-4** is a previously-unobserved pseudo-truncated octahedron. For **Zn-2**, **Zn-3**, and **Zn-4**, not all ligands are shown for clarity. The point symmetries given in parentheses disregard the rotationally disordered orientations of the pyrrole nitrogen atoms.

Here we demonstrate a network of transformations, shown in Figure 1, between cages that each incorporated a pyrrole-centered penta-amine subcomponent **A**. Different stimuli, such as the addition of subcomponents, metal ions, or a change in temperature, triggered the transformation of one cage into another. The use of graph theory to extend our original series of truncated polyhedra^18^ allowed us to characterize a new pseudo-truncated octahedral structure, which crystallized with either *D*_4_, *D*_2_, or *C*_2_ symmetry. In contrast to the structural diversity we obtained previously,^18^ here a complex transformation network between four large cages and one sandwich structure was realized, with different stimuli triggering structural interconversions.

In this system, Zn^II^_20_L_12_ dodecahedron **Zn-1** transformed into Zn^II^_30_L_12_ icosidodecahedron **Zn-2** following the addition of 6-formyl-2,2′-bipyridine **C** (Figure 1). We infer that this transformation proceeded as a result of the stronger affinity of the tridentate ligand for Zn^II^ over the bidentate ligand.^19^ In the presence of Lacour’s Δ-TRISPHAT anion (**G**),^20^ icosidodecahedron **Zn-2** transformed to Zn^II^_15_L_6_ truncated rhombohedron **Zn-3** in solution, where these chiral anions appear to fit well at the edges of the polyhedron, templating its formation. Finally, both Zn^II^_30_L_12_**Zn-2** and Zn^II^_15_L_6_**Zn-3** transformed into the truncated octahedral architecture of Zn^II^_20_L_8_**Zn-4** during crystallization in the presence of **G**.

**Figure 2:**
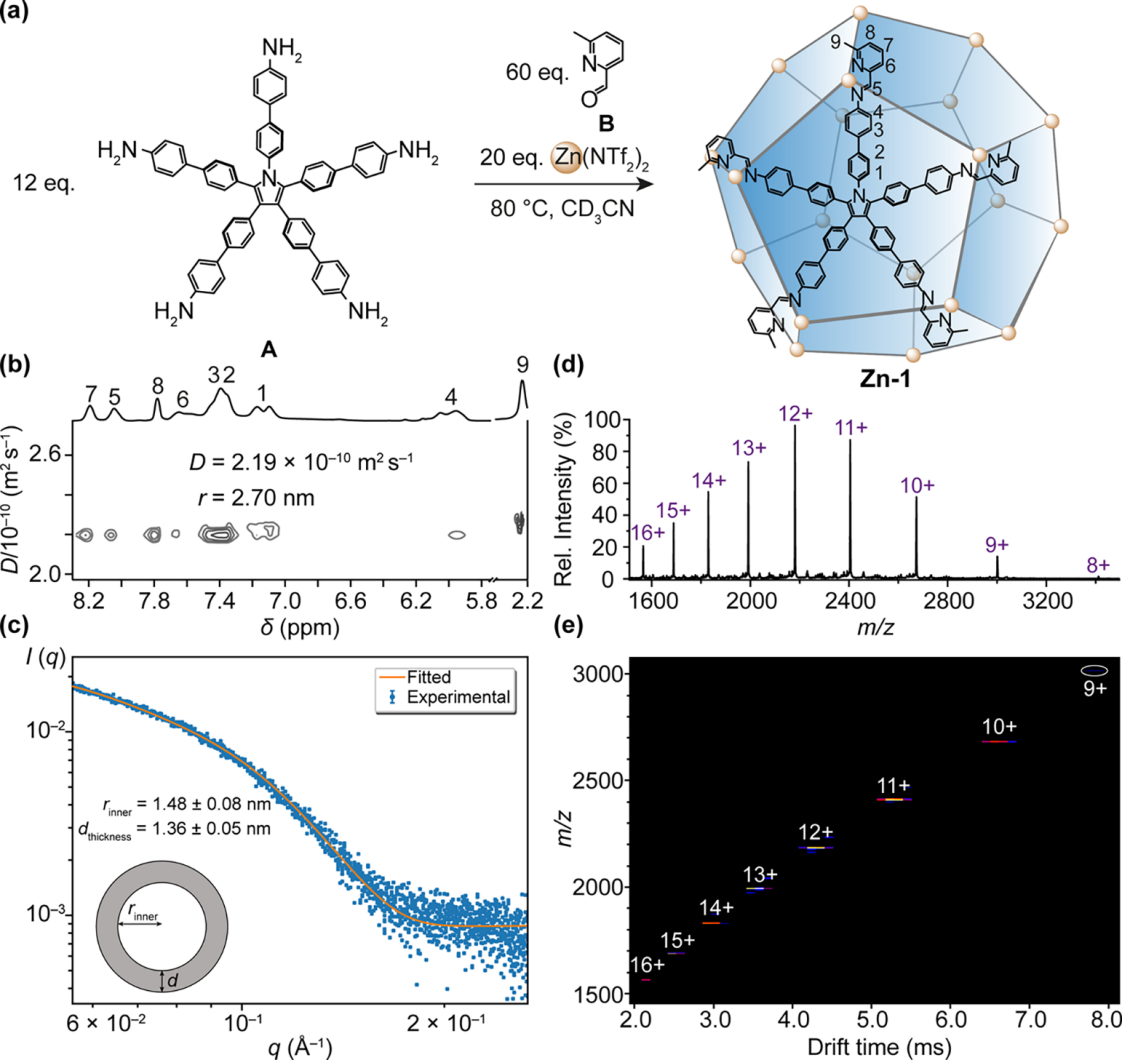
Schematic representation and characterization of dodecahedral cage **Zn-1.** (a) Subcomponent self-assembly of dodecahedral cage **Zn-1**. (b) ^1^H DOSY spectrum (400 MHz, CD_3_CN, 298 K) of **Zn-1**. (c) SAXS profile of **Zn-1** (blue circles) and the fitted result (orange line) based on a core–shell sphere model. The error bars represent uncertainties in the scattering intensity; a larger view is shown in Figure S43. (d) ESI-MS of **Zn-1**. (e) 2D ESI-TWIM-MS plot of **Zn-1** (*m*/*z* vs drift time), both consistent with the presence of a single product.

We hypothesized that penta-amine **A** would condense with methylformylpyridine **B** to generate a pentakis(bidentate) ligand, which would bind to six-coordinate zinc(II) to form M_5*n*_L_3*n*_ structures such as Zn^II^_20_L_12_ dodecahedron **Zn-1**. The reaction of zinc(II) bis(trifluoromethanesulfonyl)imide (Zn(NTf_2_)_2_, 20 equiv) with penta-amine **A** (12 equiv) and **B** (60 equiv) yielded Zn^II^_20_L_12_ cage **Zn-1** in CD_3_CN, as confirmed by NMR spectra, including two-dimensional (2D) NMR techniques (Figures S7–S10). A ^1^H diffusion-ordered spectroscopy (DOSY) experiment indicated the formation of a single species, with a diffusion coefficient (*D*) of 2.19 × 10^–10^ m^2^ s^–1^, corresponding to a solvodynamic radius of 2.7 nm (Figures 2b, S11 and S12).^21^ The results are in line with the expected size of **Zn-1**, consistent with the crystal structure of a previously described Co^II^ analog.^18^ Electrospray ionization mass spectrometry (ESI-MS) further confirmed its Zn^II^_20_L_12_ composition, while traveling wave ion mobility-mass spectrometry (TWIM-MS) data excluded the presence of other isomers (Figure 2d,e). Small-angle X-ray scattering (SAXS) analysis offers a statistically robust method for the structural characterization of large cages.^22^ The structure of **Zn-1** in CD_3_CN was first confirmed by NMR and ESI-MS and further probed by SAXS analysis, revealing the self-assembly of a hollow core–shell spherical cage with an inner radius (*r*_inner_) of 1.48 nm and a shell thickness (*d*_thickness_) of 1.36 nm (Figure 2c).

A distinct strategy was previously developed to construct a series of progressively larger capsules with formulas of M_5*n*_L_2*n*_, where *n* = 1, 2, 3, or 6, by increasing the dihedral angles between pentagonal faces at vertices.^18^ We reasoned that M_5*n*_L_2*n*_ structures with values of *n* missing from this series might be obtained by modifying the angles between the pentagonal faces of these structures. Based on graph theory^23^ we proposed a structure for the Zn^II^_20_L_8_ cage (*n* = 4, Figure S1), having a measured angle of ∼90°. Although ESI-MS signals corresponding to this Zn^II^_20_L_8_ species were found in certain samples of icosidodecahedron **Zn-2**, we were unable to isolate this cage for structural elucidation (Figure S37).

To optimize the selective formation of the Zn^II^_20_L_8_ cage, an anionic template potentially capable of stabilizing the propeller-like configuration of the pentaphenylene pyrrole core of the structure was sought. Δ-TRISPHAT (**G**), with a complementary configuration, was chosen to stack with and bind to the propeller-like pentapyrrole backbone. The reaction of penta-amine **A** (12 equiv) and flexible formylbipyridine **C** (60 equiv) with Zn(NTf_2_)_2_ (30 equiv) in the presence of **G** (18 equiv) resulted in Zn^II^_15_L_6_**Zn-3** (Figure 1), which was observed together with 10–13 **G** anions in HR-ESI-MS (Figure S23), instead of Zn^II^_30_L_12_**Zn-2**. We infer the structure of **Zn-3** to have the same truncated rhombohedral framework as a reported truncated rhombohedron with Co^II^ centers.^18^ Tetrabutylammonium **G** could not be removed even by washing with diethyl ether three times, highlighting the strong interactions between host and guest, which might lead to the cage transformation as discussed below. This truncated rhombohedral structure possesses eight triangular windows and three edge window pockets, where two pentagonal faces share the same edge. These 11 binding pockets may bind 11 **G** anions, consistent with our ESI-MS results.

The interactions between **Zn-3** and **G** were inferred to drive the transformation from **Zn-2** to **Zn-3** and to stabilize the otherwise disfavored **Zn-3** (Figure 1). We hypothesized that the interactions between enantiopure **G** and **Zn-3** might transfer stereochemical information so as to produce **Zn-3** stereoselectively,^24^ considering that the average dihedral angle φ between pentagonal faces along a common edge of **Zn-3** was ca. 82°, comparable to the dihedral angle of ∼88° between two tetrachlorocatecholate groups of **G** (Figure S24).^25^ Circular dichroism (CD) was employed to study this induction process. The CD spectrum of **Zn-3** displayed clear Cotton effects around 250–450 nm from ligand π–π* transitions, indicating chiral induction of the **Zn-3** framework by **G** (Figure S25). Due to the lack of metal-to-ligand charge transfer (MLCT) transitions, the handedness of Zn^II^ centers could not be assigned in solution state by CD spectroscopy.^26^ The stereochemistry of pairs of Zn^II^ centers that bound **G** in **Zn-3** was inferred to possess Δ handedness according to the crystal structures of **Co-3** and **Zn-4**, with the same type of window pockets that also bound **G** tightly (see below). **G** thus acted as a template not only to stabilize the otherwise disfavored **Zn-3** framework but also to control the enantioselectivity of the self-assembly process.

When Co(NTf_2_)_2_ was used instead of Zn(NTf_2_)_2_ under otherwise identical conditions to those used to produce **Zn-3**, truncated rhombohedral Co^II^_15_L_6_ cage **Co-3** was obtained, as confirmed by HR-ESI-MS (Figure S28). CD spectra (Figure S25) revealed similar Cotton effects to those observed in the case of **Zn-3**, suggesting chiral induction also occurred in this case.

**Figure 3:**
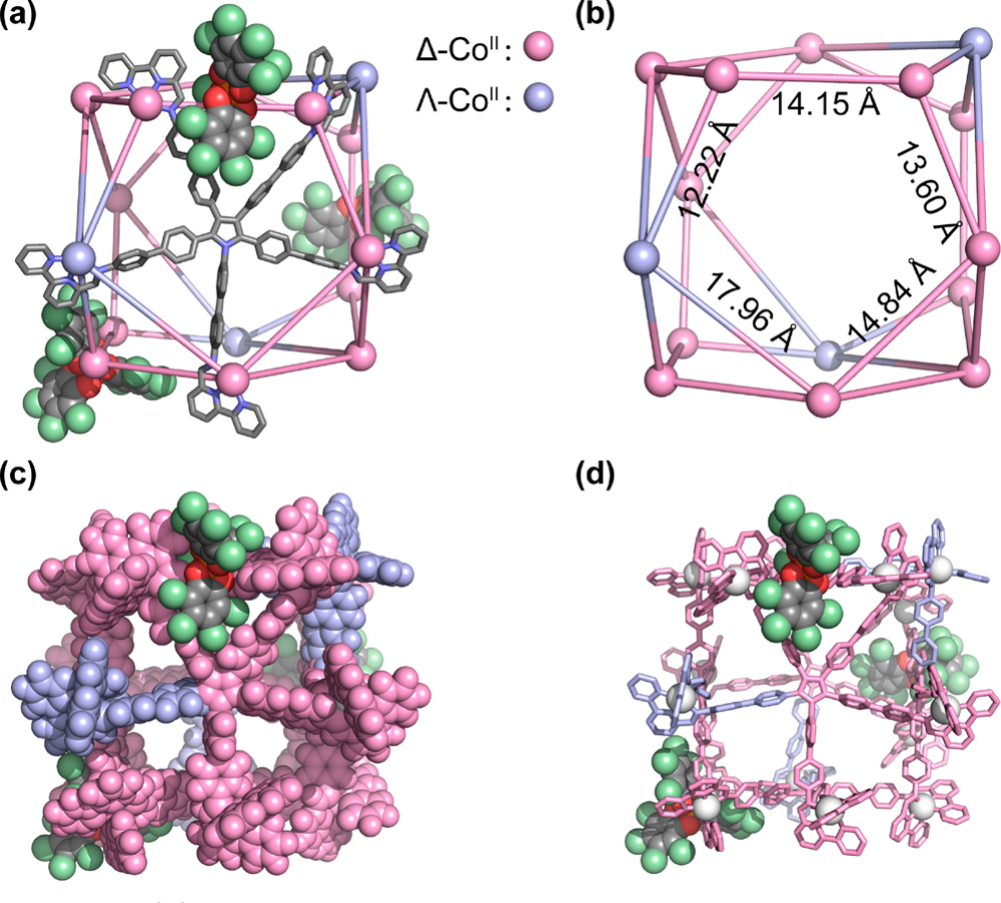
(a) X-ray crystal structure of the truncated rhombohedron **Co-3** and the binding of **G** in window binding pockets; only one ligand is shown for clarity. (b) **Co-3** framework with the different Co^II^···Co^II^ distances shown. (c) Space-filling view highlighting the stacking interactions with **G**, where blue or purple represents ligand environments around the Λ- or Δ-Co^II^ centers, respectively. (d) Ligands shown in ball and stick mode, with all Co^II^ centers colored white.

Although numerous attempts to obtain the crystal structure of **Zn-3** invariably led to its transformation to a new Zn^II^_20_L_8_ cage, **Zn-4** (see below), we were able to grow crystals of **Co-3** suitable for single-crystal X-ray diffraction. X-ray crystallographic analysis confirmed the formation of the truncated rhombohedral cage **Co-3** (Figure 3, M_5*n*_L_2*n*_ series, *n* = 3). **Co-3** crystallized in the chiral *P*3_1_21 space group, with one and a half whole cage units in the asymmetric unit. Three out of 11 **G** anions bound at the pores where pentagonal faces share edges. The driving forces for this binding were inferred to be arene stacking between phenylene rings from different ligand arms and the tetrachlorocatecholate rings of **G**, as well as electrostatic interactions between anionic **G** and cationic Co^II^ centers (Figure 3a). All Co^II^ centers were bound by two tridentate ligand arms, with three out of the 15 Co^II^ centers adopting opposite Λ handedness, resulting in different Co^II^···Co^II^ distances (Figure 3b).

**Figure 4:**
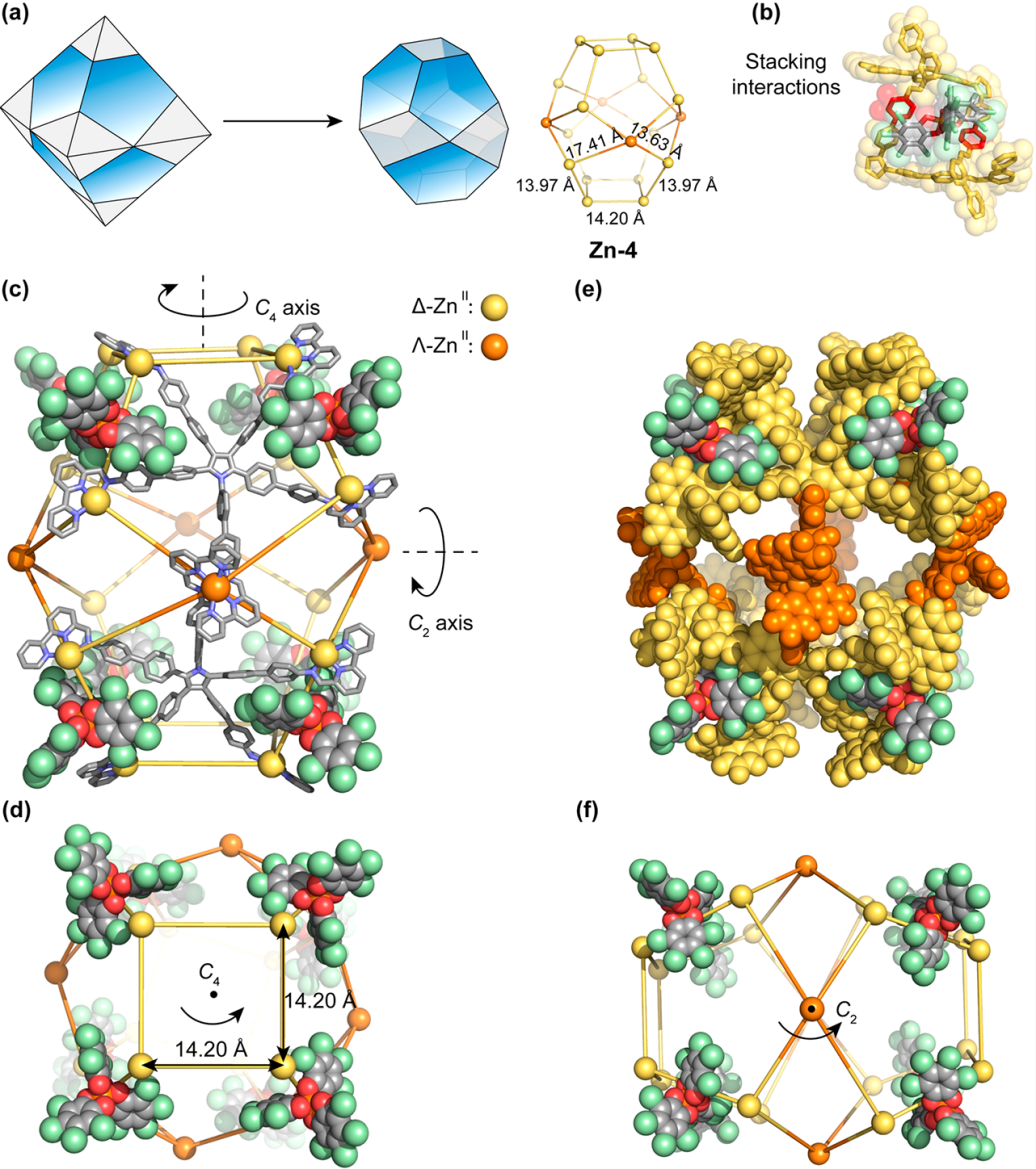
Schematic representation and X-ray crystal structures of **Zn-4**. In all cases hydrogen atoms, nonencapsulated anions, solvents, and disorder are omitted for clarity. (a) Schematic representation of the truncation of an octahedron to produce the framework of **Zn-4**, with the different Zn^II^···Zn^II^ distances shown on the metal framework. (b) Cutaway of the window binding pocket with one bound **G**, highlighting the stacking interactions; phenylene rings from different ligand arms stack with **G** are colored red. (c and d) Side and top views of **Zn-4** that exhibited *D*_4_ symmetry, showing the main *C*_4_ axis, one of four *C*_2_ axes, and Zn^II^···Zn^II^ distances on the square window. The four Λ-Zn^II^ centers of **Zn-4** are colored orange, and the other 16 Δ-Zn^II^ centers are colored yellow. Only two ligands are shown for clarity. (e) Space-filling mode highlighting the stacking interactions with **G**, where orange or yellow shows the ligand environments around the Λ- or Δ-Zn^II^ centers, respectively. (f) Top view of **Zn-4** with one *C*_2_ axis through the Λ-Zn^II^ centers.

Having identified the interactions between **Co-3** and chiral **G**, we then sought to use **G** to influence the stereochemistry of **Zn-2** for structural determination. Vapor diffusion of toluene into a CH_3_CN solution of **Zn-2** in the presence of **G**, however, led to the formation of the new Zn^II^_20_L_8_ cage **Zn-4** (M_5*n*_L_2*n*_ series, *n* = 4) instead of **Zn-2** (Figure 4a). We inferred this transformation to be driven both entropically, by the formation of a smaller assembly, and enthalpically, through the formation of multiple interactions between host and guest, as discussed below. **Zn-4** crystallized in the chiral *I*422 space group with one-eighth of a cage in the asymmetric unit. The four equatorial Zn^II^ centers of **Zn-4** have the same Λ handedness, and the other 16 adopt the opposite Δ handedness (Figure 4c).

Two distinct Zn^II^···Zn^II^ distances between Zn^II^ centers with opposite handedness around the equatorial plane, 13.63 and 17.41 Å, were observed (Figure 4a), where a pentatopic ligand bridges pairs of zinc ions, and the Zn^II^···Zn^II^ distance where two tridentate ligand coordination vectors point away from each other is longer than the case where the vectors point toward each other (Figure S45).^27^ This opposing conformation of one out of the five ligand arms results in opposite handedness of the bound Zn^II^ center, which we inferred to result in a reduction of strain within this irregular structure. Aromatic stacking and electrostatic interactions between **G** and **Zn-4** (Figure 4b) were inferred to induce the structural transformation of **Zn-2** into **Zn-4**. Multiple CH···Cl and CH···O interactions between host and guest, observed in the crystal structure (Figure S46), may also contribute to the stability of **Zn-4**.

The structure of **Zn-4** thus consists of two 4-sided windows in the axial direction, four 4-sided windows at the equatorial plane, and eight pentagonal faces capped by ligands. It has an unprecedented pseudo-truncated octahedral architecture with approximate *D*_4_ symmetry (Figure 4c,d), distinct from a regular truncated octahedron, an Archimedean solid with *O*_*h*_ symmetry.^28^

The replacement of toluene with benzene also yielded single crystals of **Zn-4**, which crystallized in the chiral *I*222 space group, with a quarter of a cage in the asymmetric unit. The distorted pseudo-truncated octahedral framework of this conformation of **Zn-4** has the same connectivity, but approximate *D*_2_ symmetry (Figure S44), with two rhomboid instead of square windows on the top and bottom. Two other distinct truncated octahedral forms of **Zn-4** were also characterized through X-ray crystallography. These crystallized in the *F*222 and *P*4_3_22 space groups and were obtained by using tetrahydrofuran (THF) and diethyl ether as antisolvents, respectively. The four different solid-state structures of **Zn-4** reflect the ability to adapt its configurations in response to the environment, due to its inherent structural flexibility (Figure S44). The different structures of **Zn-4** are discussed in Section 6 of the Supporting Information.

The existence of **Zn-4**, as an isomer of the Zn^II^_20_L_8_ cage predicted by graph theory,^23^ can be understood by extending the M_5*n*_L_2*n*_ structure series with 3-sided windows to 4-sided windows (Figure S2). We further tried to extend the M_5*n*_L_2*n*_ structure series to include architectures with 5-sided windows; however, no integer solution was obtained in this case. We then continued to use a combination of 4-sided and 5-sided faces as windows, i.e., the extension of pseudo-truncated octahedron **Zn-4** by replacing the top and bottom 4-sided windows with 5-sided pentagonal windows. This modification led to an M_25_L_10_ truncated pentagonal bipyramid architecture (Figure S3). We infer that the minor species previously observed in the ESI-MS of icosidodecahedron **Co-2** may correspond to such a structure.^18^

To characterize **Zn-4** in solution, a mass spectrum was measured immediately after dissolving crystalline samples in CH_3_CN (Figure S38). Species corresponding to **G**_8_⊂**Zn-4** in a succession of charge states were found, consistent with the crystal structure, where eight **G** anions bound in the window pockets. Notably, we also found signals corresponding to truncated rhombohedron **Zn-3**, indicating that **Zn-4** transformed into **Zn-3** upon dissolution. We infer that in solution the strong packing effects of the eight **G** holding the pseudo-truncated octahedral architecture together in the solid state will be weakened considerably, leading to partial transformation into the truncated rhombohedron **Zn-3** in solution. Upon dissolution, **Zn-4** thus converted to the more thermodynamically stable product **Zn-3** as the strength of the packing effects mediated by **G** became less pronounced. The metastability of **Zn-4** thus precluded SAXS measurements.

**Figure 5:**
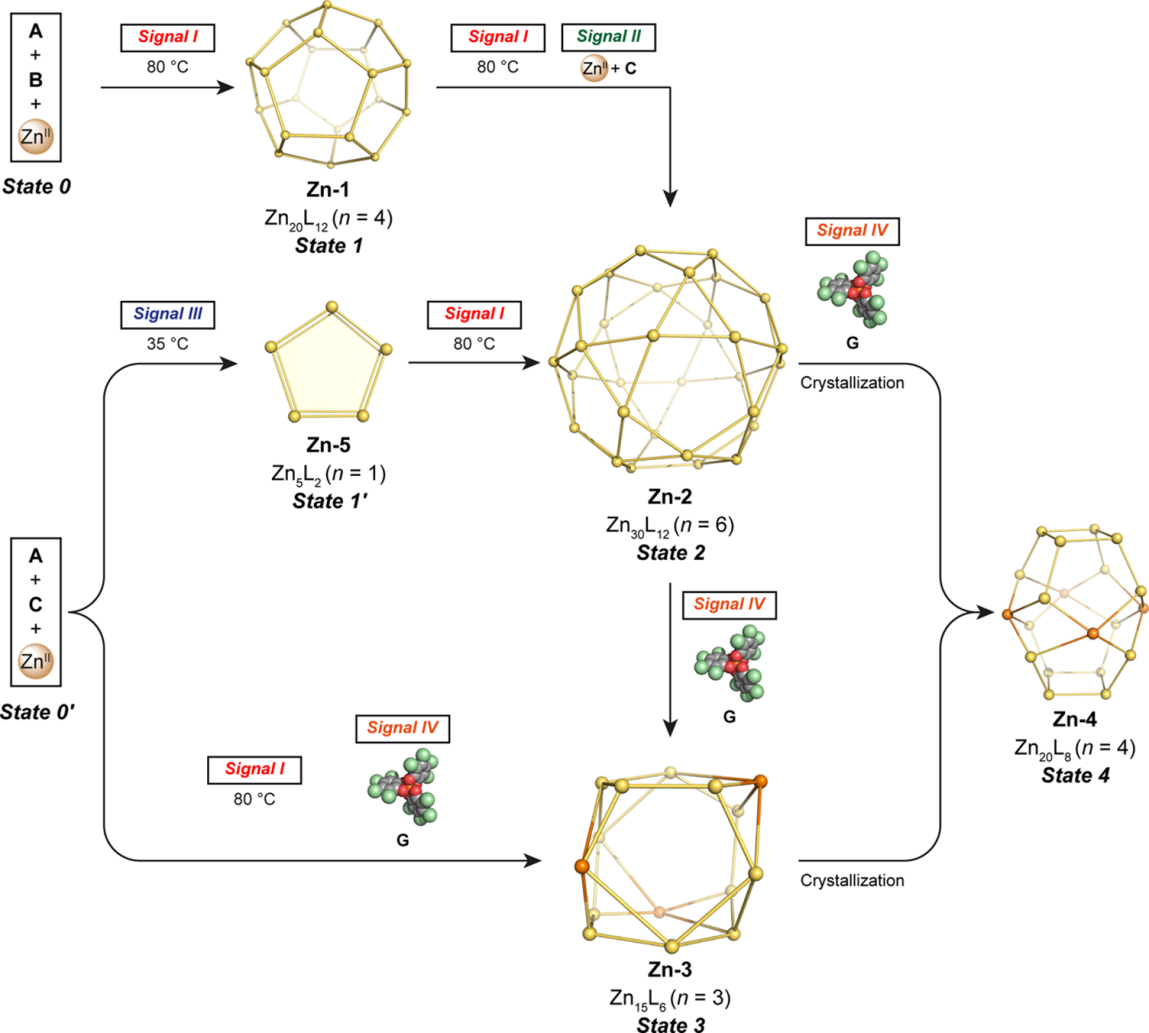
Schematic representation of the transformation network between structures containing penta-amine subcomponent **A**, triggered by different signals. The signals are *I*, 80 °C; *II*, Zn^II^ + **C**; *III*, 35 °C; *IV*, anion **G**.

This reversible chiral-guest-induced structural transformation upon crystallization encouraged us to explore the sequential transformation among the series of M_5*n*_L_2*n*_ and M_5*n*_L_3*n*_ cages, using different stimuli. A hierarchical network of transformations was thus developed, as shown in Figure 5.

The network of Figure 5 charts the ways in which different structures may emerge upon the application of sets of different signals. Starting from penta-amine **A**, aldehyde **B**, and Zn^II^ ions, dodecahedron **Zn-1** with pentakis(bidentate) ligands (*State 1*, Zn^II^_20_L_12_, *n* = 4) formed upon application of *Signal I*, which is heating to 80 °C. Further application of this *Signal I* together with *Signal II*, which is aldehyde **C** and Zn^II^ ions, to **Zn-1** (*State 1*) resulted in formation of product **Zn-2** (*State 2*). Upon the application of *Signal IV*, i.e., anion **G**, **Zn-2** (*State 2*) transformed into truncated rhombohedron **Zn-3** (*State 3*, Zn^II^_15_L_6_, *n* = 3) in solution. Both **Zn-2** (*State 2*) and **Zn-3** (*State 3*) transformed into the pseudo-truncated octahedral topology of **Zn-4** (*State 4*, Zn^II^_20_L_8_, *n* = 4) upon crystallization, with *State 2* requiring *Signal IV* as well to attain *State 4*.

Alternatively, starting from penta-amine **A**, aldehyde **C**, and Zn^II^ ions (*State 0*′), the application of *Signal III*, a temperature of 35 °C, resulted in the formation of Zn^II^_5_L_2_ sandwich structure **Zn-5** (*State 1*′, Figures S29–S36). This metastable *State 1*′ converted into *State 2* upon application of *Signal I*. The application of *Signal I* and *Signal IV* to *State 0*′ provided an alternative path to *State 3*, whereby heating and templation transformed **Zn-5** into **Zn-3**.

Starting from *State 0* or *0*′, five different pathways may be traced through the network to *State 4*, involving the different transitional *States 1–3*. Figure 5 thus represents a kind of logic diagram, showing how different physical inputs are translated into chemical structure outputs. This transformation network could show the way toward the construction of systems that are capable of more complex guest uptake and release through signal-driven structural transformations.

By exploiting the flexible coordination sphere of zinc(II) and the differing coordination preferences of multitopic and multidentate ligands, a transformation network between five complex metal–organic structures, with M_5*n*_L_2*n*_ or M_5*n*_L_3*n*_ formulas, was thus designed. Such transformations rely on the dynamic nature of subcomponent self-assembly, which allows disassembly and reassembly depending on the overall thermodynamic stability of the system. Peripheral guest binding to shape the structure of the metal–organic cages parallels allosteric interactions and control seen in enzymes, where the active site conformation is modified by distant binding. The network reported here in this work sets the stage for future work focusing on tailoring cage cavities for precise control of guest uptake and release.

## Supporting Information Available

Experimental procedures; NMR characterizations; mass spectrometry data; CD spectra; model simulation protocol (PDF)
