# Supplementary material for: A Diverse Array of Large Capsules Transform in Response to Stimuli
Source: J Am Chem Soc. 2023 May 16;145(20):11356–63. doi: 10.1021/jacs.3c02491 (PMC10214450; doi:10.1021/jacs.3c02491)
Supplement: Supplementary file 1 — ja3c02491_si_001.pdf [file ja3c02491_si_001.pdf]

Supporting Information for

A Diverse Array of Large Capsules Transform in Response to Stimuli

Kai Wu,[1] Tanya K. Ronson,[1] Leonard Goh,[1] Weichao Xue,[1] Andrew W. Heard,[1,2] Pingru Su,[3] Xiaopeng Li,[3] Mladen Vinković,[2] and Jonathan R. Nitschke[1]*

[1]Yusuf Hamied Department of Chemistry, University of Cambridge, Lensfield Road, Cambridge, CB2 1EW, UK.

[2]Astex Pharmaceuticals, 436 Cambridge Science Park, Milton Road, Cambridge CB4 0QA, U.K.

[3]College of Chemistry and Environmental Engineering, Shenzhen University, Shenzhen, Guangdong 518055, China

* E-mail: jrn34@cam.ac.uk

Table of Contents

1. General **3**

2. Graph theory analysis of $M_{5n}L_{2n}$ series polyhedral cages **5**

3. Self-assembly of the cages **7**

3.1. Self-assembly of cages with bidentate-armed ligands 7

3.1.1. Self-assembly of Dodecahedron **Zn-1 $[Zn_{20}L_{12}](NTf_2)_{40}$** 7

3.2. Self-assembly of cages with tridentate-armed ligands 15

3.2.1. Templated self-assembly of Truncated Rhombohedron **G⊂Zn-3** 15

3.2.2. Templated self-assembly of Truncated Rhombohedron **G⊂Co-3** 21

3.2.3. Self-assembly of Sandwich structure **Zn-5** 23

4. Sequential transformations of self-assembled cages **29**

4.1. Cage-to-cage transformation from **Zn-1** to **Zn-2** 30

4.2. Cage-to-cage transformation from **Zn-5** to **Zn-2** 33

4.3. Cage-to-cage transformation from **Zn-2** to **Zn-3** 34

5. SAXS measurements **35**

6. X-ray Crystallography **36**

7. Optimized structures **48**

7.1. Cartesian Coordinates of $Zn^{II}_{20}L_8$ isomer 49

8. References **75**

1. General

Materials and methods

Unless otherwise stated, all chemicals were obtained from commercial sources and used as received. Subcomponent **A**, was prepared according to reported procedures.[1]

Nuclear Magnetic Resonance (NMR)

NMR experiments were measured on Bruker AVANCE III and NEO (400, 500 or 600 MHz) spectrometers. Chemical shifts for ^{1}H ^{19}F, ^{31}P, and ^{13}C NMR are reported in ppm with residual solvent as reference: Acetonitrile (1.94 ppm for ^{1}H, 1.32 ppm for ^{13}C), DMSO (2.50 ppm for ^{1}H, 39.52 ppm for ^{13}C). Abbreviations for signal multiplicity of ^{1}H NMR spectra are shown as following: s: singlet, d: doublet, t: triplet, dd: doublet of doublets; dt: doublet of triplets; m: multiplet, br: broad.

Mass spectrometry (MS)

Low-resolution electrospray ionization mass spectrometry (ESI-MS) was undertaken on a Micromass Quattro LC or TheromoFisher TSQ Quantis Plus mass spectrometer. Electrospray Ionization Time-of-Flight (ESI-TOF) and high-resolution electrospray ionization-mass spectrometry (HR-ESI-MS) were performed on a Waters Synapt G2-Si mass spectrometer or Ionspec OFT-7 Fourier-transform ion cyclotron resonance mass spectrometer (Agilent Technologies, Lake Forest, CA) equipped with a 7 T supraconducting magnet and a Micromass Z-spray ESI ion source (Waters, Saint-Quentin, France). The sample flow rate was 4 µL/min, the spray voltage 4.2 kV. All other parameters were optimized for maximum intensities of intact cage ions (sample cone voltage: 50 V; extractor cone voltage: 20 V; desolvation gas: 5 L/min, cone gas: off).

TWIM-MS. TWIM-MS experiments were performed with a Waters Synapt G2-Si mass spectrometer under the following conditions: ESI capillary voltage: 3 kV, sample cone voltage: 30 V, extraction cone voltage: 3.5 V, source temperature: 100 °C, desolvation temperature: 100 °C, cone gas flow: 10 $L{\cdot}h^{-1}$, desolvation gas flow: 700 $L{\cdot}h^{-1}$ (N_2), source gas control: 0 $mL{\cdot}min^{-1}$, trap gas control: 2 $mL{\cdot}min^{-1}$, helium cell gas control: 100 $mL{\cdot}min^{-1}$, ion mobility cell gas control: 30 $mL{\cdot}min^{-1}$, sample flow rate: 5 $\mu L{\cdot}min^{-1}$, ion mobility travelling wave height: 20 – 25 V, ion mobility travelling wave velocity: 350 – 1000 $m{\cdot}s^{-1}$.

Circular dichroism (CD)

UV-vis measurements were employed to fine-tune the solution concentration for subsequent CD measurements, and were performed on a Varian Cary 400 scan UV-vis

spectrophotometer with a 1 mm path-length cuvette at 25 °C. Circular Dichroism was performed on an Applied-Photophysics Chirascan CD spectrometer using a 1 mm path-length cuvette. Experiments were recorded at 298 K, maintained with a Peltier temperature control. Measurements were background subtracted from blank solvent in an identical cuvette. The sample concentrations were adjusted to maintain a HV below 800 V.

Small-angle X-ray scattering. For SAXS measurements, all the SAXS profiles were acquired at the B21 beamline of the Diamond light source in the UK, a dedicated beamline for small angle X-ray scattering. The experiment was performed with the SAXS detector (EigerX 4M, Dectris) positioned 3.7 m from the sample with the beamstop positioned at the top of the detector to give access to the widest possible q (scattering wave vector) range for the data collection. The X-ray energy was 13.0 KeV. The q range was calibrated using silver behenate.

Each supplied stock solution was diluted 2-fold and 10-fold in deuterated acetonitrile to give a range of concentrations for the measurements. Samples were filled into 1.5mm borosilicate capillaries for measurement and sealed using parafilm to prevent evaporation. Capillaries were presented on the beamline using purpose designed 3D printed capillary holders to prevent the delicate borosilicate glass from breaking during sample changeover. The SAXS data were reduced using DAWN, the Diamond developed data analysis workbench application (available for download from https://dawnsci.org/), according to standard protocols. The data were reduced, averaged and background subtracted. In some cases, variation in thickness of the capillaries required a careful approach to background correction where the contribution from the solvent background was varied fractionally to account for the different X-ray path lengths. Borosilicate capillaries are hand blown and some natural variation in thickness can occur which should be accounted for, particularly in weakly scattering systems. To avoid over-subtraction of the background, the background scattering from an empty capillary was subtracted from both the scattering from the solvent and samples measurements, thus removing the contribution from the capillary itself. The solvent scattering was then subtracted from the sample scattering, with a multiplication factor X accounting for the variation in X-ray path length from different diameter capillaries. Some of the low q data from the solvent was rejected during data processing (typically due to flares or shadows) and so this also resulted in truncation of the manually corrected dilute sample data.

Modelling of SAXS data from molecular assemblies has been performed using SASView (https://www.sasview.org/) developed by Diamond in collaboration with other central facilities. The analytical solution was prepared at a concentration of 0.55 mg mL^{-1} for Zn^{II} dodecahedron at 298 K. Acetonitrile was used as blank to conduct background subtraction.

Theoretical calculations

Geometry optimized structures were modelled using the MM3 force field on SCIGRESS software (Fujitsu Limited, Tokyo, Japan, 2013) version FJ 2.6 (EU 3.1.9) Build 5996.8255.20141202.[2]

2. Graph theory analysis of $M_{5n}L_{2n}$ series polyhedral cages

In contrast to the well-known classes of regular convex polyhedra (Platonic, Archimedean, Goldberg polyhedra (smallest dodecahedron **1**), graph theory[3] was used to predict the $V_{5n}F_{2n}$-type cages. Note that the vertices are occupied by metal ions M, and the faces are capped by pentatopic ligands L, therefore $V_{5n}F_{2n} = M_{5n}L_{2n}$.

Key parameters to describe the $V_{5n}F_{2n}$-type polyhedra are shown below. Faces (***F***), Windows (***W_m***), Edges (***E***), Vertices (***V***) and common edges (***l***) represents the number of pentagonal faces, m-sided faces as windows, edges, vertices, and common edges where pentagons share. The composition $\boldsymbol{V = 5n}$, $\boldsymbol{F = 2n}$ would be driven by the principle of maximum site whereby all metal ions are coordinatively saturated, and all ligand nitrogen atoms bound to a metal.

I. $\boldsymbol{V_{5n}F_{2n}}$***-type polyhedra with pentagons and 3-sided triangular windows***

The constraints of Euler's polyhedron theorem and geometrical analysis require:

$$F + W_3 - E + V = 2$$

$$3W_3 + l = E$$

$$5F + 3W_3 = 2E$$

The solution for above equations can be solved:

$$\boldsymbol{V = 5n,\ F = 2n}$$

$$\boldsymbol{W_3 = 4(n-1),\ l = 6-n}$$

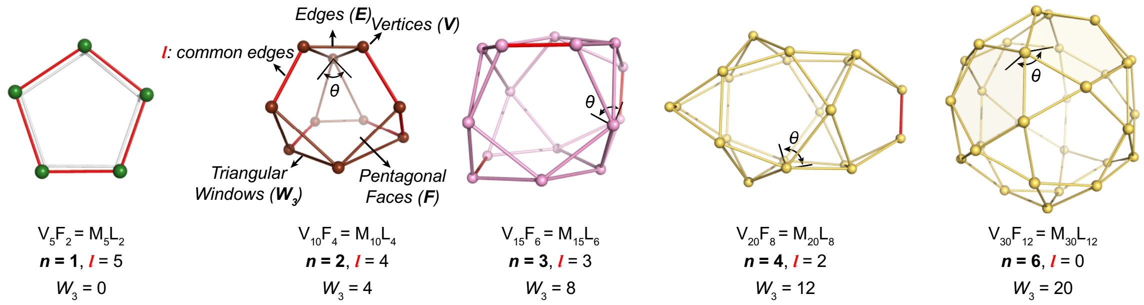

Figure S1. $M_{5n}L_{2n}$ series of cages with pentagonal faces and 3-sided triangular windows. The dihedral angles are 0°, 70.5°, 80.2° and 116.6° for n = 1, 2, 3, 6, respectively. The model of $\mathbf{Zn_{20}L_8}$ (n = 4), has a measured dihedral angle of ~90°.

II. $V_{5n}F_{2n}$ type polyhedra with pentagons and 4-sided square/rhombus windows

We extended the graph theory to 4-sided faces as windows

$$F + W_4 - E + V = 2$$

$$4W_4 + l = E$$

$$5F + 4W_4 = 2E$$

The above equations can be solved:

$$\mathbf{V = 5n,\ F = 2n}$$

$$\mathbf{W_4 = 2(n-1),\ l = n+4}$$

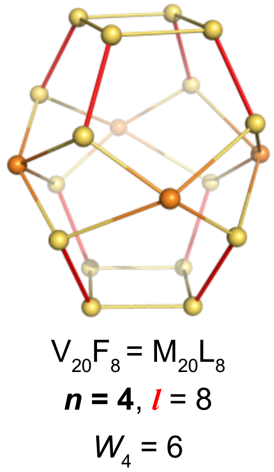

Figure S2. $M_{5n}L_{2n}$ series of cages (**Zn-4**, $\mathbf{Zn_{20}L_8}$, n = 4) with pentagonal faces capped by ligands and 4-sided faces as windows.

III. $V_{5n}F_{2n}$ type polyhedra with pentagons, both 4-sided square/rhombus and 5-sided pentagonal windows

We extended the graph theory to 4-sided and 5-sided faces as windows

$$F + W_4 + W_5 - E + V = 2$$

$$4W_4 + 5W_5 + l = E$$

$$5F + 4W_4 + 5W_5 = 2E$$

As this structure is an extension of **Zn-4** by replacing the top and bottom square windows with pentagonal windows, the above equations can be solved:

$$\boldsymbol{V = 25,\ F = 10}$$

$$\boldsymbol{W_4 = 5,\ W_5 = 2,\ l = 10}$$

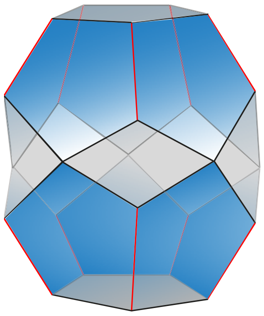

Figure S3. $M_{5n}L_{2n}$ series of cages (**$M_{25}L_{10}$**, *n* = 5) with pentagonal faces, both 4-sided and 5-sided windows (Truncated pentagonal bipyramid).

3. Self-assembly of the cages

3.1. Self-assembly of cages with bidentate-armed ligands

3.1.1. Self-assembly of Dodecahedron **Zn-1 [$Zn_{20}L_{12}$](NTf_2)$_{40}$**

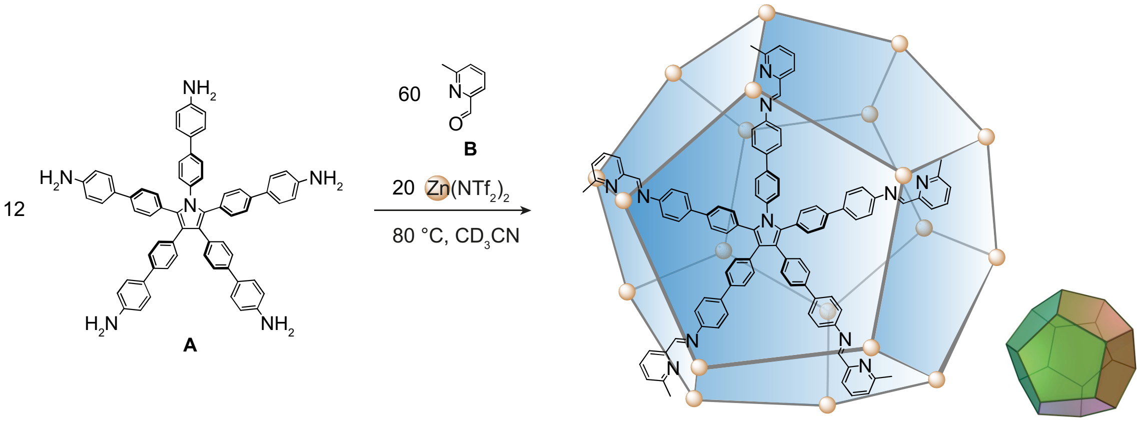

Subcomponent **A** (0.9 mg, 1 µmol), $Zn(NTf_2)_2$ (1.1 mg, 1.7 µmol), 2-formyl-6-methyl pyridine **C** (0.61 mg, 5 µmol) and CD_3CN (0.4 mL) were added to a small test tube, and the mixture was sonicated for 1 min and heated at 80 °C overnight. The brownish-yellow

solution was concentrated by blowing with N_2 and then Et_2O was added. The resulting solid was collected by centrifugation, washed three times with additional Et_2O and then vacuum dried to give dodecahedron **Zn-1 $[Zn_{20}L_{12}](NTf_2)_{40}$** as a brown solid (2.2 mg, 0.075 μmol, yield 90%).

^{1}H NMR (500 MHz, CD_3CN) δ 8.2 (br, 60H), 8.0 (br, 60H), 7.8 (br, 60H), 7.7 (br, 60H), 7.4 (m, 240H), 7.2 (br, 60H), 7.1 (br, 60H), 6.0 (m, J = 48.9 Hz, 120H), 2.2 (br, 180H).

^{13}C NMR (125 MHz, CD_3CN) δ 166.0, 161.8, 147.6, 147.1, 146.8, 142.7, 140.6, 140.1, 139.3, 138.5, 137.4, 136.5, 133.8, 133.1, 132.9, 132.6, 130.2, 128.2, 127.0, 124.3, 122.8, 122.5, 24.8.

^{19}F NMR (376 MHz, CD_3CN) δ –79.99 (s, CF_3).

Low-resolution ESI-MS (CH_3CN): *m/z*: 1062.7 $[M–22NTf_2^-]^{22+}$, 1126.8 $[M–21NTf_2^-]^{21+}$, 1197.1 $[M–20NTf_2^-]^{20+}$, 1274.8 $[M–19NTf_2^-]^{19+}$, 1361.3 $[M–18NTf_2^-]^{18+}$, 1457.9 $[M–17NTf_2^-]^{17+}$, 1566.5 $[M–16NTf_2^-]^{16+}$, 1689.6 $[M–15NTf_2^-]^{15+}$.

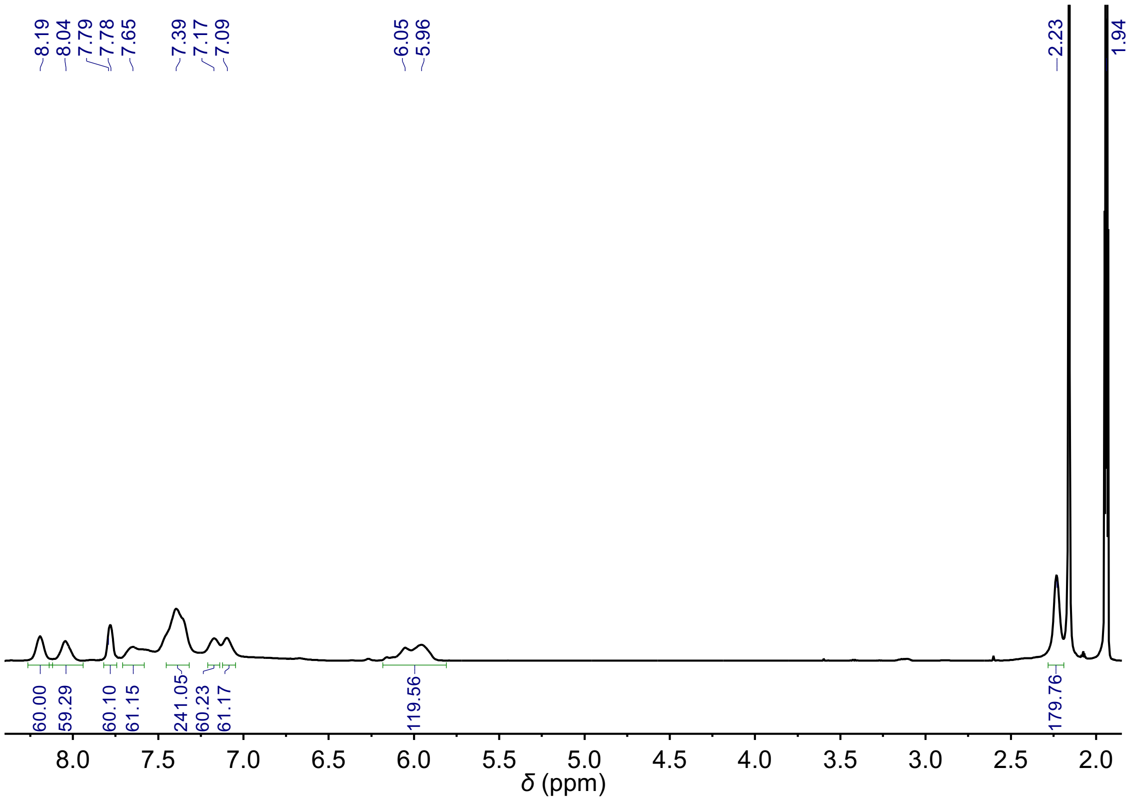

Figure S4. ^{1}H NMR spectrum (500 MHz, 298 K, CD_3CN) of **Zn-1 $[Zn_{20}L_{12}](NTf_2)_{40}$**. Only one set of broad signals of the ligand arms were observed, presumably due to the similarity of the different ligand arm environments as a result of pyrrole-N random orientations, resulting in signal overlap and broadness.

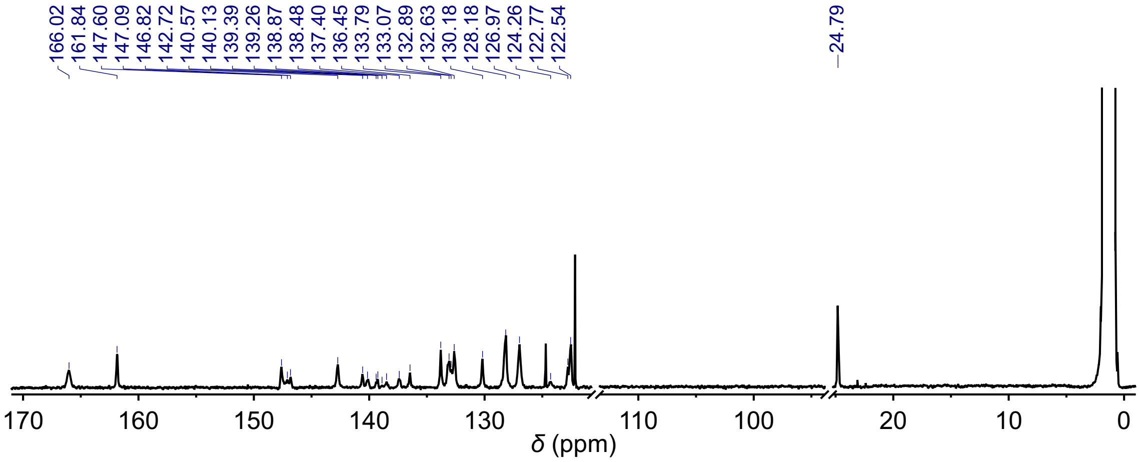

Figure S5. ^{13}C NMR spectrum (125 MHz, 298 K, CD_3CN) of **Zn-1 $[Zn_{20}L_{12}](NTf_2)_{40}$**. ^{13}C peaks are broad presumably due to the many isomers in the system caused by rotationally disordered pyrrole-N and the slow tumbling of the large cage molecules in the solution, thus fewer than the expected number of signals are observed.

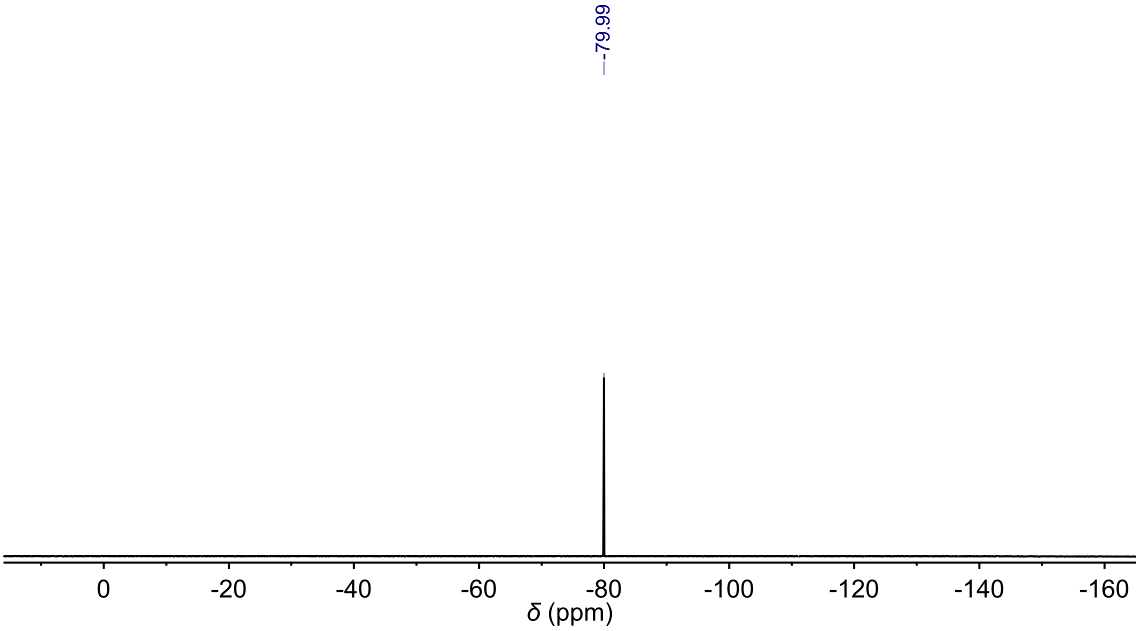

Figure S6. ^{19}F NMR spectrum (376 MHz, 298 K, CD_3CN) of **Zn-1 $[Zn_{20}L_{12}](NTf_2)_{40}$**.

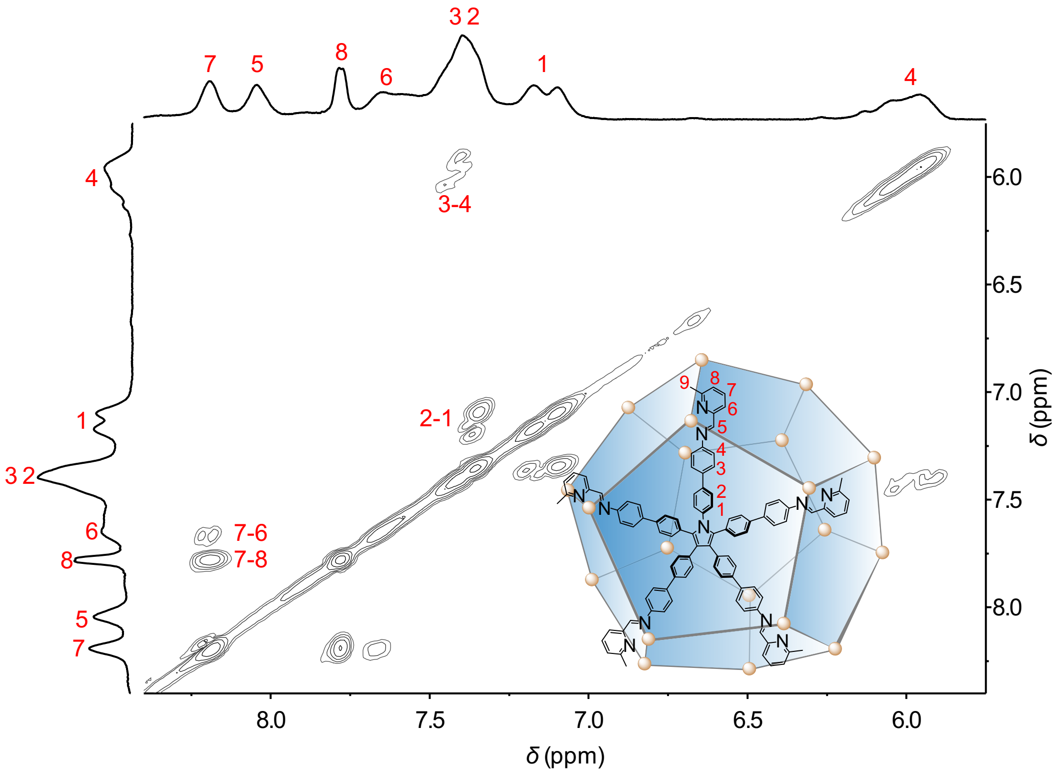

Figure S7. Partial ^1H–^1H COSY spectrum (500 MHz, 298 K, CD_3CN) of dodecahedron **Zn-1 [$Zn_{20}L_{12}$](NTf_2)$_{40}$**.

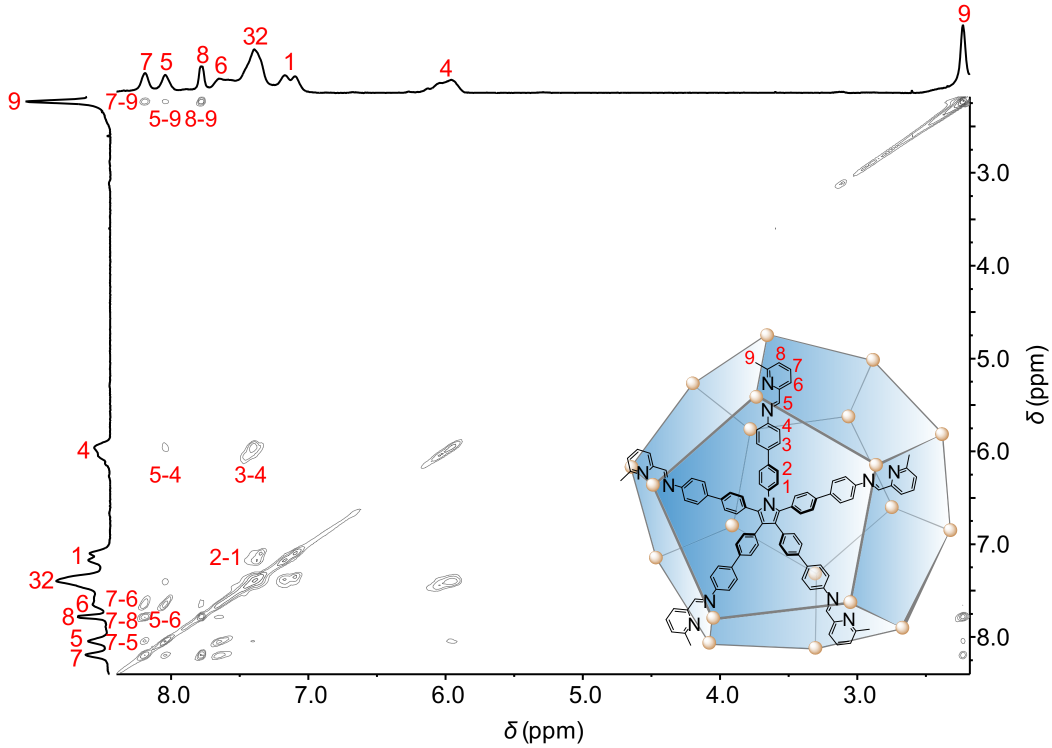

Figure S8. Partial ^1H–^1H NOESY spectrum (500 MHz, 298 K, CD_3CN) of dodecahedron **Zn-1 [$Zn_{20}L_{12}$](NTf_2)$_{40}$**.

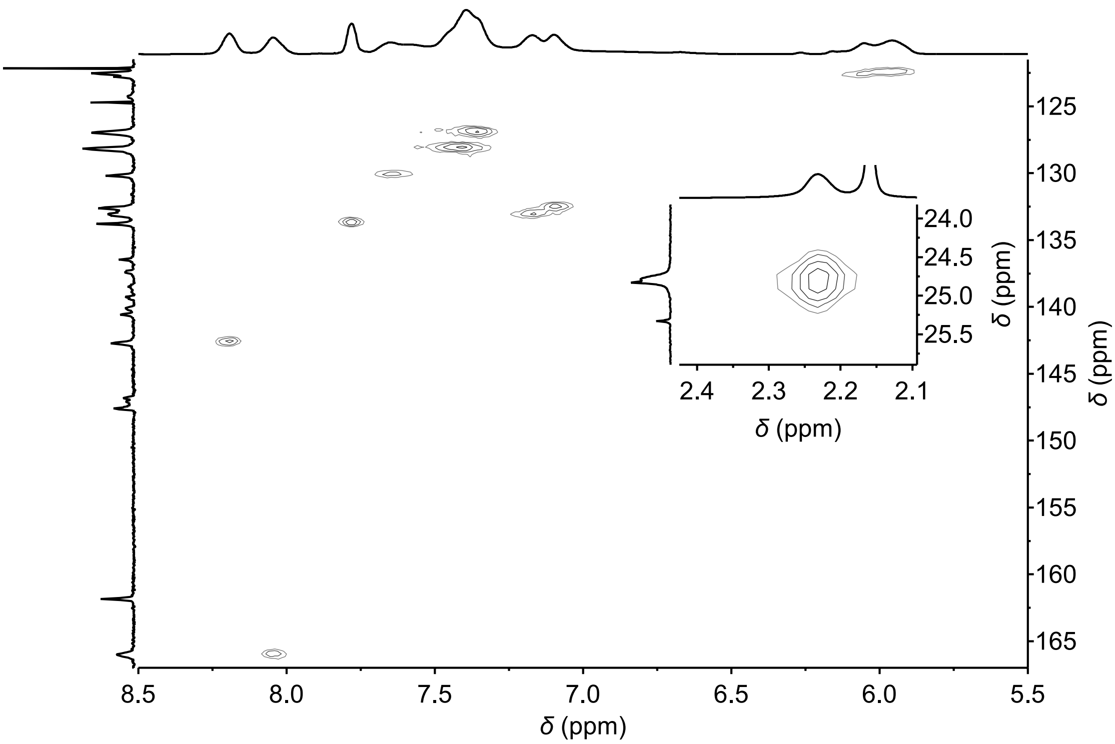

Figure S9. Partial ^1H–^{13}C HSQC spectrum (500 MHz, 298 K, CD_3CN) of dodecahedron **Zn-1 [$Zn_{20}L_{12}$](NTf_2)$_{40}$** (Inset shows the correlation of the CH_3 group).

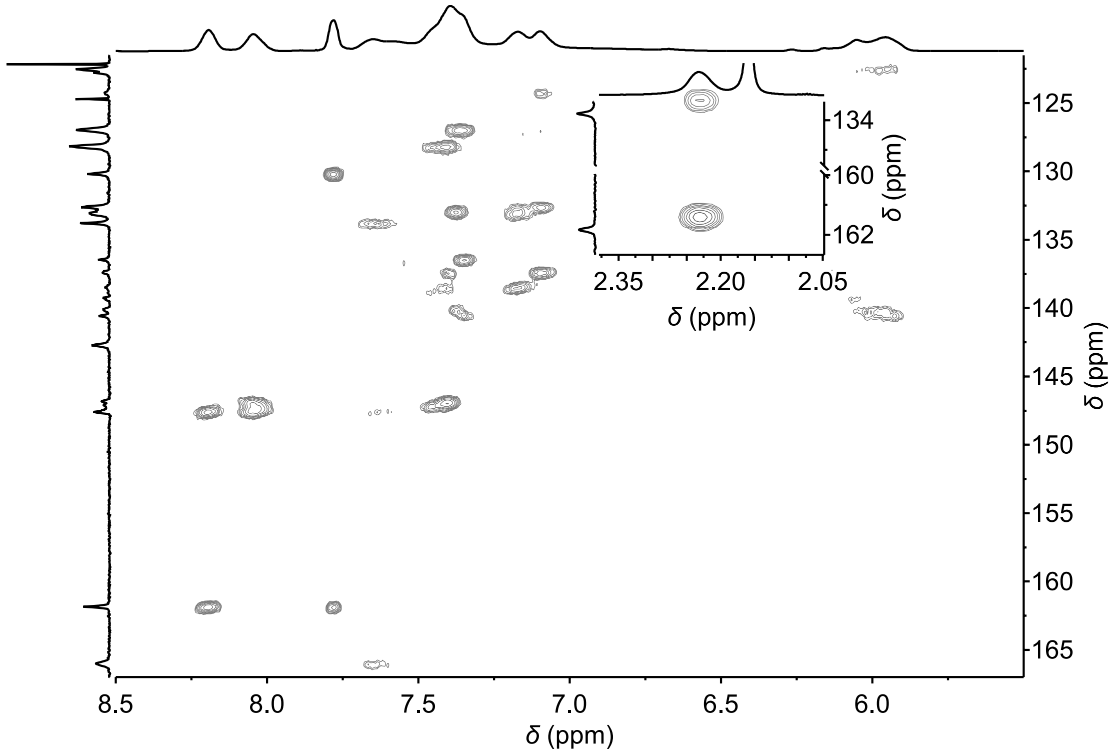

Figure S10. Partial ^1H–^{13}C HMBC spectrum (500 MHz, 298 K, CD_3CN) of dodecahedron **Zn-1 [$Zn_{20}L_{12}$](NTf_2)$_{40}$** (Inset shows the correlation of the CH_3 group).

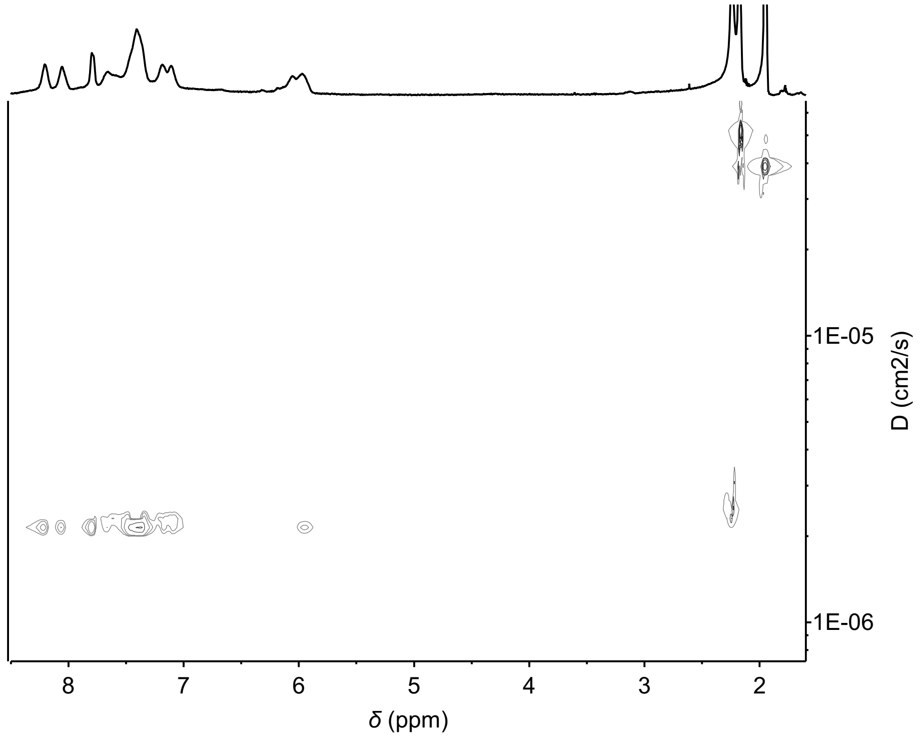

Figure S11. ^{1}H DOSY spectrum (400 MHz, 298 K, CD_3CN) of dodecahedron **Zn-1 [$Zn_{20}L_{12}$](NTf_2)$_{40}$**. Diffusion coefficient: D = 2.19 × 10^{-10} m^2 s^{-1}, r = 27.0 Å.

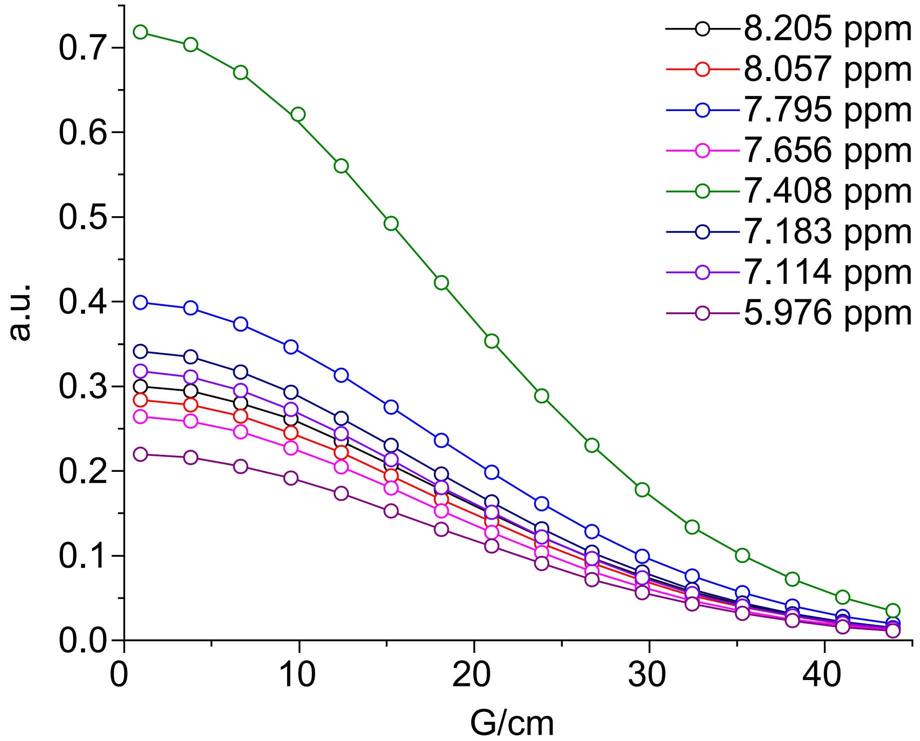

Figure S12. T_1 fitting of the DOSY spectrum (400 MHz, 298 K, CD_3CN) of dodecahedron **Zn-1 [$Zn_{20}L_{12}$](NTf_2)$_{40}$**.

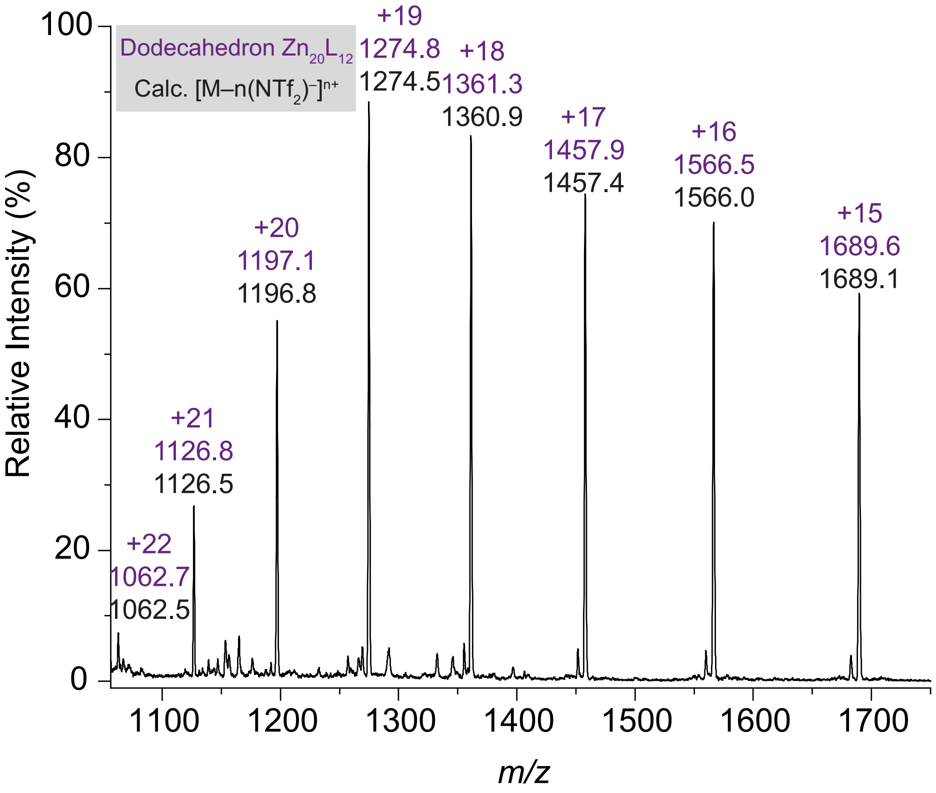

Figure S13. Low-resolution ESI-MS of **Zn-1 $[Zn_{20}L_{12}](NTf_2)_{40}$**.

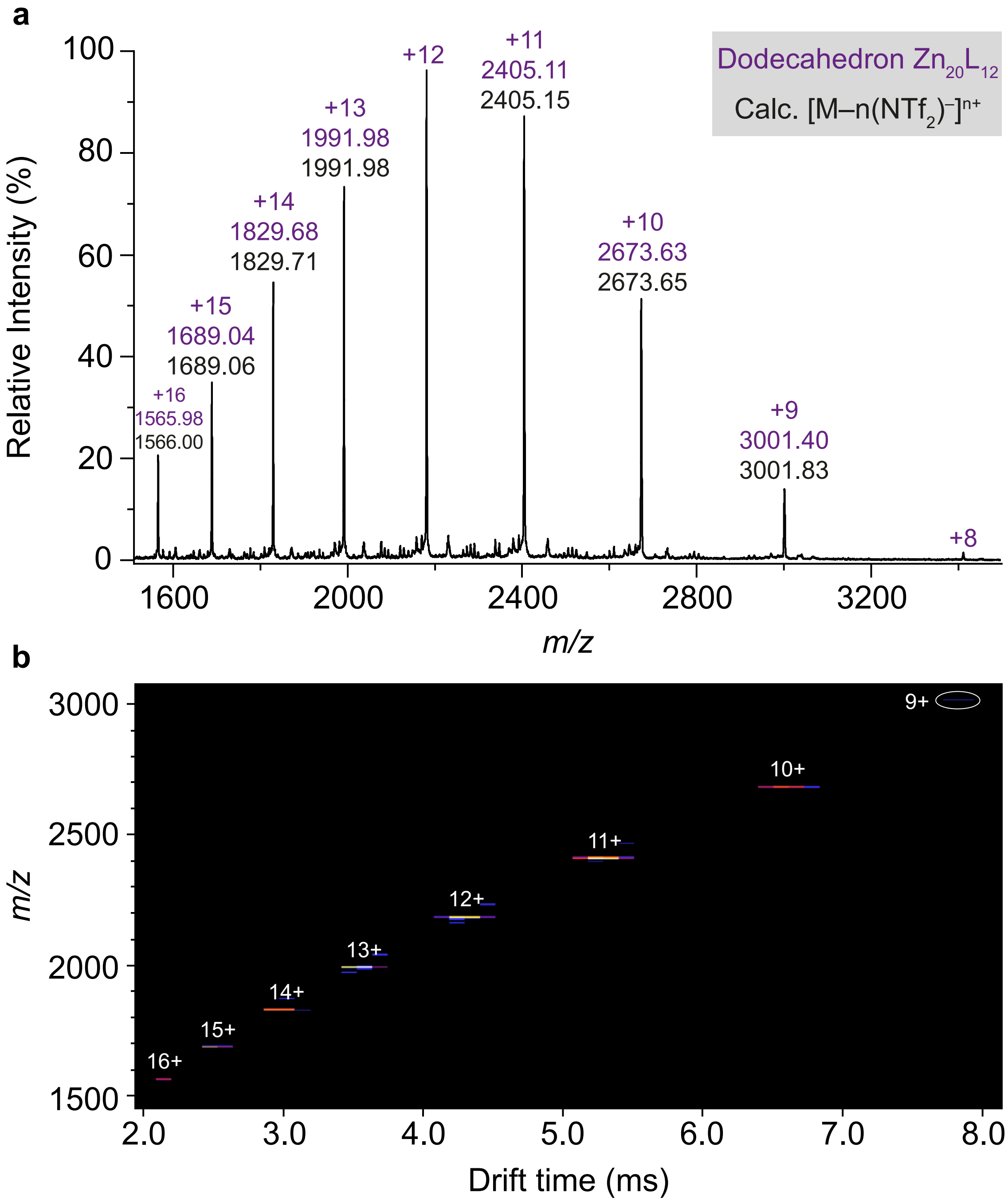

Figure S14. a, HR-ESI-MS of **Zn-1 [$Zn_{20}L_{12}$](NTf_2)$_{40}$**. **b**, 2D ESI-TWIM-MS plot (*m/z* vs. drift time).

3.2. Self-assembly of cages with tridentate-armed ligands

3.2.1. Templated self-assembly of Truncated Rhombohedron **G⊂Zn-3**

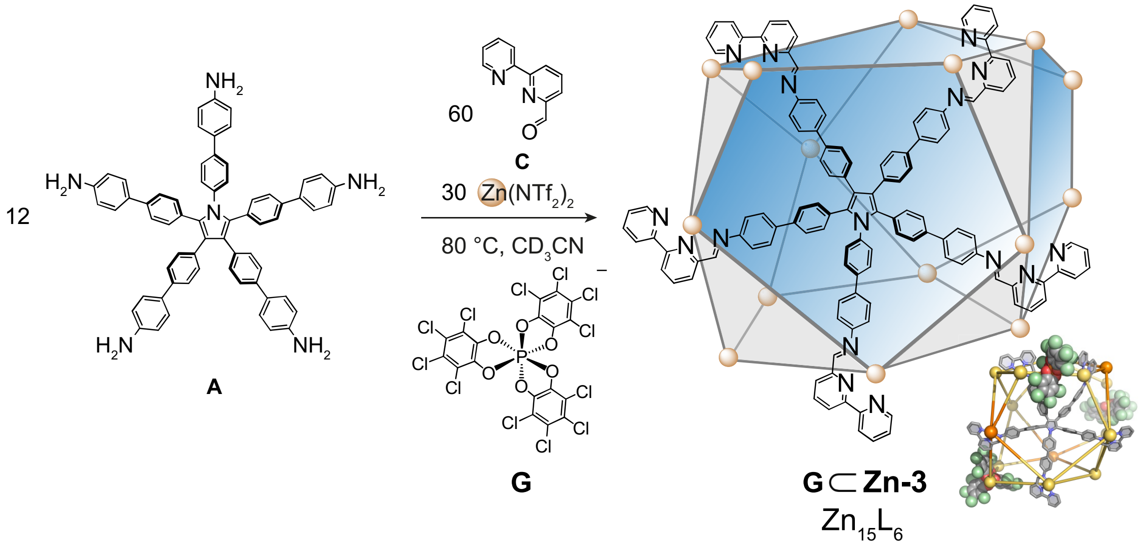

Subcomponent **A** (1.35 mg, 1.5 μmol), $Zn(NTf_2)_2$ (2.4 mg, 3.8 μmol), [2,2'-bipyridine]-6-carbaldehyde **C** (1.4 mg, 7.5 μmol), **G** (2.27 mg, 2.25 μmol) and CD_3CN (0.45 mL) were added to a NMR tube, and the mixture was sonicated for 1 min and heated at 80 °C overnight. The orange-brown solution was concentrated by blowing with N_2 and then Et_2O was added. The resulting solid was collected by centrifugation, washed three times with additional Et_2O and then vacuum dried to give of truncated rhombohedron **G⊂Zn-3** as a brown solid.

^{1}H NMR (500 MHz, CD_3CN) *δ* 9.8 (br), 9.7 (br), 9.5 (br), 9.4 (br), 9.0 (br), 8.9 (br), 8.8 (br), 8.7 (br), 8.6 (br), 8.6 (br), 8.6 (br), 8.6 (br), 8.5 (br), 8.4 (br), 8.3 (br), 8.2 (br), 7.8 (br), 7.7 (br), 7.6 (br), 7.5 (br), 7.4 (br), 7.4 (br), 7.3 (br), 7.3 (br), 7.2 (br), 7.1 (br), 6.9 (br), 6.9 (br, 4H), 6.8 (br, 4H), 6.6 (br), 6.5 (br), 6.3 (br, 7H), 6.1 (br), 6.0 (br).

^{13}C NMR (126 MHz, CD_3CN) *δ* 159.5, 150.8, 150.4, 149.9, 149.5, 149.3, 149.0, 148.7, 148.0, 148.0, 147.7, 147.6, 147.4, 146.9, 146.8, 146.2, 146.0, 145.8, 145.4, 145.3, 145.1, 145.0, 142.9, 142.8, 142.1, 133.2, 132.9, 132.6, 132.5, 132.2, 130.9, 130.2, 129.4, 129.1, 128.4, 128.1, 127.3, 126.9, 125.0, 124.8, 124.7, 123.7, 123.0, 122.2, 114.7, 114.5.

^{19}F NMR (376 MHz, CD_3CN) *δ* –80.05 (s, CF_3).

^{31}P NMR (162 MHz, CD_3CN) *δ* –81.33.

High-resolution ESI-MS (CH_3CN): *m/z*: 1397.31 $[M-15NTf_2^-]^{15+}$, 1517.12 $[M-14NTf_2^-]^{14+}$, 1655.38 $[M-13NTf_2^-]^{13+}$, 1816.66 $[M-12NTf_2^-]^{12+}$, 2007.29 $[M-11NTf_2^-]^{11+}$, 2236.01 $[M-10NTf_2^-]^{10+}$, 2515.61 $[M-9NTf_2^-]^{9+}$.

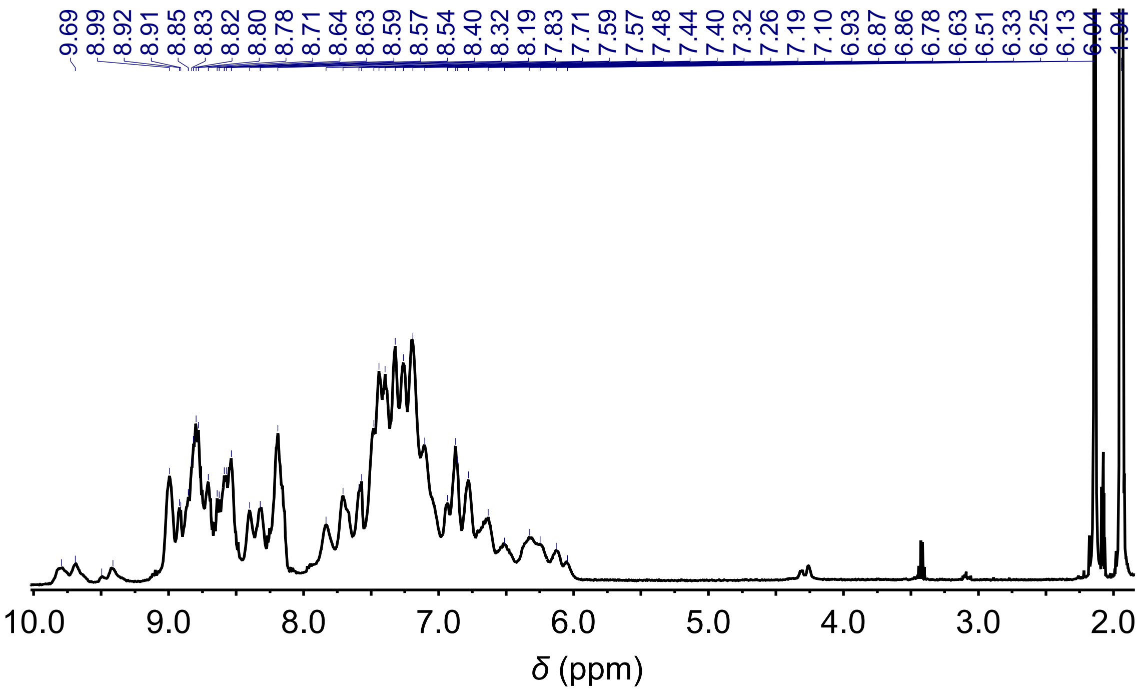

Figure S15. ^{1}H NMR spectrum (500 MHz, 298 K, CD_3CN) of truncated rhombohedron **G⊂Zn-3**. Several sets of broad signals of the ligand arms were observed presumably due to the different ligand arm environments as a result of the low symmetry, different Zn^{II} handedness, and pyrrole-N random orientation, resulting in signal overlap and broadness.

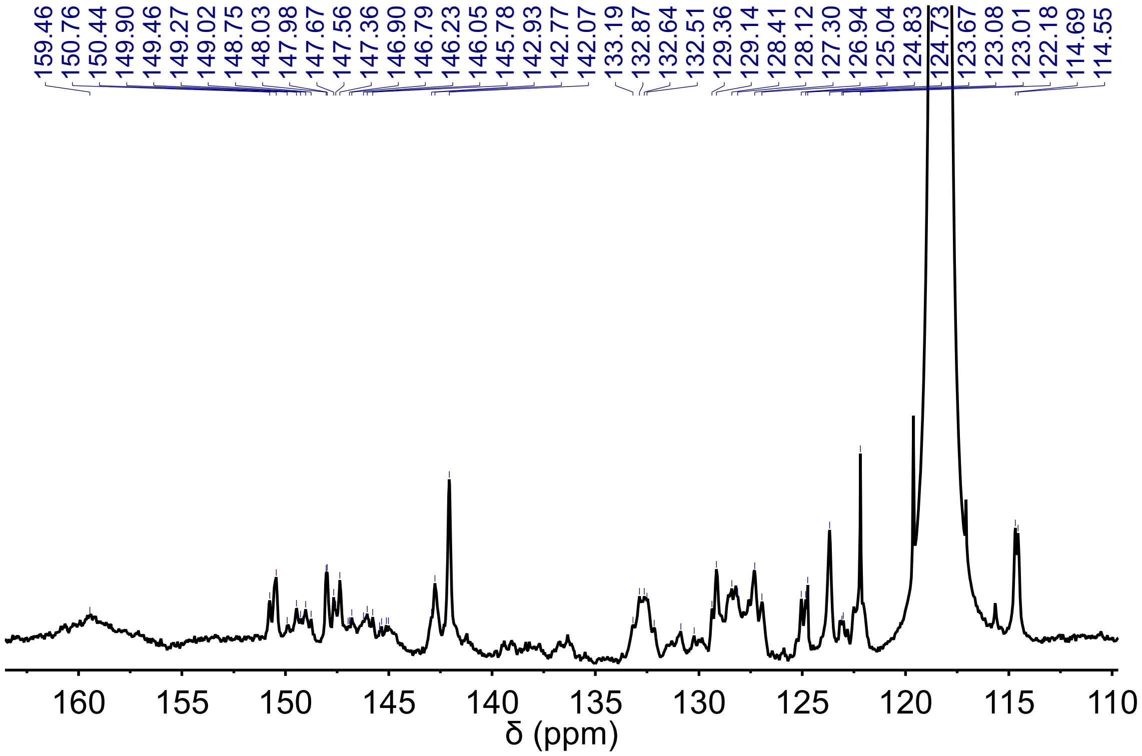

Figure S16. ^{13}C NMR spectrum (126 MHz, 298 K, CD_3CN) of truncated rhombohedron

G⊂Zn-3. ^{13}C peaks are broad presumably due to the many isomers in the system caused by rotationally disordered pyrrole-N and the slow tumbling of the large cage molecules in the solution, thus fewer than the expected number of signals are observed.

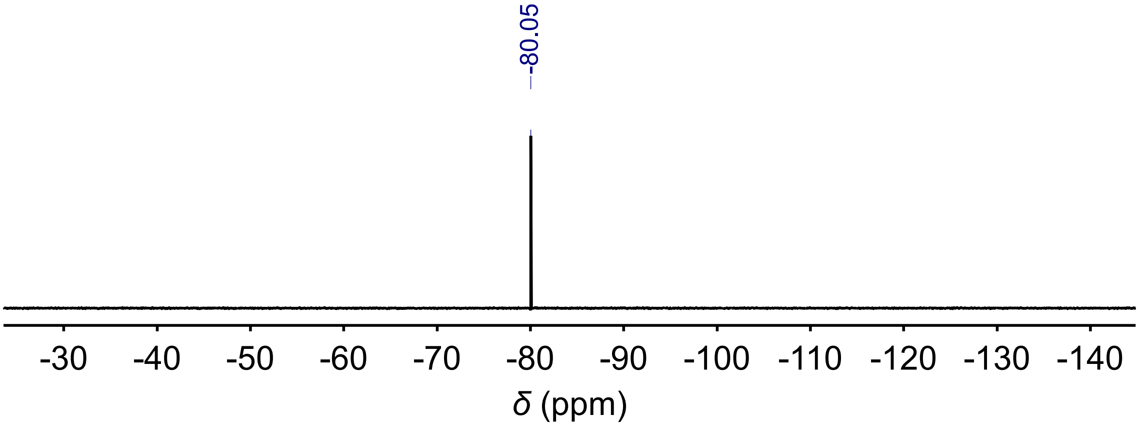

Figure S17. ^{19}F NMR spectrum (376 MHz, 298 K, CD_3CN) of truncated rhombohedron **G⊂Zn-3**.

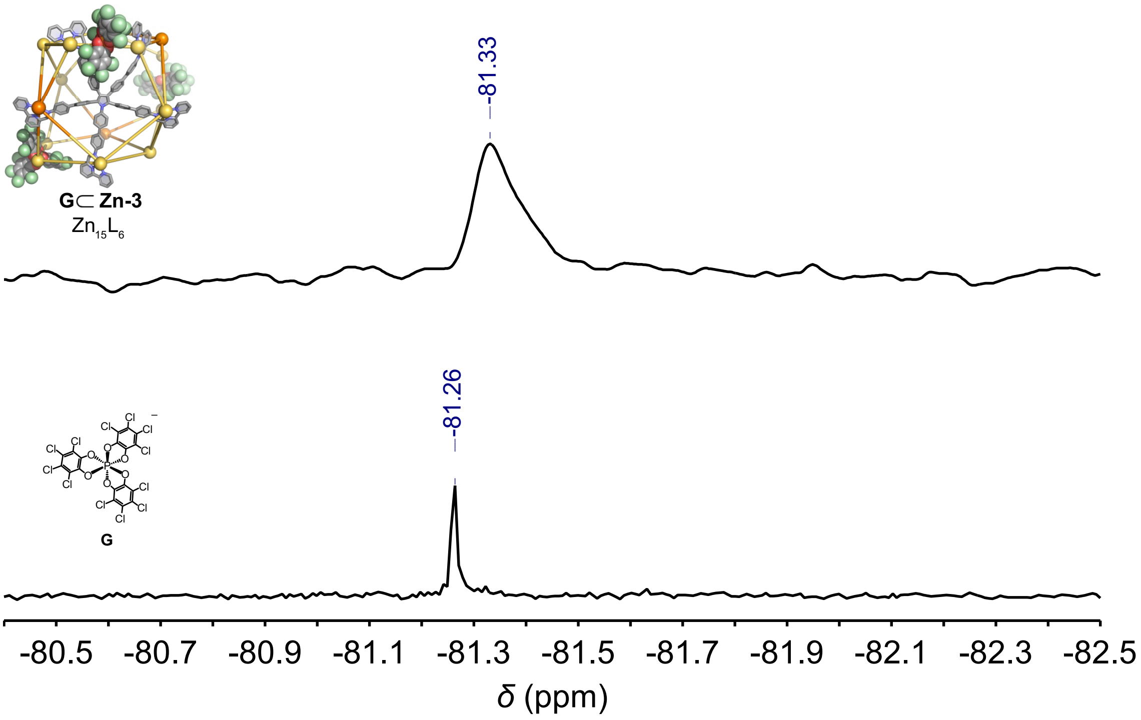

Figure S18. ^{31}P spectrum (162 MHz, 298 K, CD_3CN) of truncated rhombohedron **G⊂Zn-3** (top) and **G** (bottom).

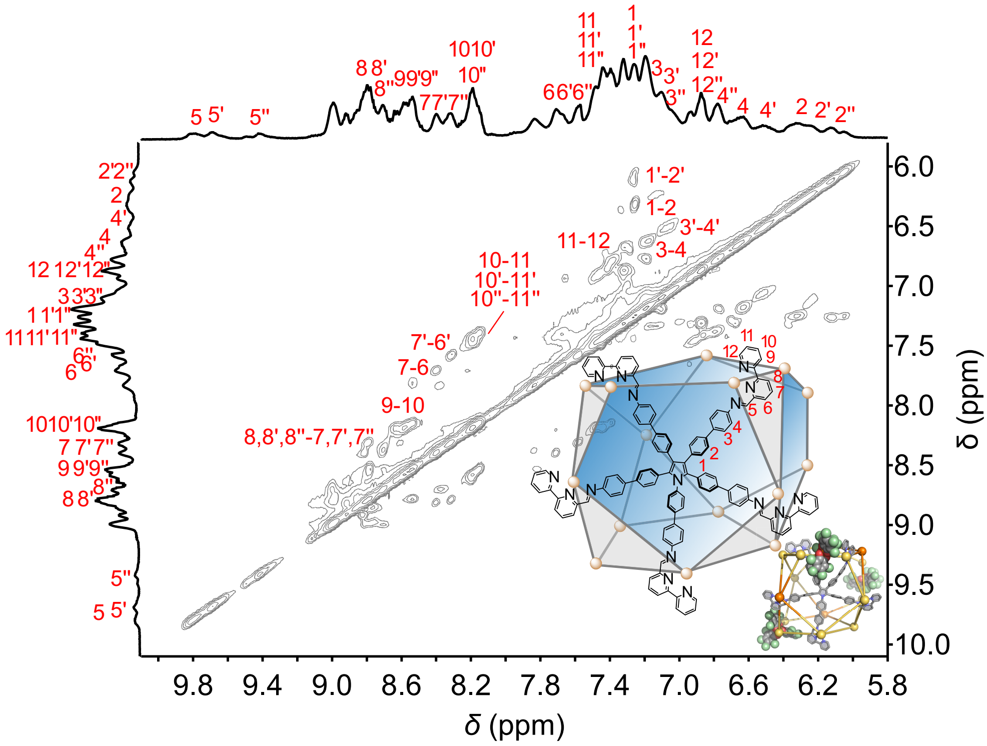

Figure S19. Partial ^{1}H–^{1}H COSY spectrum (500 MHz, 298 K, CD_3CN) of truncated rhombohedron **G⊂Zn-3**.

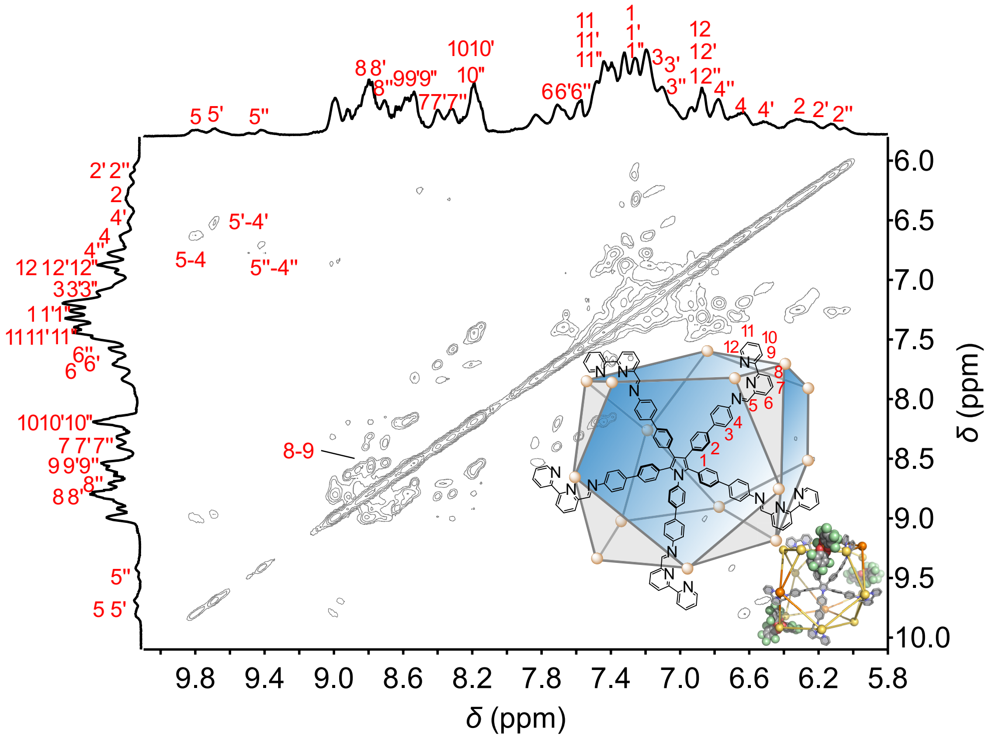

Figure S20. Partial ^{1}H–^{1}H NOESY spectrum (500 MHz, 298 K, CD_3CN) of truncated rhombohedron **G⊂Zn-3**.

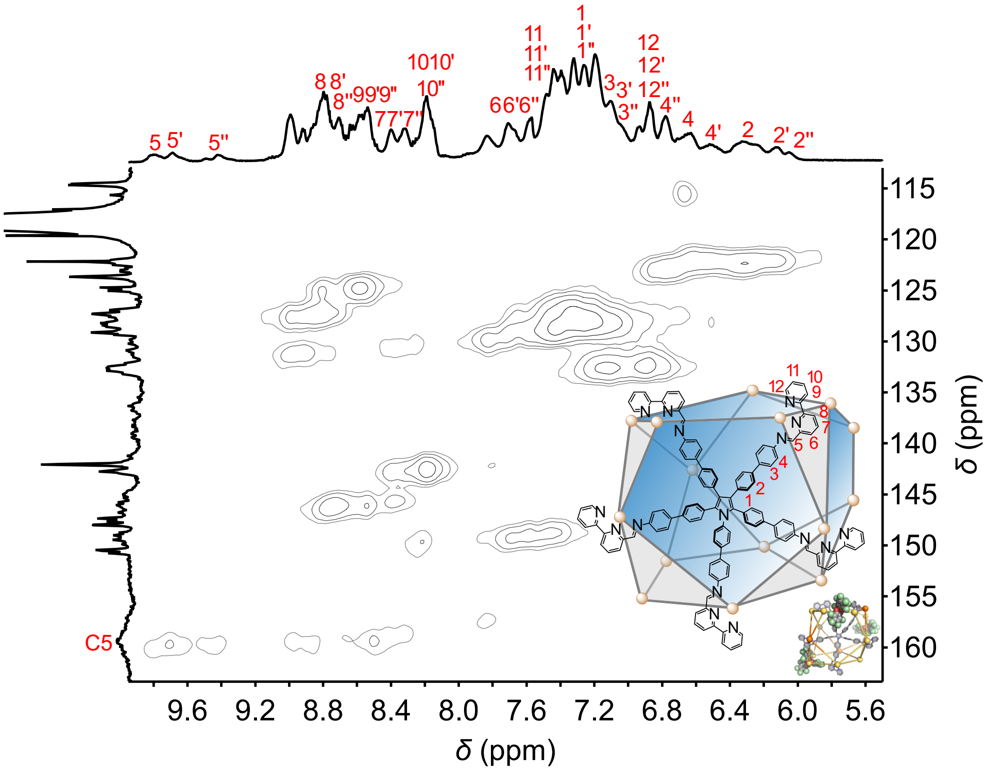

Figure S21. Partial ^{1}H–^{13}C HSQC spectrum (500 MHz, 298 K, CD_3CN) of truncated rhombohedron **G⊂Zn-3**.

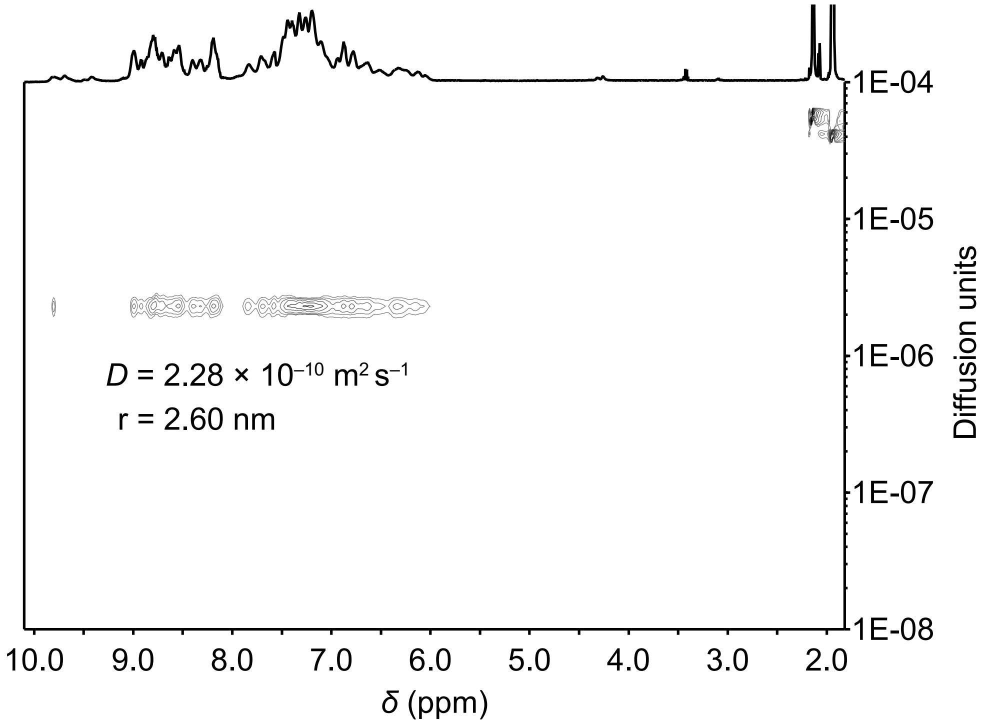

Figure S22. ^{1}H DOSY spectrum (400 MHz, 298 K, CD_3CN) of truncated rhombohedron **G⊂Zn-3**. Diffusion coefficient: $D = 2.28 \times 10^{-10}$ m^2 s^{-1}, r = 26.0 Å.

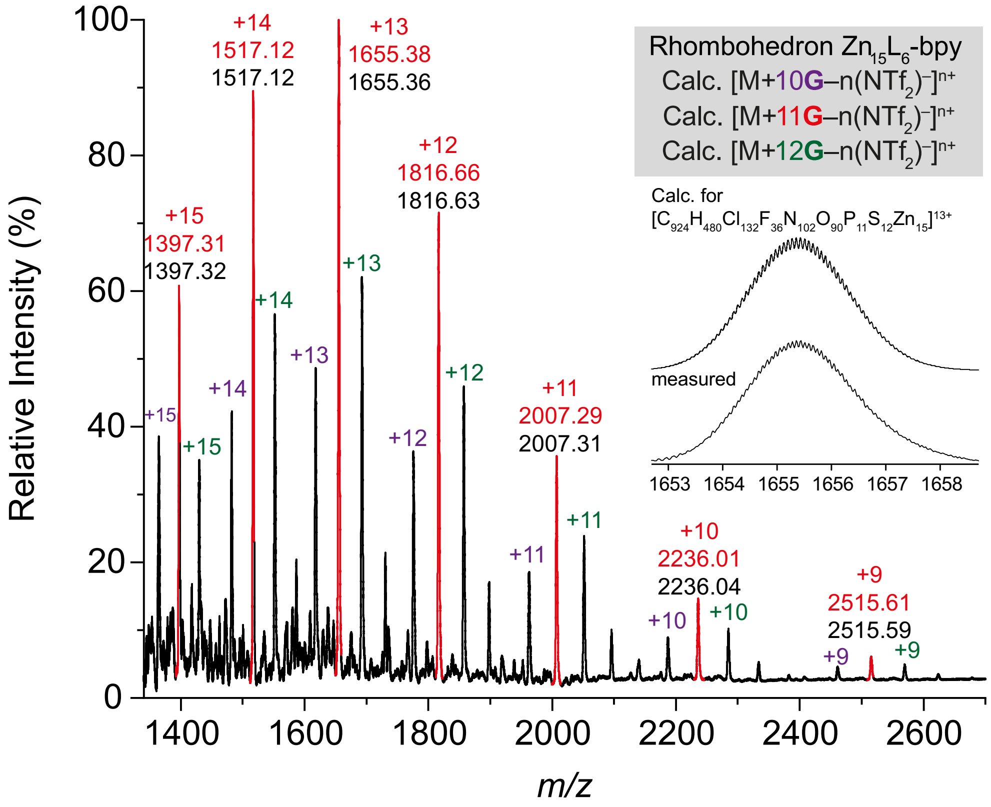

Figure S23. HR-ESI-MS of truncated rhombohedron **G⊂Zn-3**.

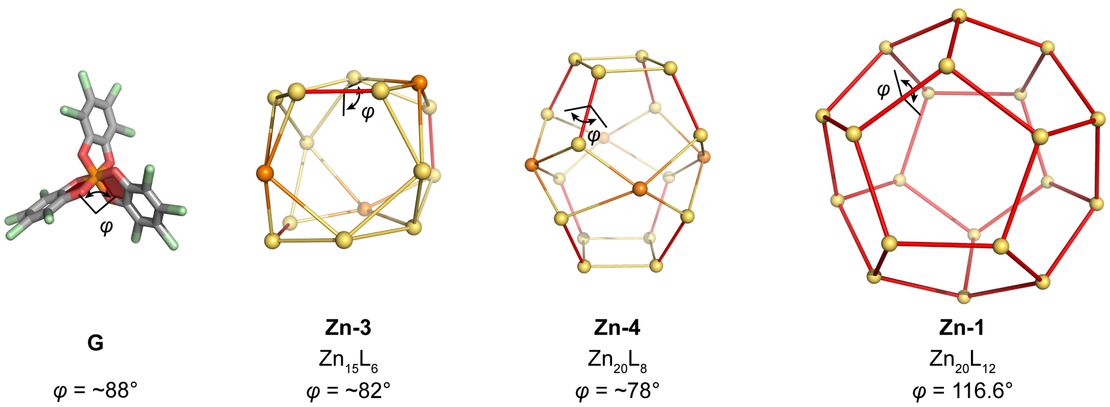

Figure S24. Comparison of the dihedral angles of cages and **G**. Only the truncated octahedral structure **Zn-4** and rhombohedron **Zn-3** match with that of **G**. (Common edges where two pentagonal faces share are colored with red)

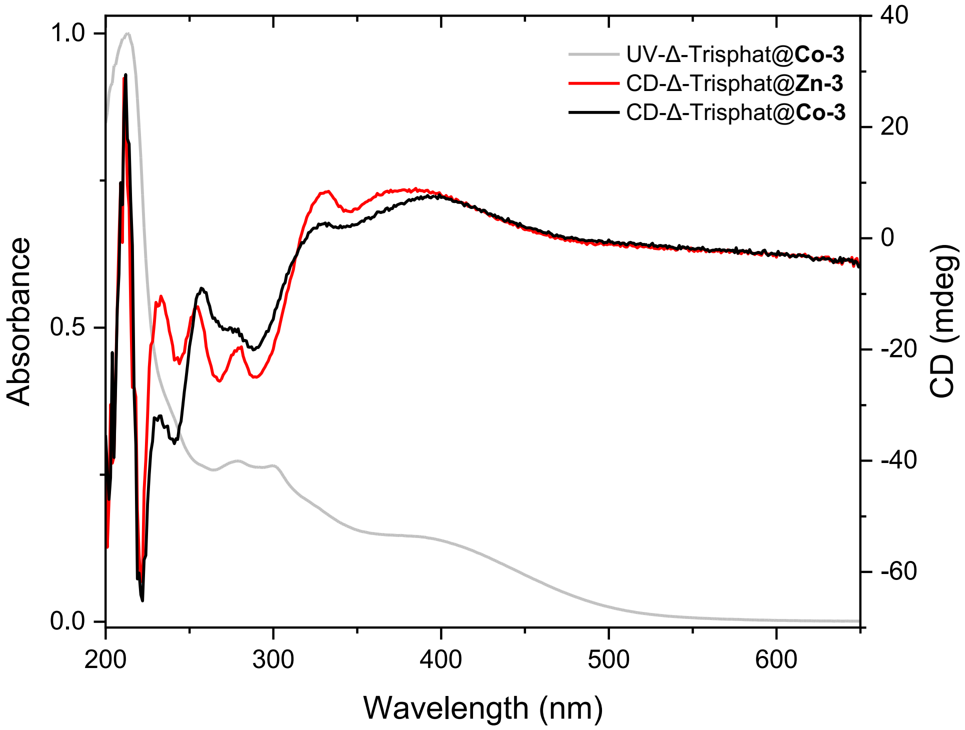

Figure S25. UV-vis and CD spectrum of truncated rhombohedron **G⊂Zn-3** and **G⊂Co-3**.

3.2.2. Templated self-assembly of Truncated Rhombohedron **G⊂Co-3**

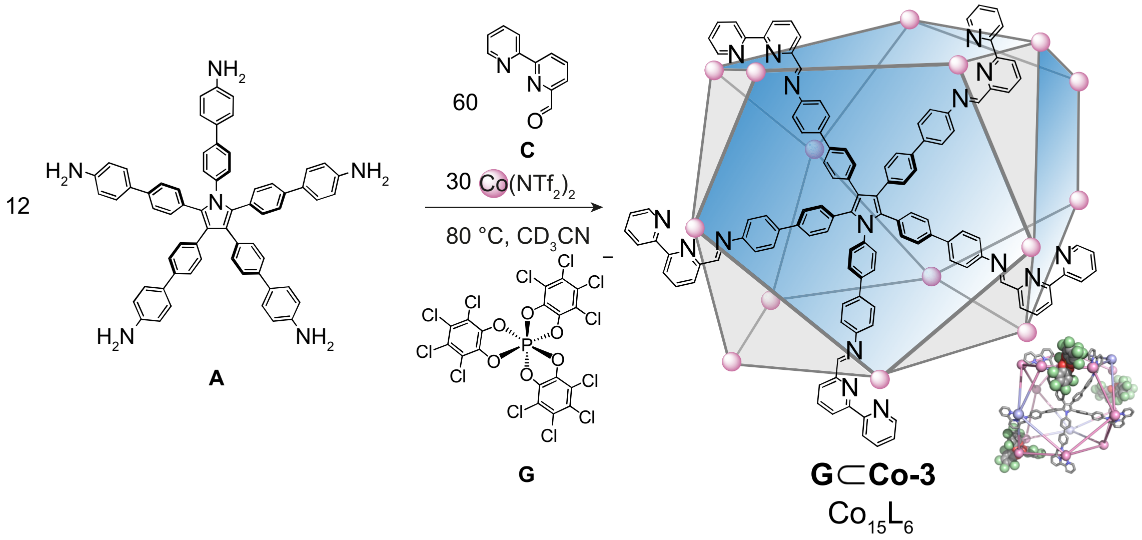

Subcomponent **A** (1.35 mg, 1.5 μmol), $Co(NTf_2)_2$ (2.35 mg, 3.8 μmol), [2,2'-bipyridine]-6-carbaldehyde **C** (1.4 mg, 7.5 μmol), **G** (2.27 mg, 2.25 μmol) and CD_3CN (0.45 mL)

were added to a NMR tube, and the mixture was sonicated for 1 min and heated at 80 °C overnight. The deep orange-brown solution was concentrated by blowing with N_2 and then Et_2O was added. The resulting solid was collected by centrifugation, washed three times with additional Et_2O and then vacuum dried to give of truncated rhombohedron **G⊂Co-3** as a brown solid.

High-resolution ESI-MS (CH_3CN): *m/z*: 1194.20 $[M–17NTf_2^-]^{17+}$, 1286.41 $[M–16NTf_2^-]^{16+}$, 1390.88 $[M–15NTf_2^-]^{15+}$, 1510.13 $[M–14NTf_2^-]^{14+}$, 1647.87 $[M–13NTf_2^-]^{13+}$, 1808.59 $[M–12NTf_2^-]^{12+}$, 1998.41 $[M–11NTf_2^-]^{11+}$, 2226.12 $[M–10NTf_2^-]^{10+}$.

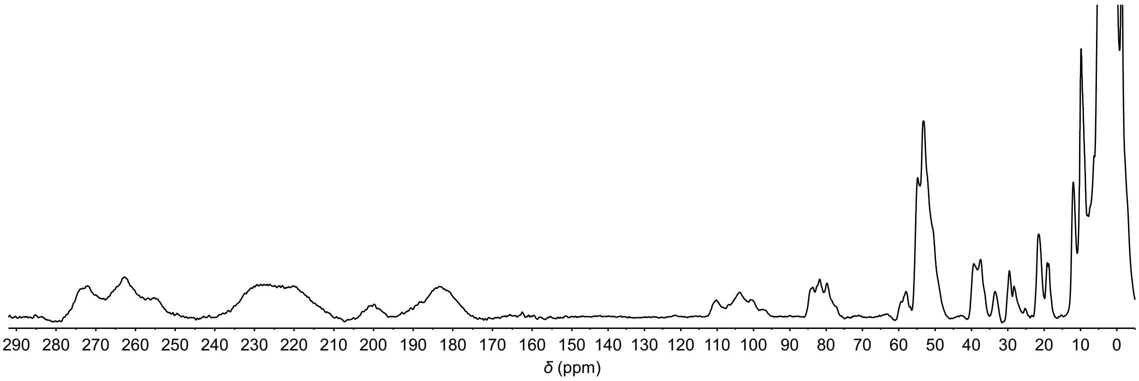

Figure S26. ^1H NMR spectrum (400 MHz, 298 K, CD_3CN) of truncated rhombohedron **G⊂Co-3**. Several sets of broad signals of the ligand arms were observed presumably due to the different ligand arm environments as a result of the low symmetry, different Co^{II} handedness, and pyrrole-N random orientation, resulting in signal overlap and broadness.

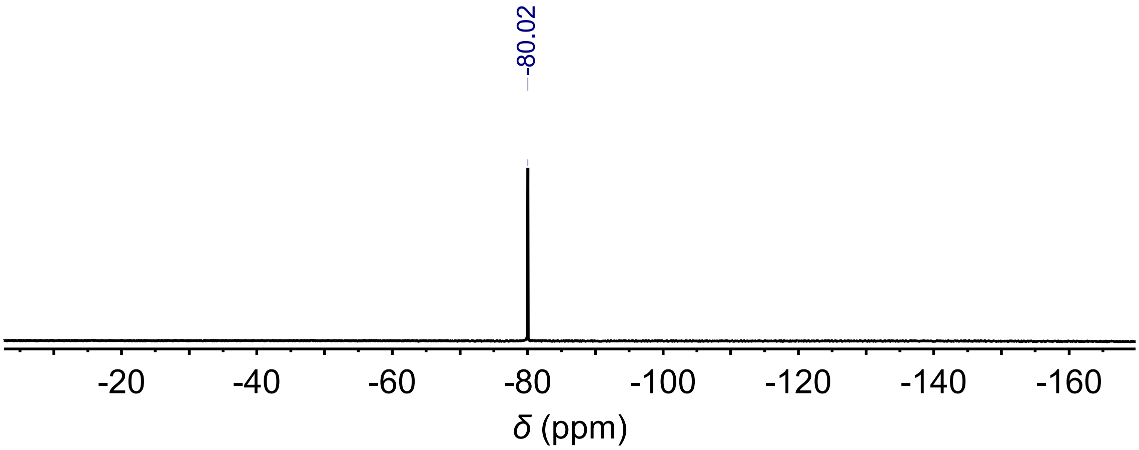

Figure S27. ^{19}F NMR spectrum (376 MHz, 298 K, CD_3CN) of truncated rhombohedron **G⊂Co-3**.

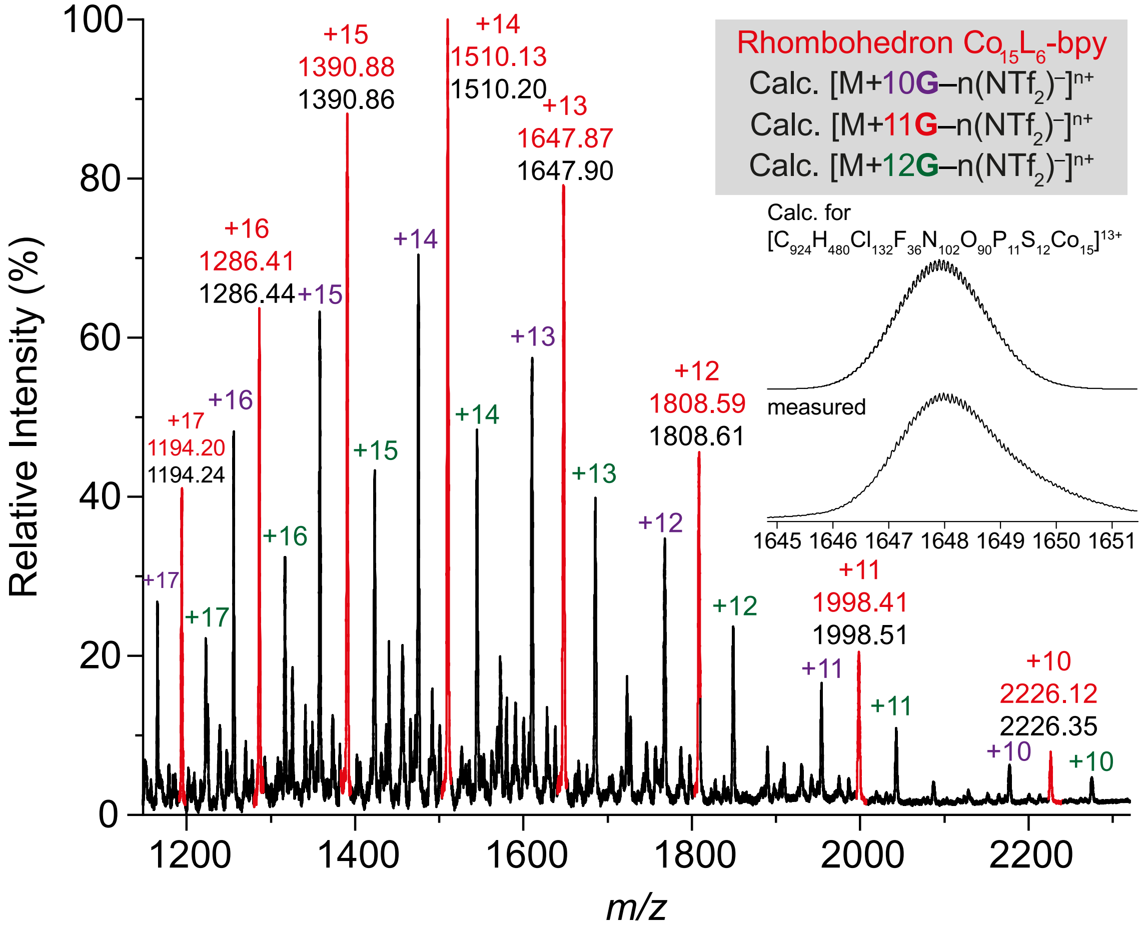

Figure S28. HR-ESI-MS of truncated rhombohedron **G⊂Co-3**.

3.2.3. Self-assembly of Sandwich structure **Zn-5**

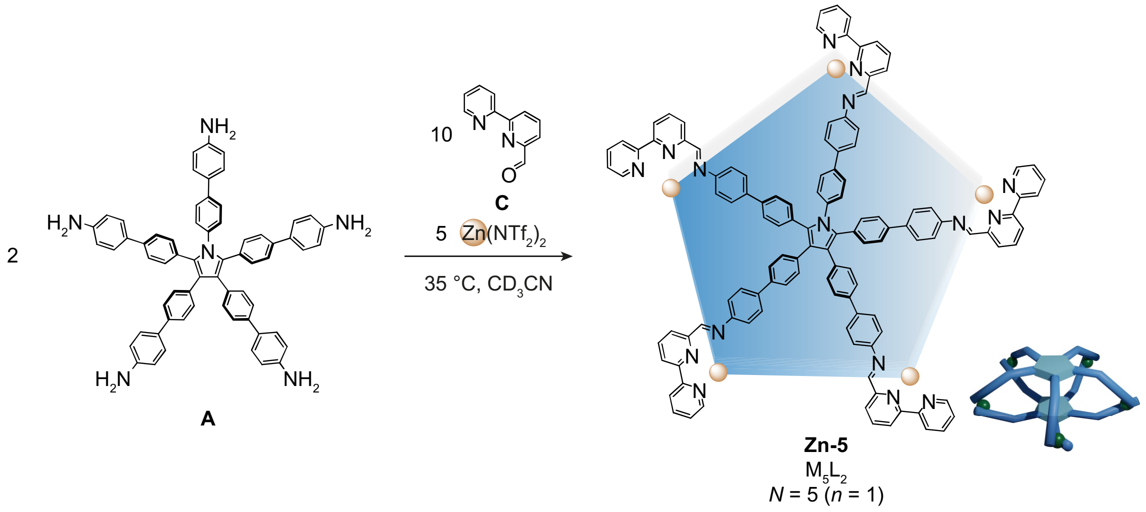

Subcomponent **A** (0.90 mg, 1.0 μmol), $Zn(NTf_2)_2$ (1.6 mg, 2.5 μmol), [2,2'-bipyridine]-6-carbaldehyde **C** (0.91 mg, 5 μmol) and CD_3CN (0.4 mL) were added to a NMR tube, and the mixture was sonicated for 1 min and heated at 35 °C for 0.5 h. The pale orangish

solution was concentrated by blowing with N_2 and then Et_2O was added. The resulting solid was collected by centrifugation, washed three times with additional Et_2O and then vacuum dried to give sandwich **Zn-5 $[Zn_5L_2](NTf_2)_{10}$** as a brown solid (2.97 mg, 0.45 μmol, yield 90%).

^{1}H NMR (400 MHz, CD_3CN) δ 9.2 – 9.0 (br), 8.8 (br), 8.7 (br), 8.6 (br), 8.4 (br), 8.3 (br), 8.3 – 8.2 (br), 7.7 (br), 7.5 (br), 7.5 (br), 6.9 (br), 6.8 (br), 6.6 (br), 6.1 (br), 6.0 (br), 6.0 (br).

^{13}C NMR (176 MHz, CD_3CN) δ 160.0, 157.9, 151.0, 150.4, 149.9, 149.3, 149.0, 148.7, 148.1, 147.8, 147.2, 145.9, 144.5, 143.3, 142.6, 132.8, 132.4, 130.4, 129.0, 128.6, 127.4, 126.8, 126.5, 124.9, 124.7, 124.2, 123.5, 123.0, 122.3, 77.3.

^{19}F NMR (371 MHz, CD_3CN) δ –80.08 (s, CF_3).

High-resolution ESI-MS (CH_3CN): *m/z*: 662.09 $[M–7NTf_2^{-}]^{7+}$, 819.26 $[M–6NTf_2^{-}]^{6+}$, 1053.51 $[M+4H_2O–5NTf_2^{-}]^{5+}$, 1391.36 $[M+5H_2O–4NTf_2^{-}]^{4+}$, 1948.46 $[M+5H_2O–3NTf_2^{-}]^{3+}$.

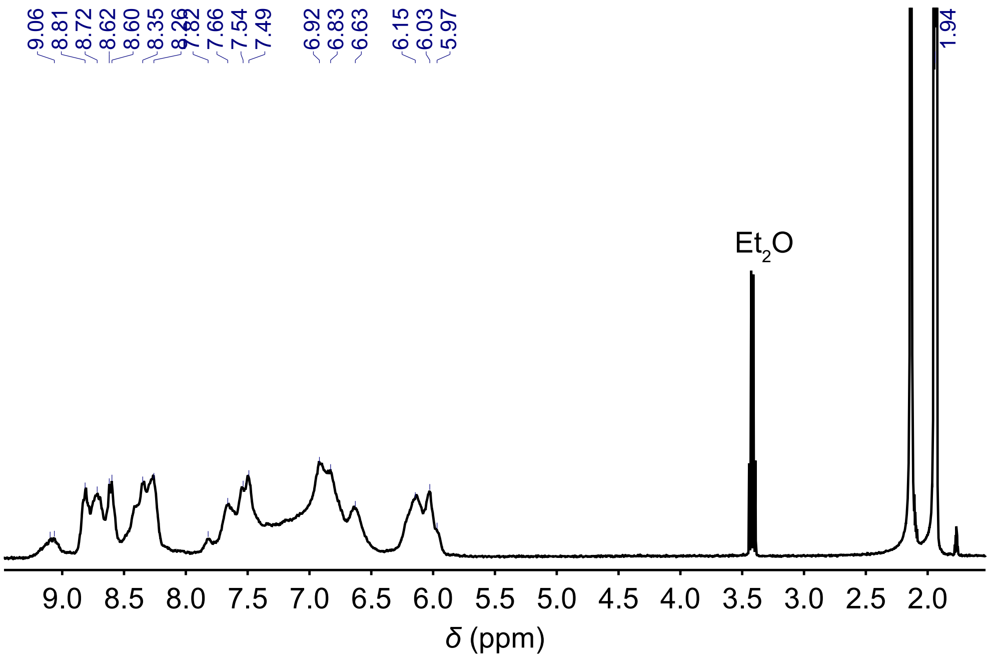

Figure S29. ^{1}H NMR spectrum (400 MHz, 298 K, CD_3CN) of **Zn-5 $[Zn_5L_2](NTf_2)_{10}$**. Only broad signals of the ligand arms were observed presumably due to the different ligand arm environments as a result of pyrrole-N random orientation are similar, resulting in signal overlap and broadness.

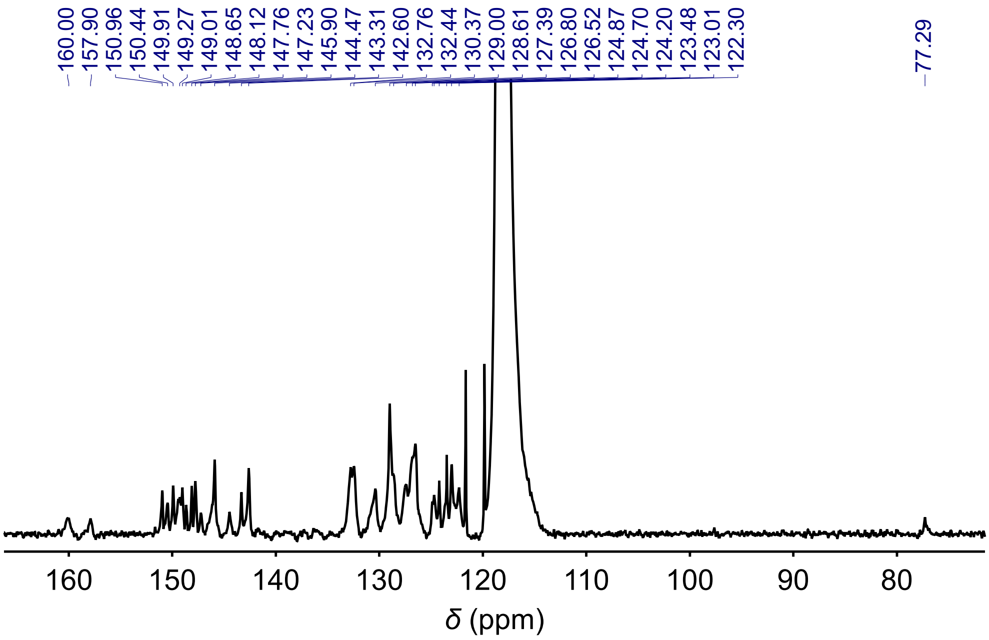

Figure S30. ^{13}C NMR spectrum (176 MHz, 298 K, CD_3CN) of **Zn-5 $[Zn_5L_2](NTf_2)_{10}$**. ^{13}C peaks are broad presumably due to the many isomers in the system caused by rotationally disordered pyrrole-N and the slow tumbling of the cage molecules in the solution, thus fewer than the expected number of signals are observed.

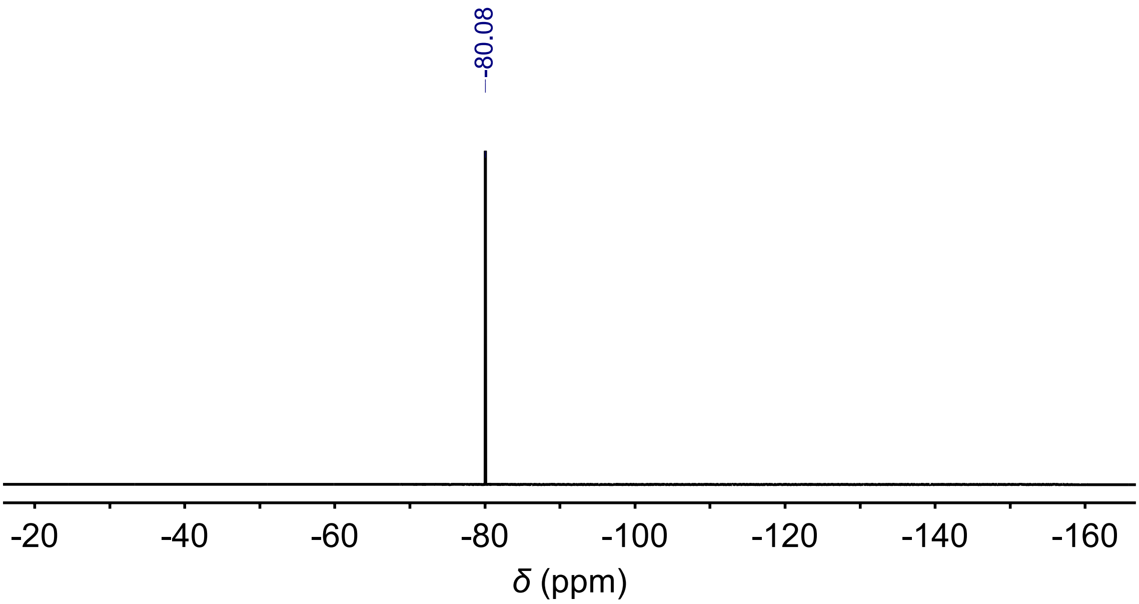

Figure S31. ^{19}F NMR spectrum (376 MHz, 298 K, CD_3CN) of **Zn-5 $[Zn_5L_2](NTf_2)_{10}$**.

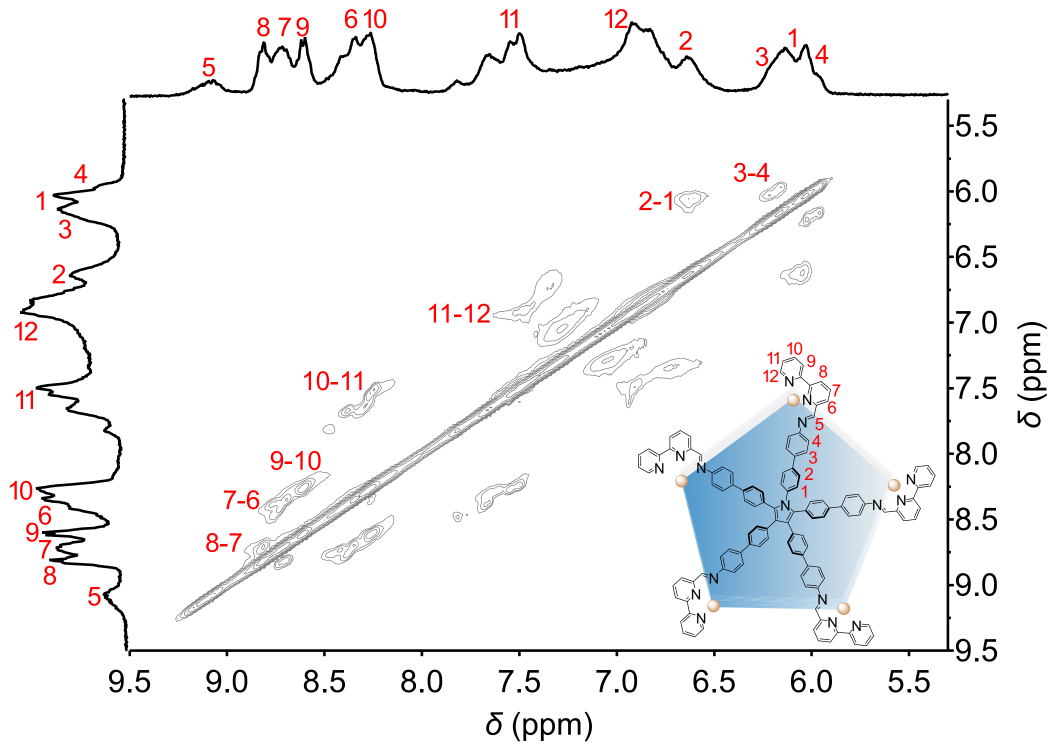

Figure S32. Partial ^1H–^1H COSY spectrum (500 MHz, 298 K, CD_3CN) of **Zn-5 [Zn_5L_2](NTf_2)$_{10}$**.

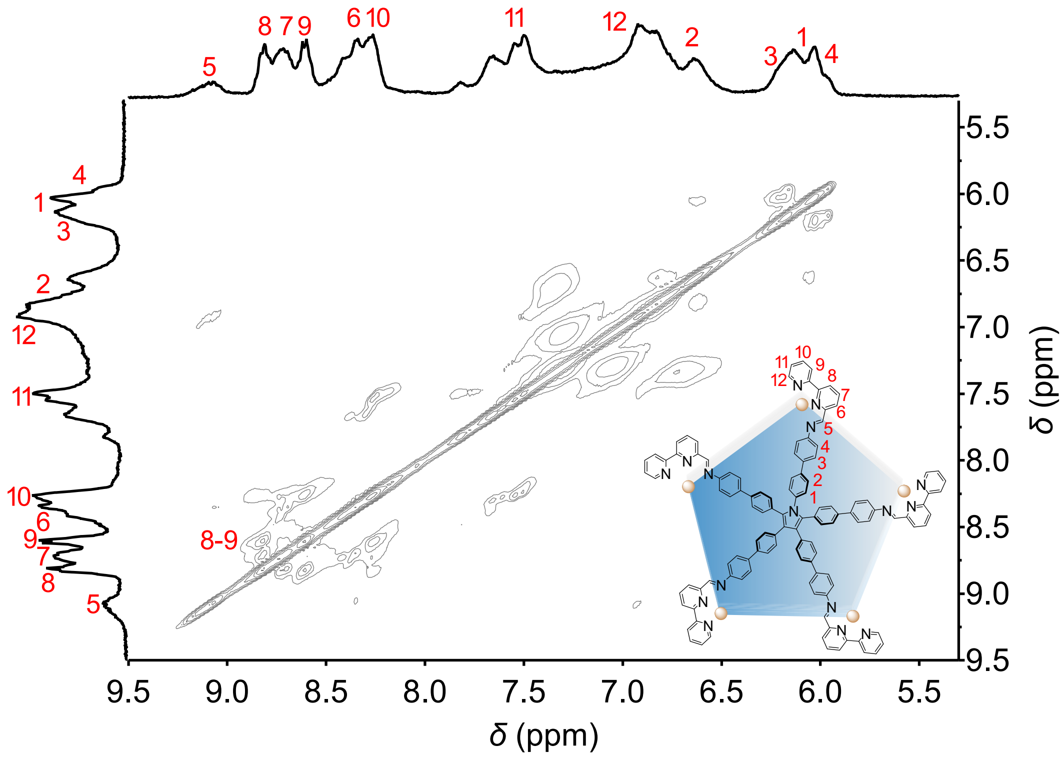

Figure S33. Partial ^1H–^1H NOESY spectrum (500 MHz, 298 K, CD_3CN) of **Zn-5 [Zn_5L_2](NTf_2)$_{10}$**.

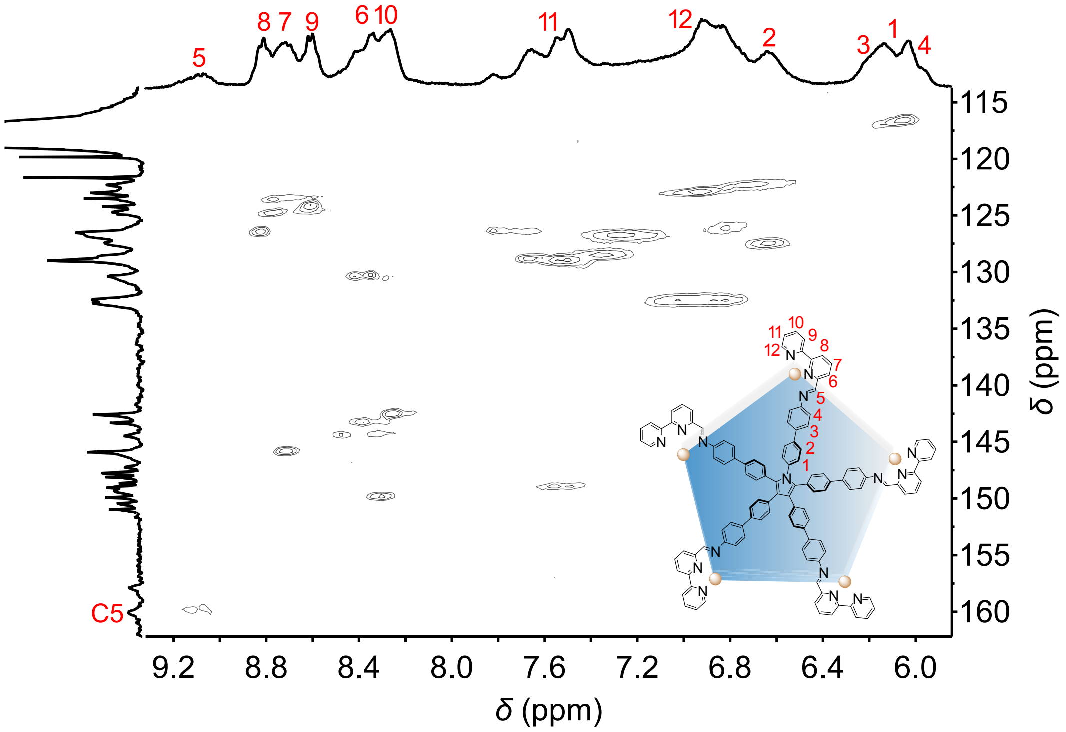

Figure S34. Partial ^{1}H–^{13}C HSQC spectrum (500 MHz, 298 K, CD_3CN) of **Zn-5 $[Zn_5L_2](NTf_2)_{10}$**.

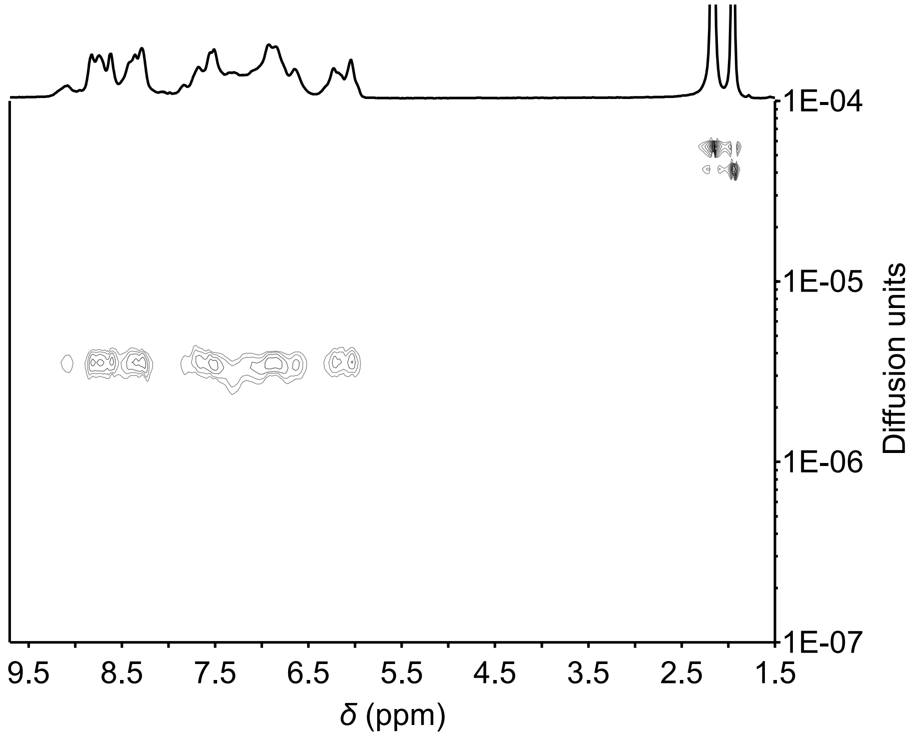

Figure S35. ^{1}H DOSY spectrum (400 MHz, 298 K, CD_3CN) of sandwich **Zn-5 $[Zn_5L_2](NTf_2)_{10}$**. Diffusion coefficient: $D = 3.58 \times 10^{-10}$ m^2 s^{-1}, r = 16.5 Å. r_{model} = 18 Å.

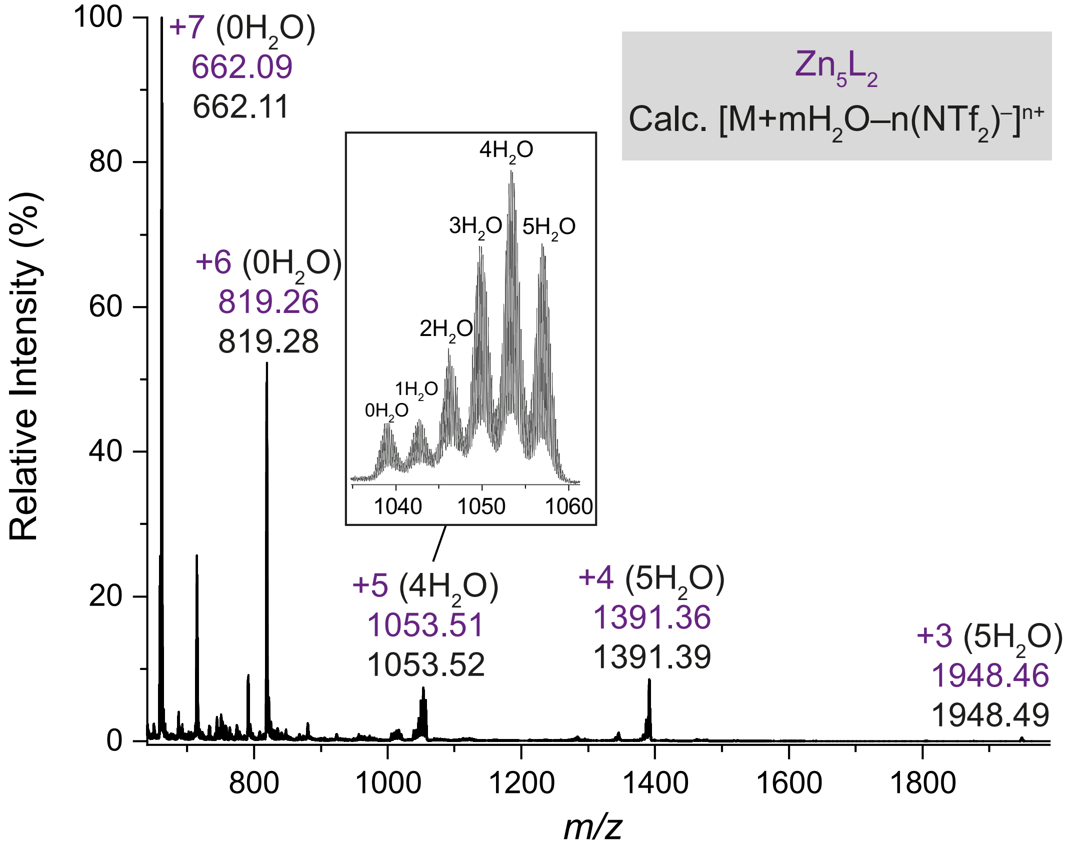

Figure S36. HR-ESI-MS of **Zn-5 [Zn_5L_2](NTf_2)$_{10}$**.

4. Sequential transformations of self-assembled cages

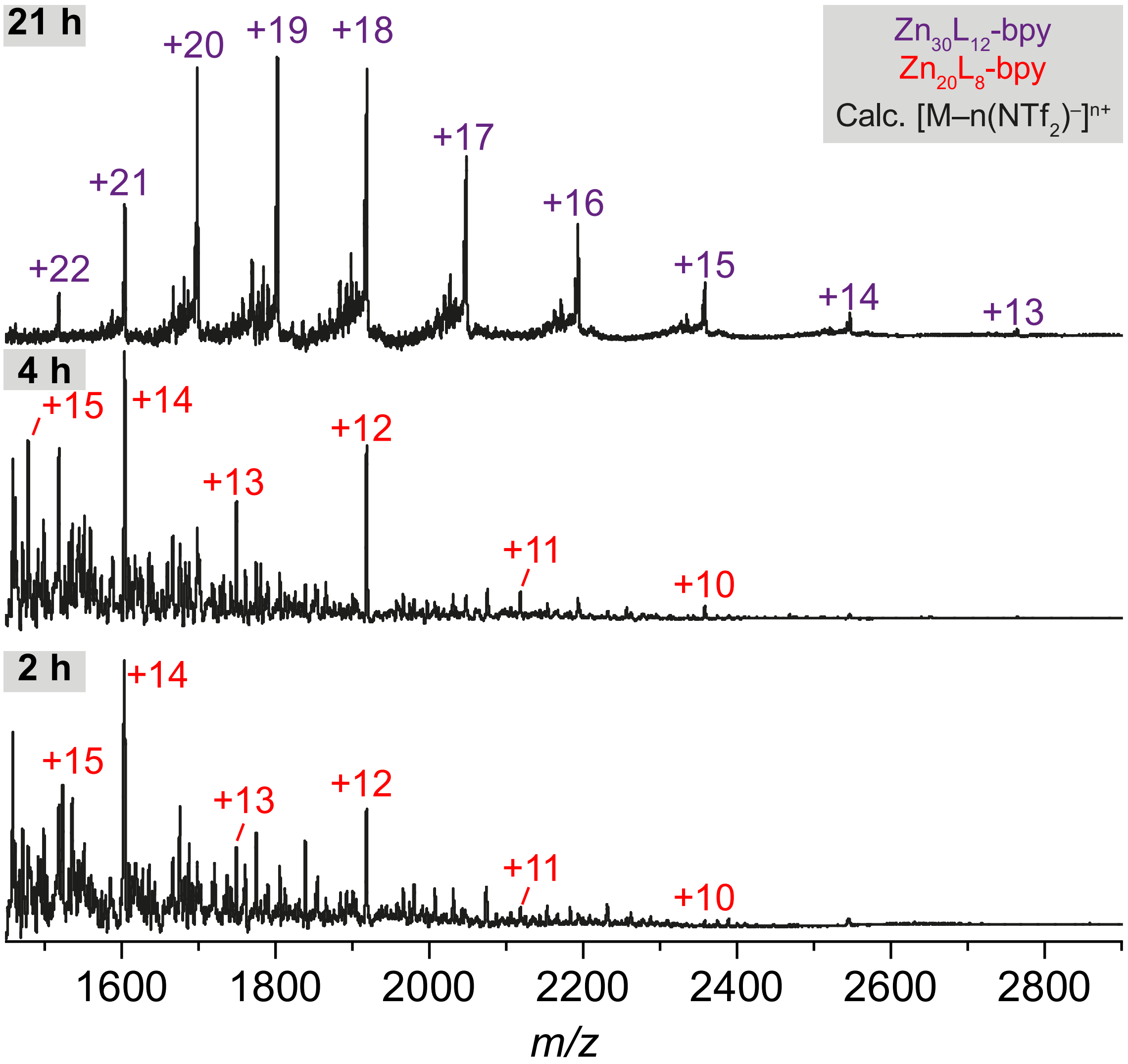

Figure S37. High-resolution ESI-MS monitoring the formation of **Zn-2** **$[Zn_{30}L_{12}](NTf_2)_{60}$** with different time intervals of heating at 80 °C. Signals of **$[Zn_{20}L_8](NTf_2)_{60}$** could be found at early stage of heating before reaching thermodynamic equilibrium.

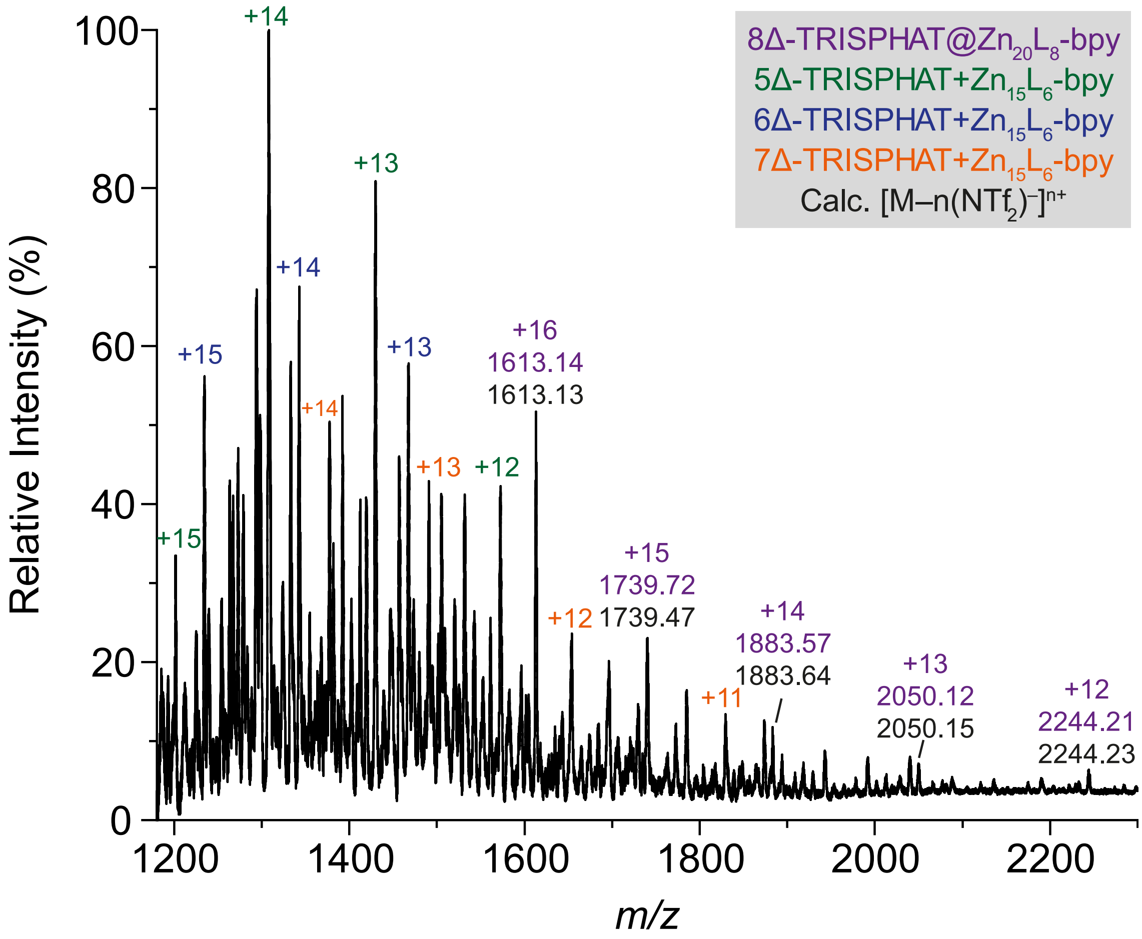

Figure S38. High-resolution ESI-MS of the redissolved crystal samples of **Zn-4** **[Zn$_{20}$L$_8$](NTf$_2$)$_{40}$** (Crystals were grown from **Zn-2** **[Zn$_{30}$L$_{12}$](NTf$_2$)$_{60}$** cage in the presence of **G**). Eight equivalents of **G** were found together with **Zn-4**, consistent with the crystal structure, where eight **G** molecules pack tightly in the window pockets that pentagonal faces share the same edge. Signals of truncated rhombohedron **Zn-3** **[Zn$_{15}$L$_6$](NTf$_2$)$_{30}$** were also found, indicating **Zn-4** transformed into **Zn-3** upon dissolution.

4.1. Cage-to-cage transformation from **Zn-1** to **Zn-2**

Zn-1 **[Zn$_{20}$L$_{12}$](NTf$_2$)$_{40}$** (0.084 μmol, 1.0 μmol based on ligand) was dissolved in 400 μL CD_3CN. [2,2'-bipyridine]-6-carbaldehyde **C** (0.92 mg, 5.0 μmol), and additional $Zn(NTf_2)_2$ (0.52 mg, 0.83 μmol) were added. NMR was measured at r.t., the decomposition of **Zn-1** was observed, and signals of free **B** were found in the NMR spectrum. After heating at 80 °C for 2 h, the released free **B** was removed by precipitation, and the precipitate was washed with Et_2O three times to give **Zn-2** as an orange solid.

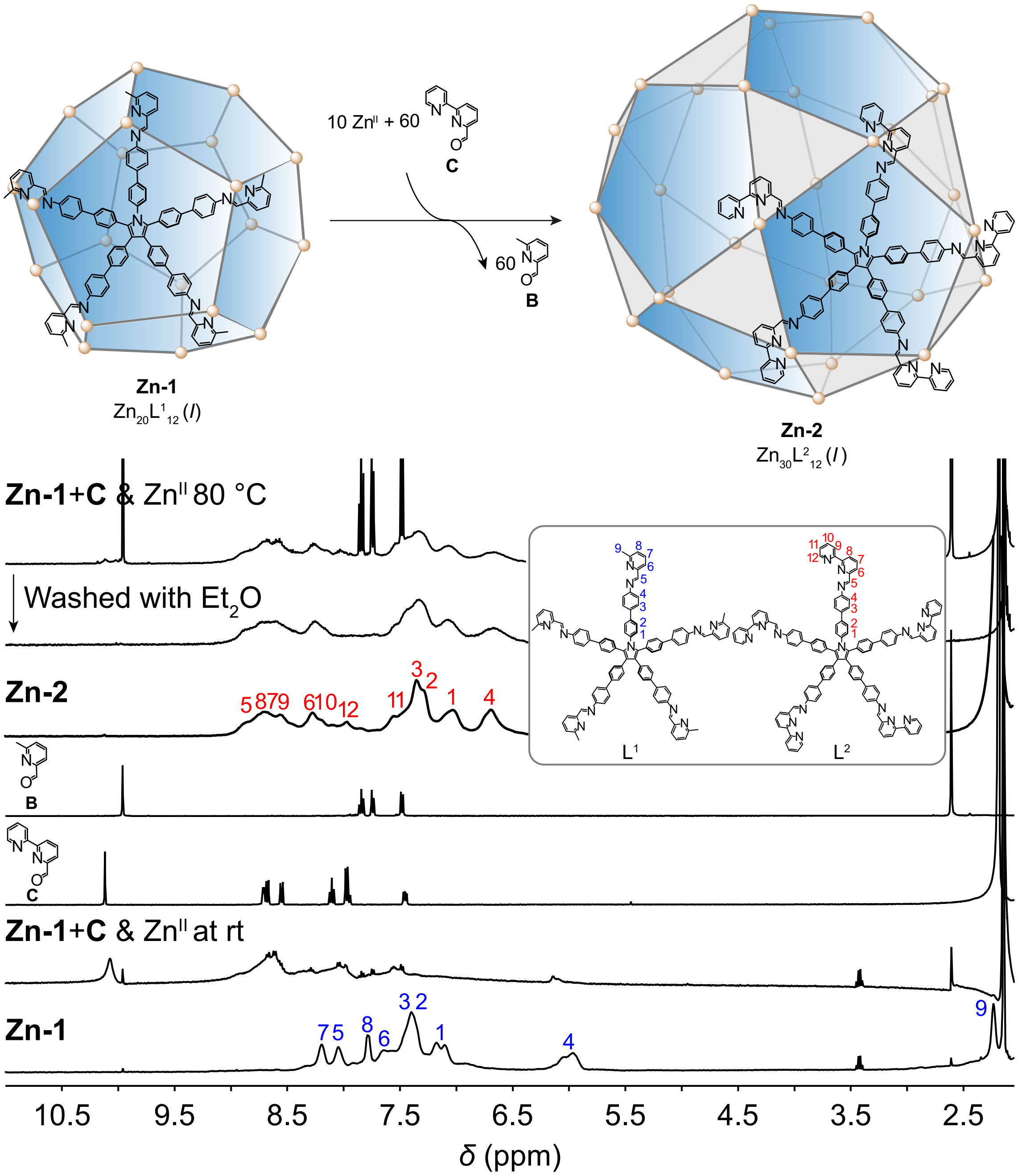

Figure S39. ^{1}H NMR spectra comparison (400 MHz, 298 K, CD_3CN) for the transformation from **Zn-1** **$[Zn_{20}L_{12}](NTf_2)_{40}$** to **Zn-2** **$[Zn_{30}L_{12}](NTf_2)_{60}$** cage after heating. The addition of aldehyde **C** and Zn^{II} led to the decomposition of **Zn-1**, free **B** could be found. After transformation, the free **B** was removed by precipitation and washing with Et_2O. (The broadness of **Zn-2** was observed in different batches of samples)

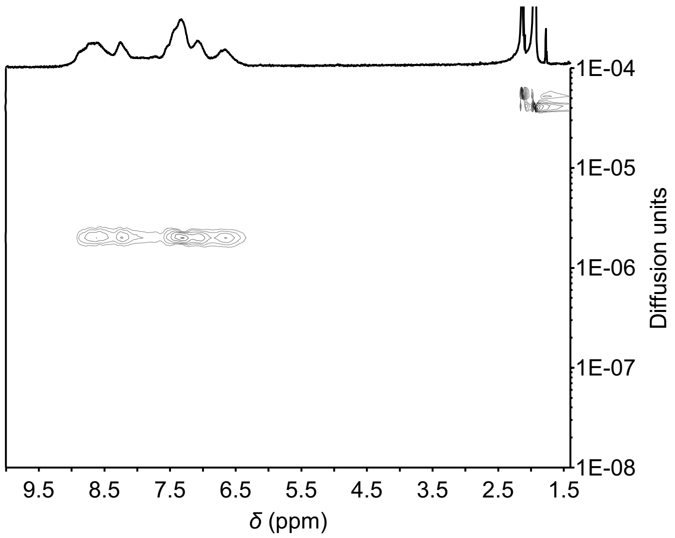

Figure S40. ^{1}H DOSY spectrum (400 MHz, 298 K, CD_3CN) of **Zn-2 [$Zn_{30}L_{12}$](NTf_2)$_{60}$** obtained from **Zn-1**. Diffusion coefficient: $D = 1.97 \times 10^{-10}$ m^2 s^{-1}, $r = 30.0$ Å. $r_{model} = 28.8$ Å.

4.2. Cage-to-cage transformation from **Zn-5** to **Zn-2**

Subcomponent **A** (0.90 mg, 1.0 μmol), $Zn(NTf_2)_2$ (1.6 mg, 2.5 μmol), [2,2'-bipyridine]-6-carbaldehyde **C** (0.91 mg, 5.0 μmol) and CD_3CN (0.4 mL) were added to an NMR tube, and the mixture was sonicated for 1 min and heated at 35 °C for 0.5 h to give **Zn-5**. The solution was further heated at 80°C for 3 h to give icosidodecahedron **Zn-2**.

^{1}H NMR (500 MHz, CD_3CN) δ 8.8 (br), 8.7 (br), 8.6 (br), 8.5 (br), 8.3 (br), 8.2 (br), 7.8 (br), 7.5 (br), 7.3 (br), 7.3 (br), 7.0 (br), 6.7 (br).

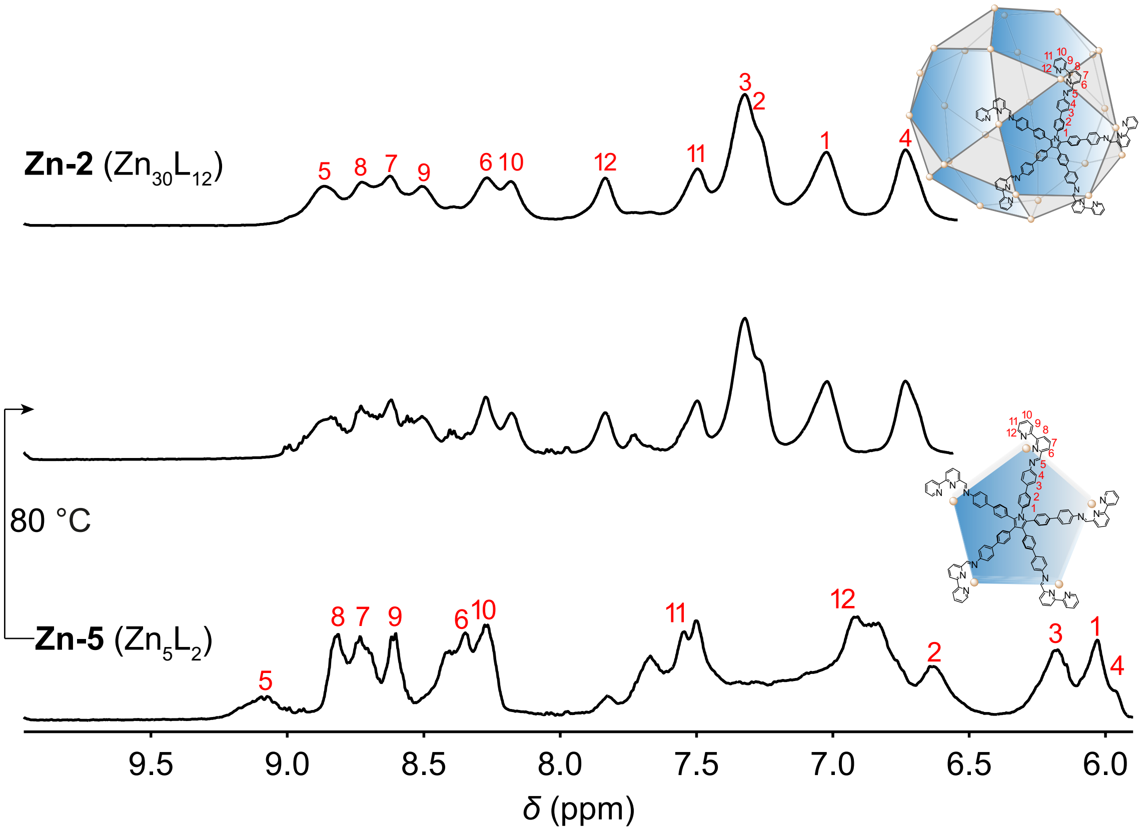

Figure S41. ^{1}H NMR spectra comparison (400 MHz, 298 K, CD_3CN) for the transformation from sandwich structure **Zn-5 $[Zn_5L_2](NTf_2)_{10}$** to **Zn-2 $[Zn_{30}L_{12}](NTf_2)_{60}$** cage after heating at 80 °C for 3 h.

4.3. Cage-to-cage transformation from **Zn-2** to **Zn-3**

To a **Zn-2 $[Zn_{30}L_{12}](NTf_2)_{60}$** (0.06 µmol) cage solution in CD_3CN (0.24 mL) was added **G** (15 eq., 112 µL, 8 mM, 0.9 µmol), the solution was kept at r.t. in an NMR tube for 40 d to give **Zn-3**. Heating was also tried to accelerate this process, but only partial conversion was observed. Heating overnight did not result in complete conversion.

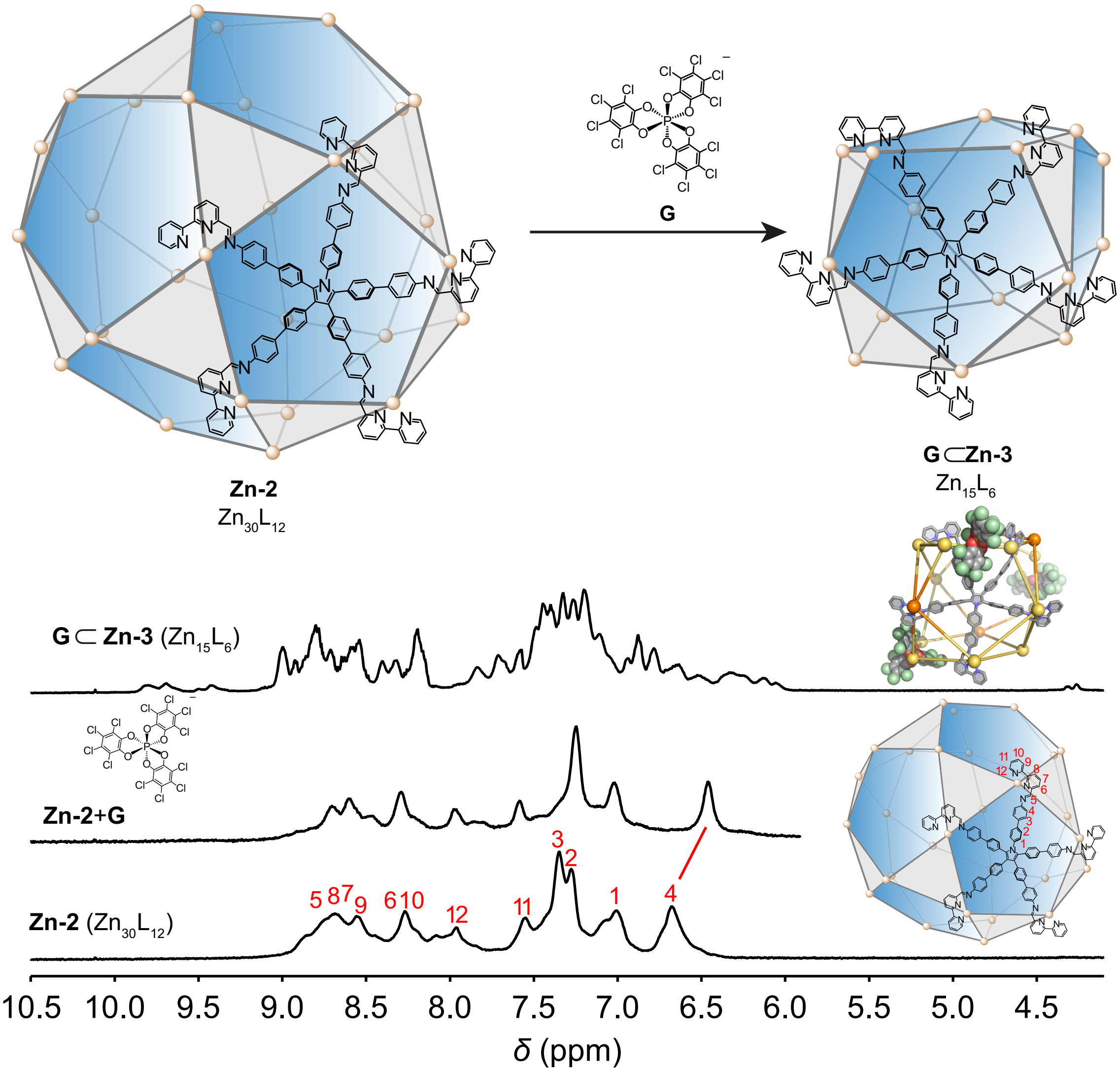

Figure S42. ^{1}H NMR spectra comparison (400 MHz, 298 K, CD_3CN) of **Zn-2** (bottom), **Zn-2** with 15 equiv. **G** at r.t. for 10 min (middle), and G⊂**Zn-3** after standing at r.t. for 40 days (Top).

5. SAXS measurements

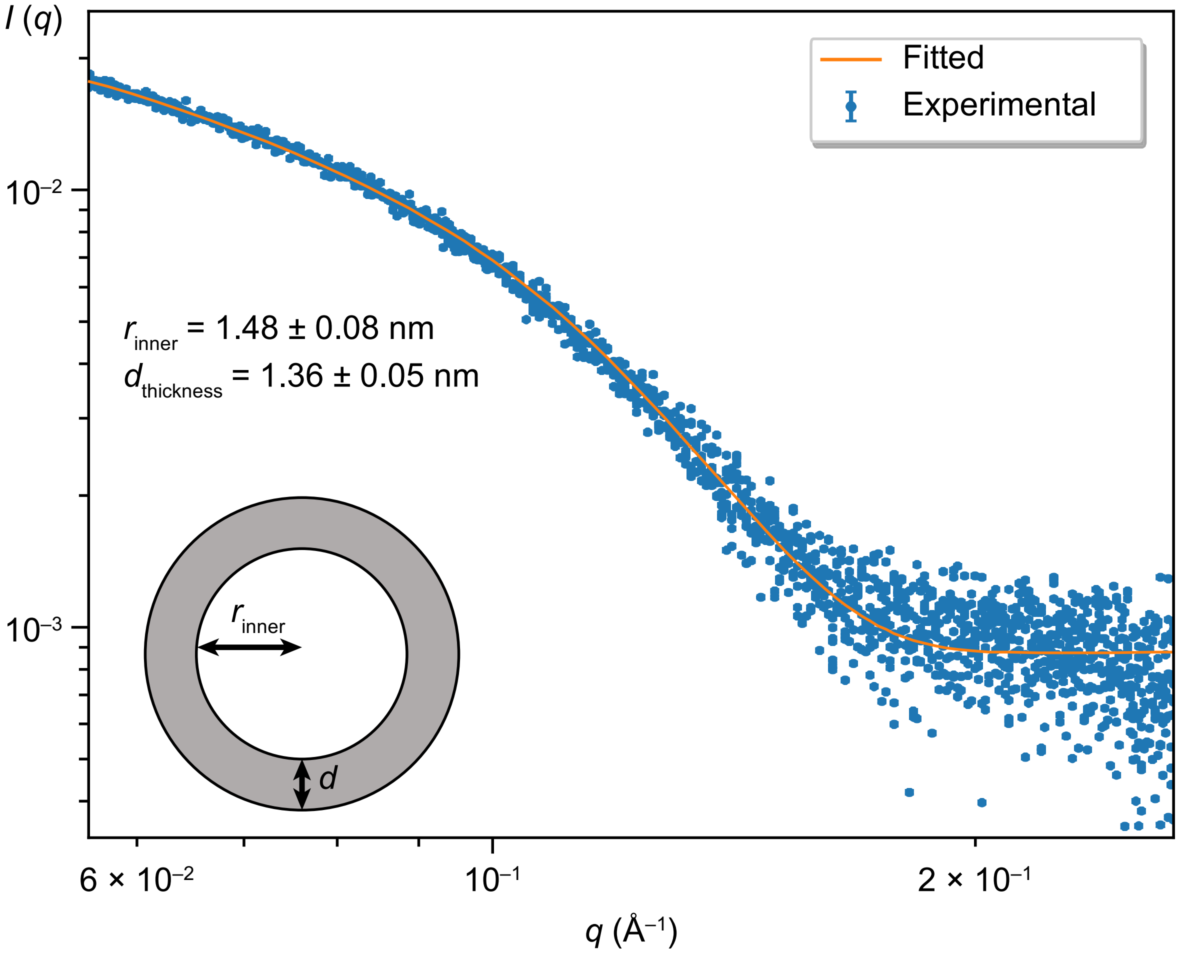

Figure S43. SAXS profile of dodecahedron **Zn-1 $[Zn_{20}L_{12}](NTf_2)_{40}$** in CD_3CN (0.55 mg mL^{-1}) and the fitted result based on a core-shell sphere model. The error bars represent the uncertainties in the scattering intensity.

6. X-ray Crystallography

All crystals diffracted to sub-atomic resolution, rendering direct methods unsuitable for structure solution. However, Δ-TRISPHAT (**G**) anions influenced the stereochemistries of the cages and forced them to crystallize in polar (enantiomorphic) space groups. That enabled the use of anomalous scattering for solving crystal structures. This is a common strategy for crystal structure solution of biomacromolecules and its effectiveness is not dependent on diffraction resolution.

Data for all 5 samples have been collected at different synchrotron beamlines (Table S1 and S2) that enable variable wavelength data collection and absorption edge scans necessary for single-wavelength anomalous dispersion/diffraction (SAD) and multiple-wavelength anomalous dispersion (MAD) experiments. Zinc K-edge fluorescence detected X-ray absorption spectra was recorded for **Zn-4** samples and cobalt K-edge absorption spectra was recorded for **Co-3** sample. Absorption spectra were processed with program CHOOCH[4] to identify appropriate wavelength(s) for data collection. Up to 3 wavelengths were selected for data collection. "**Peak**" wavelength is the maximum of the anomalous scattering factor f'' curve from CHOOCH. "**Inflection**" wavelength is the inflection point on the anomalous scattering factor f'' curve, which is also the minimum in anomalous scattering factor f' curve. High energy "**remote**" wavelength was selected to be around 100 eV higher than the peak wavelength. Each data collection scan was performed after re-centring the crystal to an unexposed part in order to minimise the effects of radiation damage from the previous scan (MAD experiment). If the crystal was too small, then only one scan was collected at the peak wavelength (SAD experiment). All 4 datasets of **Zn-4** diffract to around 1.7 Å.

Zn-4 (*I*422):

Crystals with composition $[Zn_{20}L_8]\cdot 12(C_{18}Cl_{12}O_6P)\cdot 12ClO_4\cdot 8$Toluene [+solvent] and *I*422 space group were grown by the diffusion of toluene into an acetonitrile solution of $[Zn_{30}L_{12}]\cdot 60(NTf_2)$ containing excess tetrabutylammonium perchlorate ($TBAClO_4$). MAD data were collected at beamline ID30B of ESRF employing synchrotron radiation (peak=1.28215 Å, inflection=1.28268 Å, remote=1.26514 Å) at 100(2) K and using EIGER2 X 6M 0.45 mm Si sensor detector. 3600 oscillation images were taken around the omega axis for each scan with a 0.2° step and 20 ms exposition time for a total of 720°. The X-ray beam was focused to 50 x 50 μm and attenuated to around 20%

transmission. Data integration and reduction were undertaken with the autoPROC[5], which relies on STARANISO[6], XDS[7], AIMLESS[8] and other programs from CCP4 suite[9] software packages.

Note that the same structure with *I*422 space group was also obtained from truncated rhombohedron Zn-3 sample, confirming truncated rhombohedron Zn-3 inevitably led to its transformation to truncated octahedron Zn-4.

SHELXC[10] was used to estimate substructure (zinc atoms) structure factor amplitudes (anomalous differences) from 3 MAD datasets. They were used by SHELXD[11,12] to identify the position of zinc atoms. SHELXE[13] was used to apply density modification to establish the correct handedness, improve zinc atom positions and produce phases for electron density map generation. Coot[14] was used to visualise and manipulate models and electron density. Molecular modelling software Maestro[15] was used to generate the initial model for the pentakis(tridentate) ligand which was then used to create SHELX and Refmac[16,17] dictionaries with GRADE program[18]. Refmac dictionary was imported to Coot to enable editing of ligand conformation.

The quality of the experimental electron density map produced by SHELXE[19] was sufficient to unambiguously place the ligand using Coot. Remote wavelength dataset was selected for refinement to avoid variation of dispersion coefficients near absorption edge. SHELX dictionary containing the full set of bond distance and angle restraints (DFIX, DANG, FLAT) was used for structure refinement with SHELXL[20]. Thermal parameter restraints (SIMU, RIGU) were applied to all atoms to facilitate a stable anisotropic refinement. Even with these restraints some thermal parameters remain larger than ideal as a consequence of the high level of thermal motion throughout the structure and especially around the vertex incorporating Zn^{II} and Δ-TRISPHAT.

The nitrogen atoms of the central pyrrole rings could not be resolved and each pentatopic ligand was assumed to be 5-fold rotationally disordered. Therefore, all atoms within the central pentagonal rings of the ligands were modelled as 80% carbon and 20% nitrogen. Carbon-bound hydrogen atoms were included in idealised positions and refined using a riding model. Disorder was modelled using standard crystallographic methods including constraints and restraints where necessary.

Rounds of restrained structure refinement with SHELXL were alternated with inspection of 2Fo-Fc and Fo-Fc electron density maps in Coot and fitting of additional anion (Δ-TRISPHAT and perchlorate) and solvent (toluene, water) molecules. Dictionaries for all

of them were created in the same way as for the ligand. The cage shows a high degree of crystallographic symmetry with 1/8 of the truncated octahedron (one crystallographically unique organic ligand, 1.5 Δ-TRISPHAT, and 2.5 Zn^{II} atoms) in the asymmetric unit. The absolute configuration of the crystal was determined by a density modification procedure in SHELXE and corroborated by the chirality of Δ-TRISPHAT used.

Additional anions per $Zn_{20}L_8$ assembly were required for charge balance. These anions (included as perchlorate in the formula above) were significantly disordered and despite numerous attempts at modelling, including with rigid bodies, no satisfactory model for the electron-density associated with them could be found. Therefore, the SQUEEZE[21] function of PLATON[22] was employed to remove the contribution of the electron density associated with the remaining anions and further highly disordered solvent, which gave a potential solvent accessible void of 60075 $Å^3$ per unit cell (a total of approximately 11083 electrons). Diffuse solvent molecules could not be assigned to acetonitrile or toluene and were therefore not included in the formula. Consequently, the molecular weight and density given above are underestimated.

CheckCIF of **Zn-4** (*I*422) gives 5 A and some B level alerts. These alerts result from the limited resolution of the data (low value of sine(theta_max)/wavelength, low data to parameter ratio and low bond precision) and the high level of thermal motion around the Zn^{II} vertex and Δ-TRISPHAT.

Zn-4 (*I*222):

Crystals with composition $[Zn_{20}L_8]\cdot 10(C_{18}Cl_{12}O_6P)\cdot 22ClO_4\cdot 4$Benzene [+solvent] and *I*222 space group were grown by the diffusion of benzene into an acetonitrile solution of $[Zn_{30}L_{12}]\cdot 60(NTf_2)$ containing excess tetrabutylammonium perrhenate ($TBAReO_4$). SAD data were collected at beamline I24 of Diamond Light Source employing synchrotron radiation (peak= 1.28160 Å) at 100(2) K and using Pilatus3 6M detector. 3600 oscillation images were taken around omega axis with 0.2° step and 20 ms exposition time for a total of 720°. X-ray beam was focused to 50 x 50 μm and attenuated to 4.9% transmission. Data integration and reduction were undertaken with the autoPROC, the same as for **Zn-4** (*I422*). This dataset diffracted to 1.55 Å.

Structure solution and restrained refinement was done with the same software and in the same way as for **Zn-4** (*I422*), except that SHELXC prepared data from only one dataset (SAD experiment). The quality of the experimental electron density map produced by

SHELXE with only peak dataset was sufficient to unambiguously place the ligand molecules using Coot.

Despite these measures and the use of synchrotron radiation few reflections at greater than 1.55 Å resolution were observed. Nevertheless, the quality of the data is sufficient to establish the connectivity of the structure. The cage shows a high degree of crystallographic symmetry with 1/4 of the truncated octahedron (two crystallographically unique organic ligands, 2.5 Δ-TRISPHAT, and 5 Zn^{II} atoms) in the asymmetric unit. The absolute configuration of the crystal was determined by a density modification procedure in SHELXE and corroborated by the chirality of Δ-TRISPHAT used.

The anions within the structure also show evidence of disorder and one of the located perrhenate anions were modelled as disordered over two locations. The disordered perrhenate anions were restrained to be approximately tetrahedral and most low occupancy oxygen atoms were modelled with isotropic thermal parameters.

Additional anions per $Zn_{20}L_8$ assembly are required for charge balance. These anions (included as perrhenate in the formula above) were significantly disordered and despite numerous attempts at modelling, including with rigid bodies no satisfactory model for the electron-density associated with them could be found. Therefore, the SQUEEZE[21] function of PLATON[22] was was employed to remove the contribution of the electron density associated with the remaining anions and further highly disordered solvent, which gave a potential solvent accessible void of 61414 $Å^3$ per unit cell (a total of approximately 14245 electrons). Diffuse solvent molecules could not be assigned to acetonitrile or benzene and were therefore not included in the formula. Consequently, the molecular weight and density given above are underestimated.

CheckCIF of **Zn-4** (*I*222) gives 3 A and some B level alerts. These alerts result from the limited resolution of the data (low value of sine(theta_max)/wavelength, low data to parameter ratio and low bond precision) and the high level of thermal motion around the Zn^{II} vertex and Δ-TRISPHAT.

Zn-4 (*F*222):

Crystals with composition $[Zn_{20}L_8]\cdot 12(C_{18}Cl_{12}O_6P)\cdot 14ReO_4$ [+solvent] and *F*222 space group were grown by the diffusion of THF to an acetonitrile solution of $[Zn_{30}L_{12}]\cdot 60(NTf_2)$ containing excess tetrabutylammonium perrhenate ($TBAReO_4$). SAD data were collected at beamline X06SA of Swiss Light Source employing synchrotron

radiation (peak=1.28256 Å) at 100(2) K and using EIGER 16M X detector. 3600 oscillation images were taken around omega axis with 0.2° step and 20 ms exposition time for a total of 720°. X-ray beam was focused to 50 x 50 μm and attenuated to 10% transmission. Data integration and reduction were undertaken with the autoPROC, the same as for **Zn-4** (*I422*). This dataset diffracted to 1.44 Å.
Structure solution and restrained refinement was done with the same software and in the same way as for **Zn-4** (*I422*), except that SHELXC prepared data from only one dataset (SAD experiment). The quality of the experimental electron density map produced by SHELXE with only peak dataset was sufficient to unambiguously place the ligand molecules using Coot.
The cage shows a high degree of crystallographic symmetry with 1/4 of the truncated octahedron (two crystallographically unique organic ligands, 3 Δ-TRISPHAT, and 5 Zn^{II} atoms) in the asymmetric unit. The absolute configuration of the crystal was determined by a density modification procedure in SHELXE and corroborated by the chirality of Δ-TRISPHAT used.
CheckCIF of **Zn-4** (*F*222) gives 7 A and some B level alerts. These alerts result from the limited resolution of the data (low value of sine(theta_max)/wavelength, low data to parameter ratio and low bond precision) and the high level of thermal motion around the Zn^{II} vertex and Δ-TRISPHAT.

Zn-4 ($P4_322$):
Crystals with composition $[Zn_{20}L_8]\cdot 8(C_{18}Cl_{12}O_6P)\cdot 7PF_6\cdot 2(CH_2Cl_2)$ [+solvent] and $P4_322$ space group were grown by the diffusion of diethyl ether/DCM to an acetonitrile solution of $[Zn_{30}L_{12}]\cdot 60(NTf_2)$ containing excess tetrabutylammonium hexafluorophosphate ($TBAPF_6$). SAD data were collected at beamline X06SA of Swiss Light Source employing synchrotron radiation (peak=1.28256 Å) at 100(2) K and using EIGER 16M X detector. 3600 oscillation images were taken around omega axis with 0.2° step and 20 ms exposition time for a total of 720°. X-ray beam was focused to 50 x 50 μm and attenuated to 10% transmission. Data integration and reduction were undertaken with the autoPROC, the same as for **Zn-4** (*I*422). This dataset diffracted to 1.46 Å.
Structure solution and restrained refinement was done with the same software and in the same way as for **Zn-4** (*I*422), except that SHELXC prepared data from only one dataset (SAD experiment). The quality of the experimental electron density map produced by

SHELXE with only peak dataset was sufficient to unambiguously place the ligand molecules using Coot.

The cage shows a twofold crystallographic symmetry with 1/2 of the truncated octahedron (four crystallographically unique organic ligands, 4 Δ-TRISPHAT, and 10 Zn^{II} atoms) in the asymmetric unit. The absolute configuration of the crystal was determined by a density modification procedure in SHELXE and corroborated by the chirality of Δ-TRISPHAT used.

CheckCIF of **Zn-4** ($P4_322$) gives 8 A and some B level alerts. These alerts result from the limited resolution of the data (low value of sine(theta_max)/wavelength, low data to parameter ratio and low bond precision) and the high level of thermal motion around the Zn^{II} vertex and Δ-TRISPHAT.

Table S1. Crystal data and structure refinement of truncated octahedron Zn-4.

Compound	**Zn-4**	**Zn-4**	**Zn-4**	**Zn-4**
Identification code	I422_sq	I222_sq	F222_sq	*P*4$_3$22_sq
CCDC number	2239369	2239370	2239371	2239372
Empirical formula	$C_{1224}H_{704}Cl_{156}N_{128}O_{156}P_{12}Zn_{20}$	$C_{1156}H_{664}Cl_{120}N_{128}O_{184}P_{10}Re_{22}Zn_{20}$	$C_{1168}H_{640}Cl_{144}N_{128}O_{160.4}P_{12}Re_{15.6}Zn_{20}$	$C_{1098}H_{644}Cl_{100}F_{42.33}N_{128}O_{68}P_{15.05}Zn_{20}$
Formula weight	26908.35	29257.61	28721.01	22840.30
Temperature [K]	100(2)	100(2)	100(2)	100(2)
Crystal system	tetragonal	orthorhombic	orthorhombic	tetragonal
Space group	*I*422 (97)	*I*222 (23)	*F*222 (22)	*P*4$_3$22 (95)
a [Å]	49.381(10)	46.521(10)	47.97(5)	49.89(5)
b [Å]	49.381(10)	48.027(10)	66.08(5)	49.89(5)
c [Å]	46.974(10)	50.962(10)	70.49(5)	91.92(9)
α [°]	90	90	90	90
β [°]	90	90	90	90
γ [°]	90	90	90	90
Volume [Å^3]	114545(52)	113863(41)	223408(329)	228809(510)
Z	2	2	4	4
ρ_{calc} [g cm^{-3}]	0.780	0.853	0.854	0.663
μ [mm^{-1}]	2.122	3.262	3.110	1.844
F(000)	27248	28816	56901	46315
Crystal size [mm^3]	0.100×0.050×0.050	0.100×0.100×0.100	0.100×0.100×0.050	0.100×0.050×0.050
Crystal color	orange	orange	orange	orange
Crystal shape	block	block	block	block
Radiation	Synchrotron ESRF (λ=1.26514 Å)	Synchrotron DLS (λ=1.28256 Å)	Synchrotron SLS (λ=1.28256 Å)	Synchrotron SLS (λ=1.28256 Å)
2θ range [°]	2.08 to 43.37 (1.71 Å)	2.10 to 48.76 (1.55 Å)	2.16 to 52.97 (1.44 Å)	1.47 to 52.11 (1.46 Å)
Index ranges	$0 \leq h \leq 26$<br>$0 \leq k \leq 20$<br>$0 \leq l \leq 27$	$0 \leq h \leq 24$<br>$0 \leq k \leq 29$<br>$0 \leq l \leq 31$	$0 \leq h \leq 32$<br>$0 \leq k \leq 45$<br>$0 \leq l \leq 42$	$0 \leq h \leq 34$<br>$0 \leq k \leq 23$<br>$0 \leq l \leq 45$
Reflections collected	2999	6504	8289	14399
Independent reflections	2999<br>R_{int} = 0.0327	6504<br>R_{int} = 0.0417	8289<br>R_{int} = 0.0360	14399<br>R_{int} = 0.0796
Completeness	89.5 %	76.3 %	79.9 %	68.9 %
Data/Restraints/Parameters	2999/3176/2393	6504/5837/4509	8289/5327/4175	14399/9975/7564
Goodness-of-fit on F^2	1.289	0.936	0.973	1.069
Final *R* indexes [$I \geq 2\sigma(I)$]	R_1 = 0.2695<br>wR_2 = 0.5119	R_1 = 0.1217<br>wR_2 = 0.3211	R_1 = 0.2656<br>wR_2 = 0.5471	R_1 = 0.2639<br>wR_2 = 0.5458
Final *R* indexes [all data]	R_1 = 0.2831<br>wR_2 = 0.5931	R_1 = 0.1340<br>wR_2 = 0.3478	R_1 = 0.2863<br>wR_2 = 0.6165	R_1 = 0.2859<br>wR_2 = 0.5903
Largest peak/hole [eÅ^{-3}]	1.00/−0.52	0.58/−0.41	2.09/−0.88	1.34/–0.71

Intriguingly, replacement of toluene with benzene also yielded single crystals, which crystallized in chiral *I*222 space group with one fourth of ligand in the asymmetric unit, leading to a distorted pseudo-truncated octahedron with approximate D_2 symmetry (Figure S44C, G), with two rhombus windows instead of square windows on the top and bottom compared to **Zn-4** with approximate D_4 symmetry. Two other different truncated octahedral structures with *F*222 and $P4_322$ space groups were also obtained by using THF and diethyl ether/CH_2Cl_2 as anti-solvents, respectively. Comparison of four of the Zn^{II} metal frameworks were shown to demonstrate their different symmetries (Figure S44). *F*222 structure also has D_2 symmetry, but the C_2 axis at the equatorial position is different compared to the *I*222 structure, for *I*222 structure, it goes through the two opposite equatorial windows, in contrast, for *F*222 one, it goes through the two opposite Zn centres. For the structure of $P4_322$, all the four edges of top and bottom windows have different distances, there is only one C_2 axis going through two opposite equatorial windows, leading to approximate C_2 symmetry. The slightly different structures of **Zn-4** make it flexible to adapt to external environment, reflecting the dynamic nature of Zn^{II}-N coordination.

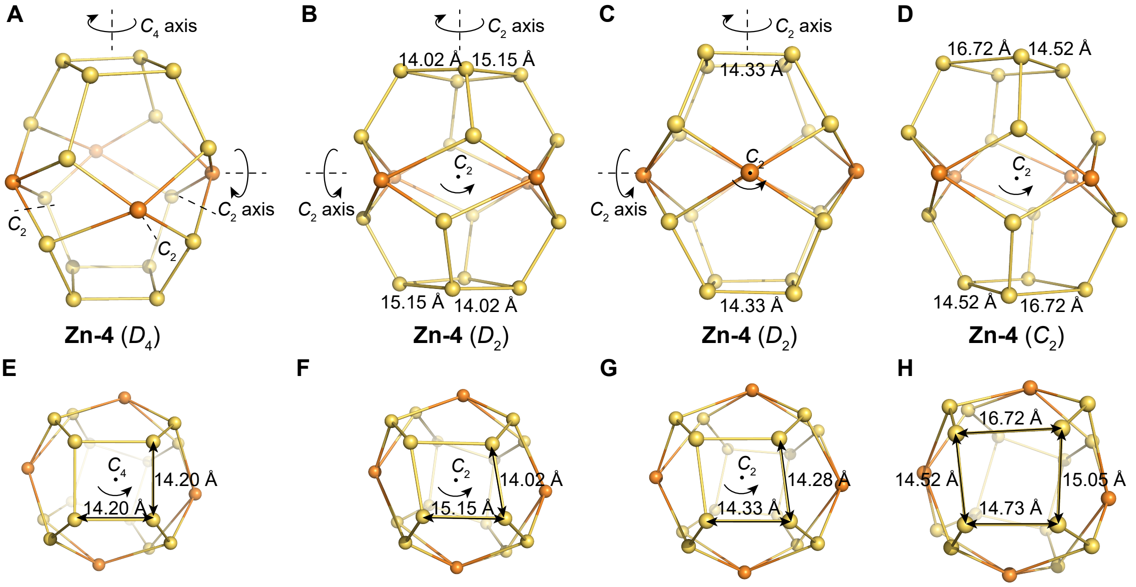

Figure S44. Comparison of four different Zn^{II} metal frameworks of crystal structures of Zn-4 with the axes and Zn^{II}–Zn^{II} distances shown. (A and E) **Zn-4** with *I*422 space group and D_4 symmetry grown by diffusing toluene to CH_3CN. (B and F) **Zn-4** with *I*222 space group and D_2 symmetry grown by diffusing benzene to CH_3CN. (C and G) **Zn-4** with *F*222 space group and D_2 symmetry grown by diffusing THF to CH_3CN.

(D and H) **Zn-4** with *P*$4_3$22 space group and C_2 symmetry grown by diffusing diethyl ether/CH_2Cl_2 to CH_3CN.

Co-3 (*P*$3_1$21): Crystals with composition $[Co_{15}L_6]\cdot10(C_{18}Cl_{12}O_6P)\cdot20NTf_2$ [+solvent] and *P*$3_1$21 space group were grown by diffusion of benzene into an acetonitrile solution of $[Co_{15}L_6]\cdot30(NTf_2)$. MAD data were collected at beamline I24 of Diamond Light Source employing synchrotron radiation (peak= 1.60310 Å, inflection=1.60560 Å, remote=1.58340 Å) at 100(2) K and using Pilatus3 6M detector. 1800 oscillation images were taken around omega axis with 0.2° step and 25 ms exposition time for a total of 360°. X-ray beam was focused to 50 x 50 µm and attenuated to 10% transmission for peak wavelength dataset and 34% transmission for inflection and high energy remote wavelength datasets. Data integration and reduction were undertaken with the autoPROC, the same as for **Zn-4** (*I422*). These datasets diffracted to between 2.69 Å (peak) and 2.79 Å (remote).
SHELXC was again used to analyse and prepare data for substructure (cobalt atoms) location. Although this crystal diffracts to significantly lower resolution than **Zn-4** cages and asymmetric unit is larger with more expected metal atoms, SHELXD identified all 15 cobalt atoms of the first cage molecule as well as several additional cobalt atoms that were later interpreted as belonging to the second copy of cage molecule. The quality of the experimental electron density map produced by SHELXE was sufficient to unambiguously place all ligand molecules of the first cage molecule (A) using Coot. Unfortunately, neither initial restrained refinement of the first cage molecule with SHELXL or SHELXE refinement of additional peaks from SHELXD provided reliable list of cobalt atoms for the second cage molecule.
Therefore, we employed phasing program PHASER[23] and density modification program PARROT[24] to use SAD data (only peak wavelength dataset), partial model (the cage A molecule after a round of refinement with SHELXL) and extensive list of peaks from SHELXD to generate refined list of cobalt atoms and new electron density map. That enabled us to fit second cage molecule near twofold crystallographic axis. Unfortunately, crystallographic axis doesn't correspond to internal cage symmetry, but duplicates all the atoms of the second cage molecule (B) making it disordered. It was possible to fit with Coot a copy of molecule A into the position for molecule B by overlaying metal atoms. However, that creates complex disorder and some close interatomic distances with cage molecule A. Additionally, refinement of molecule B improves R factor only marginally

and doesn't end up with reliable electron density for ligands of molecule B. For those reasons, we decided not to use cage molecule B ligands in final refinement. Metal atoms for molecule B came from phase refinement with Phaser and we kept them. PHASER/PARROT maps also enabled fitting of 10 Δ-TRISPHAT molecules attached to cage molecule A, thus confirming the absolute configuration of the cage determined by SHELXE. Weak electron density was identified for possible 11th Δ-TRISPHAT molecule, but it was close to two-fold crystallographic axis and obviously disordered. Therefore, we couldn't reliably fit it. Peak wavelength dataset was used for the refinement since it has slightly higher resolution than other two and it was also needed for Phaser runs. Additional DFIX commands were added in refinement to maintain ligand conformations without too short intramolecular contacts. We didn't treat diffuse solvent with SQUEEZE for this structure since cage molecule B has been omitted from the refinement.

Co-3 crystallized in the chiral $P3_121$ space group, with one and half whole cages in the asymmetric unit. Three out of eleven **G** anions bound at the pores where pentagonal faces share edges. The driving forces were inferred to be arene stacking between phenylene rings from different ligand arms and the tetrachlorocatecholate rings of **G**, as well as electrostatic interactions between anionic **G** and cationic Co^{II} centers. All Co^{II} centers were bound by two tridentate ligand arms, with three out of the fifteen Co^{II} centers adopting opposite Λ handedness, resulting in different $Co^{II}\cdots Co^{II}$ distances.

Table S2. Crystal data and structure refinement of truncated rhombohedron Co-3.

Compound	**Co-3**
Identification code	$Co_{15}L_6$
CCDC number	2240187
Empirical formula	$C_{1788}H_{960}Cl_{240}Co_{45}N_{192}O_{120}P_{20}$
Formula weight	38830.67
Temperature [K]	100(2)
Crystal system	trigonal
Space group (number)	$P3_121$ (152)
a [Å]	60.12(6)
b [Å]	60.12(6)
c [Å]	112.43(11)
α [°]	90
β [°]	90
γ [°]	120
Volume [$Å^3$]	351925(787)
Z	3
ρ_{calc} [gcm^{-3}]	0.550
μ [mm^{-1}]	3.057
F(000)	58761
Crystal size [mm^3]	0.100×0.100×0.050
Crystal color	orange
Crystal shape	block
Radiation	Synchrotron DLS (λ=1.6031 Å)
2Θ range [°]	11.50 to 34.68 (2.69 Å)
Index ranges	$0 \le h \le 18$<br>$0 \le k \le 10$<br>$-38 \le l \le 38$
Reflections collected	5690
Independent reflections	5690<br>R_{int} = 0.0391
Completeness to Θ = 17.338°	81.9 %
Data / Restraints / Parameters	5690/6342/2619
Goodness-of-fit on F^2	1.246
Final *R* indexes [$I \geq 2\sigma(I)$]	R_1 = 0.4249<br>wR_2 = 0.7041
Final *R* indexes [all data]	R_1 = 0.4321<br>wR_2 = 0.7324
Largest peak/hole [$eÅ^{-3}$]	0.38/–0.35

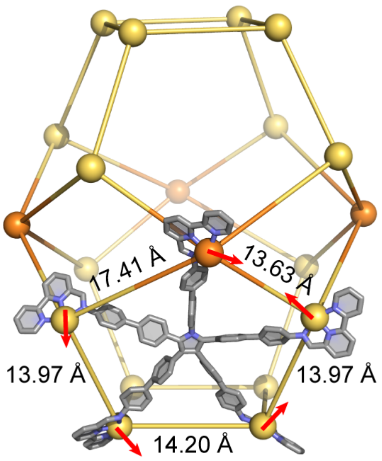

Figure S45. Schematic representation showing the shape of the truncated octahedron **Zn-4** with the different Zn^{II}–Zn^{II} distances shown on the metal framework. The directions of the coordination vectors of each tridentate motif were shown with red arrows.

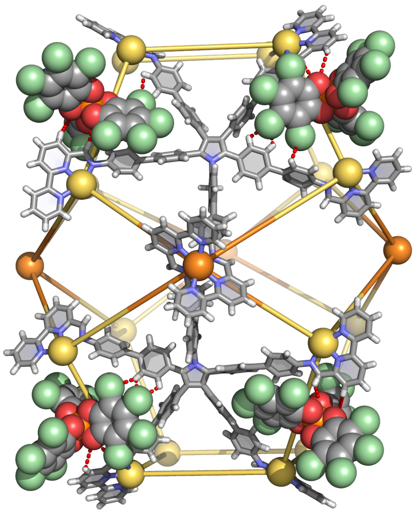

Figure S46. Schematic representation showing the multiple H···Cl and H···O interactions between host and guest, observed in the crystal structure of the truncated octahedron **Zn-4** (*I*422). Interactions are colored with red dash lines.

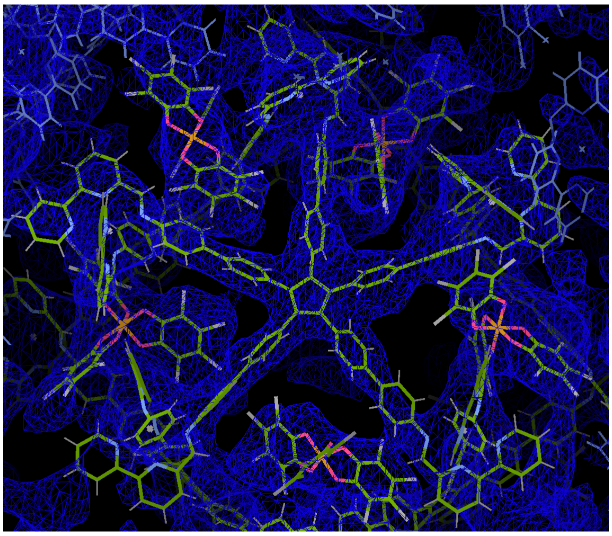

Figure S47. Enlarged view around one of the ligands in truncated rhombohedron **Co-3** with its Phaser/Parrot electron density map. The modelled ligand framework, metal ions and anionic guest **G** agree well with the observed electron density.

7. Optimized structures

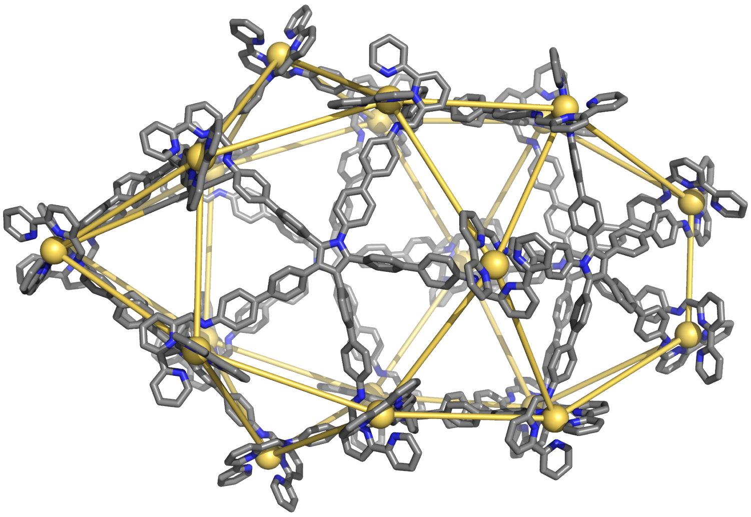

Figure S48. MM3 model of a $Zn^{II}_{20}L_8$ structure with pentagonal faces and 3-sided triangular windows as compared to the truncated octahedron **Zn-4** with the same $Zn^{II}_{20}L_8$ formula ($M_{5n}L_{2n}$ series, n = 4) but with pentagonal faces and 4-sided faces as windows shown in Figure S2.

7.1. Cartesian Coordinates of $Zn^{II}_{20}L_8$ isomer

Supplementary Table 3. Cartesian coordinates of $Zn^{II}_{20}L_8$ isomer with pentagons and 3-sided triangular windows (MM3)

C	45.903825	-27.089039	72.615068	H	15.28809	-29.79	64.64257
C	47.038278	-27.91501	72.721289	H	17.7011	-29.8962	65.26687
C	47.960104	-27.487617	71.754437	C	14.48388	-37.2031	64.26946
C	47.381763	-26.385102	71.097177	C	15.7774	-36.8062	64.61705
N	46.12665	-26.09643	71.650467	N	16.07745	-35.5081	64.7277
C	43.548133	-22.736245	70.678493	C	15.17713	-34.534	64.52022
C	44.733456	-23.03209	69.990618	C	13.86759	-34.8695	64.16955
C	45.548857	-24.132148	70.294405	C	13.51918	-36.2175	64.04376
C	45.290482	-25.060768	71.316219	H	14.23365	-38.2538	64.17706
C	44.09717	-24.748723	71.992455	H	16.53976	-37.5493	64.79607
C	43.265717	-23.657102	71.695868	H	13.12645	-34.0997	63.99476
H	45.073867	-22.37791	69.197379	H	12.50741	-36.4967	63.77245
H	46.426329	-24.227685	69.677286	Zn	17.89996	-34.8083	65.2389
H	43.784185	-25.330671	72.843907	C	18.46788	-34.4573	62.25731
H	42.375719	-23.528488	72.300031	C	17.82922	-33.2158	62.1005
C	49.381531	-24.837197	67.705574	C	17.67805	-32.5113	60.89372
C	49.461875	-24.184108	68.945302	C	18.16969	-32.9787	59.66864
C	48.81078	-24.668571	70.091666	C	18.85214	-34.1922	59.79209
C	48.026468	-25.828525	70.049348	C	18.9844	-34.8736	61.01013
C	47.940305	-26.48086	68.815253	H	17.38076	-32.7302	62.93809
C	48.595547	-25.997536	67.678167	H	17.12403	-31.5799	60.91663
H	50.063838	-23.28741	69.034026	H	19.3019	-34.6393	58.91295
H	48.917159	-24.139768	71.031811	H	19.53713	-35.7928	60.89102
H	47.350826	-27.387639	68.734338	C	19.12974	-36.3221	63.37886
H	48.471635	-26.549762	66.753935	C	18.8953	-38.36	67.09302
C	51.632585	-29.540799	71.454435	C	19.52473	-39.042	66.03989
C	51.597878	-28.143062	71.545476	C	19.66301	-38.4595	64.77075
C	50.383623	-27.440862	71.599702	C	19.15966	-37.1725	64.58074
C	49.148275	-28.100933	71.554611	N	18.59617	-36.6079	65.62796
C	49.188527	-29.489343	71.383259	C	18.40707	-37.0738	66.85525

C	50.396663	-30.189726	71.340501	H	18.79432	-38.823	68.06653
H	52.527852	-27.595067	71.643133	H	19.90803	-40.0417	66.20884
H	50.405728	-26.367973	71.748697	H	20.1395	-38.9928	63.95718
H	48.264987	-30.054082	71.328133	C	16.43466	-34.1533	69.20973
H	50.355872	-31.269265	71.24762	C	16.76556	-33.9711	67.86481
C	47.890496	-31.268139	75.163049	N	17.38842	-34.9403	67.18659
C	48.681729	-30.110842	75.173598	C	17.72071	-36.1172	67.7407
C	48.375819	-28.994635	74.385272	C	17.41519	-36.3573	69.08209
C	47.257203	-28.96422	73.543542	C	16.76577	-35.365	69.82218
C	46.441675	-30.099239	73.548717	H	15.93079	-33.3712	69.76627
C	46.759166	-31.216186	74.331786	H	16.51931	-33.044	67.36971
H	49.569757	-30.062034	75.791797	H	17.67617	-37.2984	69.54979
H	49.041945	-28.139553	74.412436	H	16.52055	-35.5338	70.8646
H	45.5775	-30.13613	72.895139	N	18.58519	-35.1692	63.32618
H	46.109934	-32.077489	74.236145	H	19.58486	-36.8097	62.50919
C	42.394791	-28.129759	74.741848	C	40.80629	-42.4031	71.90541
C	43.588917	-27.836401	75.415386	C	41.37964	-43.346	71.0347
C	44.757507	-27.467532	74.731121	C	42.47939	-43.1206	70.18976
C	44.794529	-27.373596	73.333597	C	43.11512	-41.8784	70.1141
C	43.614183	-27.711351	72.659355	C	42.57406	-40.9083	70.95907
C	42.445006	-28.06311	73.343349	C	41.49011	-41.172	71.80543
H	43.61047	-27.878294	76.498214	N	39.81294	-42.5616	72.70966
H	45.656332	-27.254707	75.29836	H	41.01327	-44.3482	71.00418
H	43.601285	-27.70557	71.574838	H	42.84462	-43.9304	69.56767
H	41.563035	-28.303503	72.760749	H	43.01254	-39.9159	70.96733
C	48.703332	-39.523674	65.294171	H	41.19714	-40.2985	72.36847
C	49.277113	-39.136096	66.522475	C	39.45624	-41.7267	73.6062
C	48.483143	-39.688457	67.541785	H	39.99598	-40.7911	73.79622
C	47.41196	-40.350844	66.915102	C	36.21214	-42.897	76.07686
N	47.562762	-40.305887	65.523314	C	36.8068	-41.6585	76.3648
C	45.131871	-42.412988	62.618532	C	37.88294	-41.1703	75.60802
C	44.960103	-42.528704	64.003729	C	38.34961	-41.9519	74.5512
C	45.726876	-41.810665	64.932323	N	37.72547	-43.0935	74.35155

C	46.754725	-40.913083	64.594991	C	36.7136	-43.6442	75.00935
C	46.874764	-40.775993	63.200607	H	35.38024	-43.259	76.66776
C	46.115174	-41.484518	62.255961	H	36.42813	-41.0669	77.1904
H	44.239083	-43.236271	64.395921	H	38.34484	-40.2178	75.83769
H	45.520866	-42.052784	65.962012	C	35.68451	-47.3471	73.22027
H	47.579878	-40.077871	62.782279	C	36.72799	-46.5333	72.77285
H	46.306188	-41.299323	61.205251	N	36.99424	-45.3792	73.39258
C	44.163188	-41.587734	69.310098	C	36.29782	-44.9444	74.45487
C	45.354669	-42.32751	69.289956	C	35.24275	-45.7158	74.94701
C	46.450326	-41.951994	68.495818	C	34.93522	-46.9285	74.32317
C	46.391414	-40.831737	67.657245	H	35.46135	-48.2839	72.72253
C	45.196238	-40.102792	67.664044	H	37.32211	-46.834	71.92303
C	44.113371	-40.468002	68.470628	H	34.66391	-45.383	75.79946
H	45.449764	-43.192098	69.93662	H	34.11938	-47.5401	74.69132
H	47.367859	-42.526898	68.542737	Zn	38.48822	-44.1315	72.86256
H	45.113044	-39.212356	67.05058	C	36.52866	-42.466	71.21582
H	43.220498	-39.853638	68.444745	C	35.90914	-41.801	72.28819
C	49.608858	-38.977198	71.541001	C	34.91192	-40.8164	72.17517
C	50.460046	-39.596824	70.618164	C	34.44118	-40.3606	70.93923
C	50.044564	-39.879239	69.31393	C	35.0519	-40.9752	69.84515
C	48.763265	-39.546633	68.856826	C	36.02691	-41.9711	69.99128
C	47.884236	-38.998195	69.79947	H	36.14416	-42.0668	73.29606
C	48.299069	-38.722321	71.111162	H	34.48613	-40.3947	73.07901
H	51.483977	-39.825778	70.890879	H	34.74421	-40.6876	68.84533
H	50.764025	-40.313855	68.628857	H	36.38877	-42.3198	69.0356
H	46.887238	-38.706688	69.491284	C	37.79068	-44.0951	70.25771
H	47.607275	-38.225111	71.781213	C	40.56082	-47.2084	71.05388
C	52.696518	-36.816273	67.402488	C	40.11689	-47.0853	69.7279
C	52.729864	-37.795565	66.402495	C	39.20523	-46.0873	69.35127
C	51.597867	-38.563704	66.092949	C	38.74816	-45.2112	70.33568
C	50.373994	-38.375829	66.750264	N	39.22342	-45.3997	71.54866
C	50.341817	-37.359803	67.715757	C	40.07534	-46.3086	72.00482
C	51.473628	-36.608928	68.047049	H	41.26898	-47.9802	71.32798

H	53.656508	-37.976465	65.870102	H	40.4859	-47.7758	68.97827
H	51.686985	-39.33931	65.341941	H	38.85984	-45.9989	68.32831
H	49.420789	-37.142693	68.243518	C	40.77357	-45.6746	76.13778
H	51.393288	-35.843366	68.810925	C	39.90344	-44.8967	75.37011
C	50.3751	-38.120237	61.672768	N	39.73196	-45.1619	74.07119
C	49.800509	-37.2627	62.618658	C	40.36522	-46.165	73.44231
C	49.253206	-37.747308	63.81296	C	41.24772	-46.9766	74.15846
C	49.25684	-39.111131	64.132161	C	41.45184	-46.7282	75.51897
C	49.879717	-39.965578	63.213031	H	40.91931	-45.4653	77.19146
C	50.407255	-39.481822	62.006687	H	39.36726	-44.0769	75.82413
H	49.741638	-36.200145	62.412	H	41.77354	-47.7879	73.67089
H	48.798241	-37.043423	64.500938	H	42.134	-47.3474	76.09036
H	49.932185	-41.026797	63.42909	N	37.41327	-43.4022	71.26053
H	50.848724	-40.188018	61.312981	H	37.36921	-43.9662	69.25357
C	33.700587	-40.767004	50.816422	C	31.04098	-17.7635	61.36857
C	32.358113	-40.584441	50.417303	C	29.78021	-17.7699	60.73749
C	31.554045	-41.152157	51.42441	C	28.85155	-18.3111	61.64894
C	32.429842	-41.668863	52.403282	C	29.58742	-18.6183	62.81634
N	33.763808	-41.478297	52.021567	N	30.92606	-18.2265	62.68452
C	37.34598	-42.925526	54.046617	C	34.00022	-18.2613	65.77716
C	36.043085	-43.227529	54.464659	C	32.69384	-18.7324	65.96572
C	34.89459	-42.741856	53.816933	C	31.71912	-18.7381	64.95684
C	34.889858	-41.900665	52.688044	C	31.91166	-18.2781	63.64221
C	36.206234	-41.597283	52.301971	C	33.2279	-17.8061	63.47751
C	37.364429	-42.078368	52.929866	C	34.21955	-17.798	64.4733
H	35.897829	-43.877301	55.319304	H	32.387	-19.0836	66.94356
H	33.969492	-43.118883	54.220449	H	30.75927	-19.1036	65.27855
H	36.395436	-40.941088	51.47097	H	33.53793	-17.3685	62.54343
H	38.309856	-41.78212	52.492579	H	35.19335	-17.4019	64.21059
C	31.082809	-43.197295	56.120764	C	27.82789	-20.7523	66.01671
C	31.823544	-42.01317	56.001918	C	27.65826	-19.3649	65.89836
C	32.246391	-41.518509	54.762708	C	28.22451	-18.6306	64.84324
C	31.97219	-42.18934	53.564202	C	29.01618	-19.2483	63.8664

C	31.188974	-43.346065	53.668873	C	29.1957	-20.6319	63.99
C	30.759011	-43.83452	54.913379	C	28.61203	-21.3641	65.0305
H	32.08881	-41.450136	56.889205	H	27.0488	-18.8452	66.62861
H	32.818767	-40.597712	54.735893	H	28.04996	-17.5629	64.78048
H	30.927509	-43.886846	52.766685	H	29.79411	-21.1562	63.25302
H	30.185358	-44.75365	54.936902	H	28.78007	-22.4345	65.06709
C	27.284574	-41.258749	51.482528	C	24.6786	-18.792	60.87912
C	28.051324	-40.735432	52.537287	C	25.38876	-17.6294	60.552
C	29.453062	-40.702365	52.526087	C	26.75547	-17.4838	60.82143
C	30.2002	-41.18361	51.445159	C	27.52537	-18.4889	61.42216
C	29.44649	-41.693837	50.380099	C	26.8064	-19.6448	61.76319
C	28.043693	-41.732866	50.400868	C	25.4327	-19.7912	61.50917
H	27.564097	-40.318175	53.409503	H	24.87152	-16.8017	60.08017
H	29.971117	-40.275505	53.377132	H	27.21778	-16.5424	60.55701
H	29.964802	-42.086891	49.512813	H	27.32481	-20.4764	62.22177
H	27.545712	-42.168236	49.543302	H	24.94936	-20.7224	61.78159
C	30.861961	-38.733705	46.877187	C	28.97526	-16.5475	56.75477
C	30.375857	-38.42874	48.158234	C	29.23826	-17.8841	57.08986
C	30.877998	-39.024637	49.323753	C	29.49792	-18.2917	58.40672
C	31.908517	-39.972509	49.294914	C	29.52744	-17.3793	59.46739
C	32.412593	-40.263418	48.019849	C	29.2767	-16.0413	59.13589
C	31.909985	-39.665403	46.854236	C	28.99763	-15.6389	57.82157
H	29.578372	-37.70463	48.274226	H	29.2354	-18.6377	56.31074
H	30.445572	-38.741363	50.276498	H	29.6868	-19.3397	58.60995
H	33.196474	-41.003909	47.918156	H	29.29193	-15.293	59.92014
H	32.332263	-39.971172	45.904353	H	28.81801	-14.5877	57.62768
C	36.872021	-38.957874	48.661782	C	34.63744	-16.8605	59.3122
C	36.632932	-40.337737	48.570834	C	34.29662	-18.1523	59.73587
C	35.578817	-40.964373	49.256301	C	33.09455	-18.4266	60.3992
C	34.715096	-40.243019	50.09247	C	32.16651	-17.422	60.70138
C	34.941714	-38.864798	50.174423	C	32.48126	-16.1354	60.245
C	35.987241	-38.243563	49.481901	C	33.68215	-15.8655	59.56851
H	37.274993	-40.94527	47.943896	H	34.97781	-18.9759	59.5549

H	35.437824	-42.03423	49.153565	H	32.88892	-19.4464	60.7057
H	34.300311	-38.259714	50.8057	H	31.7914	-15.3235	60.44101
H	36.113863	-37.17529	49.61375	H	33.88901	-14.8449	59.26799
C	48.870964	-34.923211	77.232021	C	36.87658	-18.1534	69.05908
C	49.822978	-33.989834	76.800426	C	36.87646	-17.2812	67.95712
C	49.522052	-32.787522	76.142174	C	35.96658	-17.3031	66.88445
C	48.209429	-32.38122	75.867534	C	34.92455	-18.234	66.77378
C	47.23691	-33.255714	76.360234	C	34.92507	-19.1483	67.83213
C	47.560714	-34.458138	76.998632	C	35.83923	-19.0977	68.89424
N	49.114218	-36.087103	77.71614	N	37.64635	-18.1478	70.09446
H	50.865112	-34.210682	76.893444	H	37.58914	-16.4881	67.89983
H	50.353381	-32.187881	75.790545	H	36.06682	-16.5349	66.12679
H	46.186344	-33.019689	76.237395	H	34.19001	-19.9444	67.84788
H	46.686558	-35.016376	77.300123	H	35.66791	-19.8937	69.603
C	48.240311	-37.000673	77.887307	C	37.50292	-18.9042	71.1125
H	47.172182	-36.852808	77.686779	H	36.70452	-19.6507	71.19421
C	49.793662	-40.874231	78.63146	C	40.10297	-18.1876	74.38958
C	48.402806	-40.775231	78.473218	C	39.13973	-19.1947	74.55857
C	47.782948	-39.538488	78.241171	C	38.23422	-19.5202	73.53715
C	48.592368	-38.40596	78.155214	C	38.31706	-18.8148	72.33664
N	49.891481	-38.597218	78.275617	N	39.25639	-17.8954	72.26796
C	50.560318	-39.711213	78.535743	C	40.14818	-17.5084	73.17062
H	50.253796	-41.829488	78.850675	H	40.7965	-17.9492	75.18613
H	47.793352	-41.668423	78.549752	H	39.09211	-19.7308	75.49943
H	46.708722	-39.460918	78.125752	H	37.48571	-20.291	73.67501
C	54.646857	-38.821241	79.204406	C	42.59435	-14.4508	71.54702
C	53.682319	-37.856537	78.903631	C	41.54462	-15.0557	70.85164
N	52.411193	-38.209508	78.680882	N	40.81502	-16.0127	71.43351
C	52.00052	-39.486544	78.747138	C	41.03748	-16.4376	72.68749
C	52.91783	-40.496618	79.04612	C	42.0737	-15.8698	73.43197
C	54.255358	-40.160505	79.272184	C	42.8581	-14.8667	72.85495
H	55.677639	-38.536605	79.382805	H	43.19146	-13.6748	71.08167
H	53.971486	-36.818803	78.851224	H	41.32014	-14.7528	69.83994

H	52.602605	-41.530308	79.112993	H	42.27436	-16.2	74.4435
H	54.981268	-40.930643	79.506986	H	43.66715	-14.4149	73.41766
Zn	50.926316	-36.918264	78.216401	Zn	39.26321	-16.9459	70.54441
C	51.153211	-37.437448	75.240666	C	41.17857	-19.1936	69.76323
C	51.77468	-37.316892	73.980844	C	41.21797	-19.8371	71.01091
C	51.275042	-37.84923	72.789328	C	41.96881	-20.983	71.32459
C	50.078843	-38.567028	72.740052	C	42.78026	-21.6508	70.39764
C	49.448304	-38.739222	73.976569	C	42.72697	-21.0624	69.12989
C	49.981623	-38.206816	75.162303	C	41.97827	-19.9112	68.84708
H	52.699867	-36.780586	73.830608	H	40.6794	-19.4367	71.84072
H	51.820581	-37.652714	71.872046	H	41.93309	-21.3363	72.3482
H	48.50884	-39.279174	74.015094	H	43.28336	-21.5044	68.31201
H	49.390041	-38.367071	76.039172	H	42.06828	-19.6196	67.81153
C	52.558849	-35.983955	76.272952	C	40.6763	-17.5113	68.31921
C	52.997256	-33.719882	79.827492	C	38.67248	-13.7884	67.95292
C	53.815038	-33.355557	78.746701	C	39.44168	-14.2001	66.85331
C	53.763848	-34.049805	77.529004	C	40.14027	-15.4171	66.86361
C	52.861135	-35.106956	77.417273	C	40.04807	-16.2175	68.00229
N	52.103227	-35.354217	78.467209	N	39.29786	-15.753	68.97922
C	52.109532	-34.785197	79.663758	C	38.61038	-14.6224	69.07091
H	53.077162	-33.201082	80.774437	H	38.13758	-12.8474	67.92973
H	54.512957	-32.53388	78.860066	H	39.49999	-13.563	65.97832
H	54.399127	-33.773815	76.695917	H	40.741	-15.7267	66.01712
C	49.545131	-36.907723	82.282365	C	36.6142	-14.456	72.8066
C	49.752028	-37.259158	80.94626	C	37.43686	-15.5141	72.41305
N	50.558869	-36.530453	80.166626	N	38.0352	-15.4935	71.21777
C	51.211718	-35.449337	80.624022	C	37.88919	-14.4814	70.34792
C	51.047384	-35.048905	81.952084	C	37.07859	-13.3955	70.68606
C	50.201626	-35.783073	82.787979	C	36.43635	-13.3841	71.92773
H	48.889849	-37.496263	82.91444	H	36.12494	-14.466	73.7739
H	49.253343	-38.127295	80.54513	H	37.59037	-16.3536	73.0742
H	51.573194	-34.185699	82.340354	H	36.94304	-12.5701	69.99843
H	50.063009	-35.488447	83.822001	H	35.80336	-12.5495	72.20726

N	51.610727	-36.838101	76.280218	N	40.55531	-18.1129	69.43799
H	53.12384	-35.716587	75.371914	H	41.32626	-17.8737	67.51415
C	55.866713	-34.17024	68.515713	C	20.50816	-19.1367	59.77744
C	55.39287	-35.201208	69.339516	C	21.01616	-19.4806	61.04172
C	54.369161	-36.093236	68.989025	C	22.36592	-19.3923	61.42809
C	53.753723	-36.047904	67.735432	C	23.36563	-18.9184	60.57026
C	54.257019	-35.095264	66.851448	C	22.89797	-18.5735	59.30083
C	55.260799	-34.200878	67.241041	C	21.55065	-18.6881	58.93792
N	56.687528	-33.248233	68.869026	N	19.28892	-19.1976	59.3684
H	55.751822	-35.295527	70.341328	H	20.36173	-19.8838	61.78659
H	54.015994	-36.811023	69.721563	H	22.64325	-19.6937	62.43204
H	53.820386	-35.012996	65.861708	H	23.60238	-18.2106	58.55987
H	55.523426	-33.506536	66.456542	H	21.37858	-18.3559	57.92516
C	56.909025	-32.183515	68.202449	C	18.90637	-19.0108	58.16529
H	56.459033	-31.986768	67.22171	H	19.60456	-18.8212	57.3413
C	58.93779	-29.119203	70.315395	C	14.74768	-19.3916	57.42563
C	58.47949	-28.797633	69.028623	C	15.56003	-19.1132	56.31545
C	57.811103	-29.742933	68.236704	C	16.94811	-18.9575	56.44747
C	57.599939	-31.013653	68.771413	C	17.50568	-19.1004	57.71803
N	58.029455	-31.216067	70.001691	N	16.66994	-19.387	58.69577
C	58.700518	-30.404594	70.806287	C	15.35219	-19.5248	58.67702
H	59.486203	-28.391268	70.899823	H	13.67485	-19.484	57.31243
H	58.660209	-27.805241	68.631765	H	15.10478	-19.0039	55.33777
H	57.462628	-29.496481	67.241121	H	17.57277	-18.7356	55.5908
C	59.916109	-32.494849	74.283766	C	13.91546	-20.1111	62.61934
C	59.187767	-33.077587	73.243832	C	15.26531	-19.9388	62.30351
N	58.816115	-32.352885	72.182407	N	15.64818	-19.7732	61.03285
C	59.130328	-31.053131	72.05689	C	14.77523	-19.7545	60.01268
C	59.859272	-30.414565	73.062838	C	13.41181	-19.921	60.26578
C	60.251797	-31.14209	74.189625	C	12.97913	-20.1039	61.58222
H	60.215979	-33.080393	75.14549	H	13.60077	-20.247	63.64781
H	58.925008	-34.121956	73.303095	H	16.00337	-19.9407	63.09083
H	60.129135	-29.369709	72.974423	H	12.69185	-19.9035	59.45717

H	60.819231	-30.66377	74.979818	H	11.92468	-20.2334	61.798
Zn	57.746316	-33.067255	70.622657	Zn	17.56551	-19.5289	60.44593
C	55.09018	-32.080928	71.696021	C	17.59954	-22.4835	59.75774
C	55.025681	-30.951726	70.863253	C	17.55139	-23.8499	60.10833
C	53.926713	-30.080445	70.770399	C	17.34136	-24.9069	59.21403
C	52.762236	-30.279924	71.518775	C	17.17896	-24.6964	57.84419
C	52.815454	-31.356986	72.404285	C	17.22504	-23.3544	57.45333
C	53.925737	-32.201356	72.482595	C	17.41256	-22.3137	58.37718
H	55.83995	-30.72559	70.20701	H	17.64642	-24.1871	61.1302
H	53.962257	-29.255167	70.068135	H	17.32972	-25.9228	59.5946
H	51.96641	-31.560439	73.049031	H	17.1244	-23.1121	56.40116
H	53.778056	-32.989066	73.205678	H	17.47825	-21.3376	57.94591
C	55.995313	-34.026513	72.444851	C	18.1835	-21.7626	61.83354
C	58.882436	-36.960308	71.443814	C	18.78206	-18.2905	64.19518
C	57.932802	-37.320515	72.412247	C	19.0679	-19.5222	64.80447
C	56.943551	-36.418852	72.8315	C	18.89858	-20.7335	64.11715
C	56.920862	-35.154593	72.243268	C	18.44809	-20.6814	62.7981
N	57.823985	-34.914245	71.313911	N	18.23203	-19.48	62.3003
C	58.815503	-35.681954	70.886125	C	18.33354	-18.2892	62.87305
H	59.665345	-37.652292	71.160233	H	18.89237	-17.3654	64.747
H	57.974854	-38.307535	72.858418	H	19.41332	-19.5391	65.83178
H	56.213276	-36.693153	73.583309	H	19.11004	-21.6836	64.59247
C	61.313503	-33.485336	68.265581	C	16.99354	-15.2884	60.19795
C	60.179525	-32.952861	68.883741	C	17.01737	-16.65	59.88693
N	59.419476	-33.708895	69.684397	N	17.46891	-17.5394	60.77753
C	59.711288	-34.994804	69.940486	C	17.9057	-17.1851	61.99669
C	60.833707	-35.583961	69.353792	C	17.9053	-15.838	62.36537
C	61.640285	-34.822365	68.503964	C	17.44729	-14.8811	61.45521
H	61.928423	-32.873458	67.615366	H	16.62995	-14.563	59.47909
H	59.920268	-31.920769	68.70865	H	16.67058	-16.9855	58.92158
H	61.088535	-36.616476	69.557073	H	18.24842	-15.5316	63.34559
H	62.516384	-35.261903	68.040834	H	17.43841	-13.8311	61.72448
N	56.023769	-32.962443	71.74037	N	17.80753	-21.5633	60.63112

H	55.200989	-34.257045	73.16425	H	18.41211	-22.7628	62.22276
C	16.84181	-29.095895	54.433915	C	27.35448	-26.4931	71.58273
C	16.493885	-28.915753	53.079698	C	26.34522	-27.4721	71.56451
C	16.565719	-30.176675	52.463938	C	26.53744	-28.8498	71.35899
C	17.012371	-31.08633	53.440008	C	27.79889	-29.4025	71.11615
N	17.137104	-30.444271	54.678755	C	28.83046	-28.4618	71.11406
C	17.992158	-32.351508	58.477958	C	28.60795	-27.0977	71.34164
C	17.83363	-33.051909	57.27511	N	27.24045	-25.2262	71.79158
C	17.596471	-32.420567	56.04574	H	25.32943	-27.2042	71.75627
C	17.474109	-31.031059	55.872722	H	25.67783	-29.5105	71.3815
C	17.683834	-30.34663	57.083174	H	29.84724	-28.7992	70.94267
C	17.925456	-30.961491	58.322237	H	29.52459	-26.5301	71.28697
H	17.844704	-34.13545	57.281362	C	28.21746	-24.4219	71.95651
H	17.436598	-33.09544	55.22073	H	29.26457	-24.7469	71.95208
H	17.681317	-29.269964	57.105237	C	27.21906	-20.3995	72.86634
H	18.063722	-30.324779	59.188344	C	28.58624	-20.7156	72.83033
C	18.239677	-34.911578	52.147378	C	29.02961	-22.0133	72.53257
C	16.876457	-34.609629	52.278014	C	28.06979	-22.9902	72.26813
C	16.433427	-33.352406	52.720213	N	26.81404	-22.5992	72.31717
C	17.338905	-32.347284	53.083473	C	26.29504	-21.4102	72.59444
C	18.699863	-32.65647	52.973899	H	26.89224	-19.3926	73.09371
C	19.138564	-33.902631	52.514488	H	29.31566	-19.9413	73.03865
H	16.142047	-35.350942	51.985064	H	30.08522	-22.2558	72.51444
H	15.370796	-33.143479	52.761335	C	22.06846	-21.5927	72.32268
H	19.438415	-31.900693	53.217786	C	22.90734	-22.6893	72.10978
H	20.205396	-34.074962	52.427422	N	24.23279	-22.5534	72.21739
C	15.620554	-30.414009	48.363746	C	24.82312	-21.389	72.5311
C	14.718773	-29.926552	49.317224	C	24.03539	-20.2578	72.75506
C	15.028867	-29.916336	50.679965	C	22.64528	-20.3626	72.65024
C	16.259589	-30.378702	51.162622	H	20.99259	-21.6941	72.23602
C	17.131255	-30.940274	50.22085	H	22.48687	-23.6508	71.85581
C	16.816146	-30.95805	48.85358	H	24.48988	-19.307	73.00415
H	13.773998	-29.497608	49.003131	H	22.01823	-19.4947	72.81997

H	14.312366	-29.478083	51.365823	Zn	25.5469	-24.0625	71.96066
H	18.103738	-31.295135	50.540699	C	25.96735	-23.1918	69.06685
H	17.556314	-31.329394	48.15429	C	26.78882	-22.0819	69.33103
C	15.390374	-25.455264	50.921442	C	27.39433	-21.2601	68.36468
C	16.332527	-26.365208	50.433341	C	27.25641	-21.4942	66.99337
C	16.713303	-27.485517	51.177937	C	26.46083	-22.5984	66.68452
C	16.15543	-27.776712	52.430719	C	25.85219	-23.3836	67.67214
C	15.226055	-26.8496	52.92259	H	26.96557	-21.7669	70.33602
C	14.859782	-25.70986	52.191806	H	27.99741	-20.419	68.68845
H	16.790035	-26.19555	49.464851	H	26.3006	-22.8518	65.6418
H	17.458547	-28.145449	50.750105	H	25.29022	-24.2005	67.24471
H	14.75514	-27.023598	53.882544	C	24.48558	-24.8302	69.60381
H	14.136731	-25.019646	52.610949	C	22.66183	-26.8791	72.84091
C	17.039499	-25.738638	56.994431	C	22.19467	-27.1925	71.55483
C	17.892061	-25.895021	55.895138	C	22.73114	-26.5798	70.41204
C	17.81096	-27.014366	55.057589	C	23.7512	-25.6446	70.58673
C	16.871051	-28.031825	55.266547	N	24.12806	-25.4204	71.82789
C	15.980268	-27.850706	56.332432	C	23.68432	-25.9367	72.9664
C	16.077016	-26.740625	57.18399	H	22.24069	-27.3632	73.71309
H	18.660545	-25.154647	55.70423	H	21.40144	-27.9225	71.44158
H	18.510537	-27.100508	54.23364	H	22.36296	-26.8183	69.42155
H	15.216288	-28.597712	56.516615	C	25.87352	-24.3291	76.22114
H	15.387066	-26.66373	58.016417	C	26.08812	-23.9686	74.88869
C	26.0786	-25.503933	43.561504	N	25.3535	-24.518	73.91635
C	24.683081	-25.32629	43.602735	C	24.38959	-25.422	74.15322
C	24.435649	-24.090202	44.217523	C	24.12975	-25.8214	75.46599
C	25.689441	-23.522461	44.51347	C	24.87909	-25.2681	76.50846
N	26.718439	-24.365674	44.07165	H	26.46558	-23.8889	77.01545
C	30.987003	-23.542071	44.143242	H	26.84997	-23.2452	74.64026
C	30.019754	-22.700469	44.711183	H	23.36032	-26.5525	75.68041
C	28.646319	-22.984343	44.705129	H	24.69136	-25.5665	77.5336
C	28.068904	-24.127596	44.128831	N	25.36561	-23.9584	69.90984
C	29.05314	-24.9585	43.563429	H	24.14123	-24.9924	68.57534

C	30.432133	-24.693946	43.570095	C	44.75451	-25.1135	52.7253
H	30.317269	-21.761786	45.161807	C	44.35124	-25.9831	51.6876
H	28.029987	-22.241132	45.182291	C	45.05499	-27.1876	51.87981
H	28.77172	-25.850866	43.028633	C	45.93315	-26.9866	52.96743
H	31.081169	-25.418802	43.093924	N	45.75601	-25.7058	53.5058
C	25.760636	-19.981269	46.828597	C	47.85722	-23.9391	56.88894
C	25.576502	-21.259185	47.374502	C	48.22413	-25.1585	56.30656
C	25.564515	-22.412866	46.583767	C	47.53964	-25.7201	55.21596
C	25.742427	-22.359383	45.197521	C	46.42195	-25.1535	54.57437
C	25.924066	-21.088081	44.638085	C	46.08512	-23.927	55.17197
C	25.94105	-19.933388	45.437459	C	46.75504	-23.3467	56.25862
H	25.4456	-21.381063	48.443373	H	49.06923	-25.704	56.70996
H	25.412101	-23.374187	47.062186	H	47.97431	-26.634	54.84756
H	26.050595	-20.998772	43.565326	H	45.22612	-23.372	54.83773
H	26.076536	-18.973616	44.952986	H	46.38518	-22.3908	56.60977
C	20.425037	-22.881204	44.712908	C	48.59947	-29.9466	54.42326
C	21.366682	-22.02228	44.130866	C	47.49293	-29.5047	55.16343
C	22.714966	-22.390613	44.001125	C	46.59075	-28.553	54.6686
C	23.187416	-23.628937	44.456147	C	46.75791	-27.9647	53.40812
C	22.259891	-24.449572	45.107926	C	47.82484	-28.4491	52.63977
C	20.915586	-24.088038	45.227464	C	48.71879	-29.4122	53.13276
H	21.035682	-21.079647	43.710604	H	47.33192	-29.8868	56.16486
H	23.390184	-21.727433	43.473771	H	45.76671	-28.2322	55.29619
H	22.569008	-25.414794	45.492329	H	47.98914	-28.0367	51.65083
H	20.241281	-24.788759	45.707043	H	49.55688	-29.7075	52.51215
C	21.461446	-27.819121	42.445733	C	44.42665	-30.8696	49.82401
C	21.648782	-26.537464	41.909211	C	44.36718	-30.7376	51.22003
C	22.734381	-25.731061	42.273031	C	44.59153	-29.5236	51.87984
C	23.70244	-26.15868	43.189967	C	44.88621	-28.3413	51.19193
C	23.545845	-27.447944	43.706269	C	44.93048	-28.4548	49.79616
C	22.45217	-28.24517	43.346004	C	44.71186	-29.6772	49.13879
H	20.935556	-26.132156	41.201971	H	44.11689	-31.5892	51.84055
H	22.809123	-24.736015	41.848951	H	44.5051	-29.5019	52.96011

H	24.25359	-27.816409	44.440189	H	45.14231	-27.5709	49.20572
H	22.370574	-29.207895	43.834897	H	44.77895	-29.6866	48.05765
C	27.639115	-29.269939	42.420131	C	41.69142	-25.1189	48.45335
C	27.849307	-28.713052	43.688475	C	41.55779	-26.2639	49.25725
C	27.329317	-27.463674	44.045455	C	42.41175	-26.5536	50.33333
C	26.594822	-26.681871	43.144858	C	43.46869	-25.7095	50.69566
C	26.356832	-27.247276	41.885237	C	43.59496	-24.5552	49.91094
C	26.865226	-28.507315	41.533994	C	42.74225	-24.2708	48.83403
H	28.416626	-29.262831	44.430718	H	40.76685	-26.975	49.05302
H	27.499735	-27.096411	45.051647	H	42.25183	-27.4662	50.89608
H	25.760285	-26.699279	41.16498	H	44.40735	-23.8672	50.11379
H	26.663998	-28.891049	40.540531	H	42.94366	-23.3735	48.26153
C	14.668572	-22.021868	48.47576	C	42.82393	-21.3849	53.29644
C	14.210343	-23.308604	48.158639	C	44.15658	-21.451	52.86343
C	14.416761	-24.445474	48.953345	C	44.81159	-22.673	52.64087
C	15.078463	-24.371528	50.181755	C	44.17372	-23.9005	52.86866
C	15.471781	-23.094665	50.578166	C	42.83654	-23.8364	53.27767
C	15.275941	-21.983761	49.74951	C	42.18165	-22.6163	53.48351
N	14.6305	-21.001304	47.697523	H	44.70211	-20.5311	52.68841
H	13.740285	-23.493978	47.217341	H	45.84358	-22.6702	52.30895
H	14.092117	-25.414372	48.589467	H	42.29444	-24.7573	53.46189
H	15.989396	-22.973184	51.523852	H	41.15278	-22.6407	53.82392
H	15.659198	-21.071775	50.182923	C	40.03278	-36.8194	46.60171
C	15.27364	-19.917529	47.894693	C	40.20136	-38.0533	47.25369
H	15.875871	-19.733719	48.792802	C	39.19401	-38.7773	47.91586
C	15.522637	-17.330314	44.545819	C	37.87493	-38.32	48.01133
C	16.258544	-16.96004	45.681858	C	37.66531	-37.0955	47.37395
C	16.233272	-17.735868	46.850171	C	38.68796	-36.4027	46.71423
C	15.464565	-18.899637	46.847275	N	40.91339	-36.1181	45.97421
N	14.837372	-19.190864	45.724406	H	41.16233	-38.5189	47.28539
C	14.769677	-18.505214	44.592371	H	39.45417	-39.7159	48.39238
H	15.521006	-16.701194	43.664673	H	36.67081	-36.6625	47.3767
H	16.844261	-16.048127	45.662755	H	38.32471	-35.4821	46.28421

H	16.796441	-17.446755	47.729143	C	40.68639	-35.0052	45.39224
C	11.983862	-20.318092	41.967032	H	39.70317	-34.5215	45.38454
C	12.280235	-20.807705	43.241381	C	43.96905	-33.1845	43.41832
N	13.188669	-20.19811	44.011496	C	42.74458	-32.5007	43.36211
C	13.841951	-19.094729	43.611985	C	41.59228	-33.0147	43.9758
C	13.587236	-18.55597	42.348702	C	41.69767	-34.228	44.65603
C	12.652021	-19.177027	41.516443	N	42.8893	-34.7887	44.6704
H	11.249496	-20.812307	41.341113	C	44.0242	-34.396	44.10991
H	11.768872	-21.68611	43.602255	H	44.84637	-32.7833	42.92668
H	14.097817	-17.661909	42.013618	H	42.68549	-31.5601	42.82675
H	12.440112	-18.772375	40.533331	H	40.647	-32.4879	43.92673
Zn	13.722157	-20.812508	45.862305	C	47.03989	-37.2913	44.79819
C	16.167881	-22.324706	44.906168	C	45.72763	-37.413	45.26152
C	17.088229	-21.329841	45.275002	N	44.83047	-36.4528	45.01346
C	18.486091	-21.459558	45.205682	C	45.13075	-35.3461	44.31507
C	19.096765	-22.63532	44.757664	C	46.42662	-35.168	43.82562
C	18.211595	-23.622806	44.323654	C	47.39016	-36.1503	44.07175
C	16.825542	-23.462889	44.395038	H	47.77103	-38.0665	44.99755
H	16.738359	-20.402926	45.679282	H	45.43757	-38.2871	45.82416
H	19.111777	-20.643393	45.549009	H	46.68801	-34.2836	43.25826
H	18.603257	-24.553811	43.926423	H	48.4009	-36.0299	43.69876
H	16.304058	-24.3367	44.034994	Zn	42.9072	-36.5107	45.6271
C	14.101504	-23.263062	44.786442	C	43.71886	-34.7415	47.97179
C	10.180051	-22.89604	46.345637	C	43.40192	-33.5058	47.38349
C	10.495263	-24.11338	45.722631	C	43.62393	-32.2411	47.95725
C	11.758945	-24.334346	45.155522	C	44.21048	-32.0651	49.21737
C	12.702227	-23.311012	45.244128	C	44.5611	-33.2774	49.81984
N	12.335704	-22.217061	45.881286	C	44.31952	-34.5249	49.23032
C	11.160739	-21.904008	46.407525	H	42.90888	-33.4774	46.43599
H	9.187298	-22.724484	46.742126	H	43.29829	-31.3682	47.40356
H	9.742872	-24.891355	45.661882	H	45.05697	-33.2726	50.78348
H	12.002233	-25.27125	44.668896	H	44.67827	-35.3361	49.8459
C	11.173004	-17.859246	47.661131	C	43.7297	-37.0107	48.14394

C	12.268387	-18.489672	47.065917	C	42.99018	-40.6192	46.0396
N	12.205859	-19.778861	46.713199	C	43.4221	-40.744	47.36928
C	11.099205	-20.51715	46.89913	C	43.69683	-39.6161	48.15737
C	9.970104	-19.942878	47.487724	C	43.5203	-38.3567	47.5842
C	10.009335	-18.601294	47.8768	N	43.09395	-38.3375	46.33811
H	11.223948	-16.815168	47.948538	C	42.82766	-39.3378	45.50981
H	13.170487	-17.924749	46.892218	H	42.79534	-41.499	45.43935
H	9.066903	-20.521227	47.63612	H	43.55478	-41.7324	47.79406
H	9.14221	-18.137848	48.3335	H	44.03936	-39.7153	49.18024
N	14.891171	-22.294654	45.047098	C	41.71083	-37.8019	41.7211
H	14.42495	-24.202361	44.323073	C	42.05527	-37.0002	42.81205
C	17.99783	-30.290693	41.739919	N	42.3892	-37.5574	43.98057
C	19.301876	-30.778309	41.961834	C	42.42018	-38.8866	44.168
C	20.435709	-29.98115	42.154763	C	42.08613	-39.7393	43.11358
C	20.38043	-28.584633	42.158578	C	41.72657	-39.1902	41.87939
C	19.107258	-28.071732	41.876571	H	41.43742	-37.3561	40.77148
C	17.987102	-28.891155	41.668238	H	42.05074	-35.9254	42.71269
N	16.972788	-31.061867	41.68599	H	42.10618	-40.8142	43.24283
H	19.524826	-31.834289	42.00544	H	41.46485	-39.8366	41.04947
H	21.380356	-30.484738	42.323599	N	43.52736	-35.9261	47.50291
H	18.950989	-26.999442	41.869952	H	44.05219	-37.0321	49.19172
H	17.063093	-28.372809	41.522969	C	51.62313	-32.4889	56.20413
C	16.980241	-32.303014	41.981147	C	51.05334	-32.713	54.93816
H	17.891337	-32.847196	42.258212	C	50.07096	-31.9112	54.32842
C	13.158577	-34.064447	42.494674	C	49.52022	-30.7867	54.95387
C	14.267105	-34.870765	42.795144	C	50.03983	-30.5471	56.22673
C	15.57717	-34.393061	42.643091	C	51.03002	-31.3539	56.80014
C	15.749003	-33.082363	42.198477	N	52.55137	-33.1587	56.79649
N	14.652222	-32.384042	41.979133	H	51.37484	-33.5409	54.34259
C	13.383394	-32.75886	42.053763	H	49.72356	-32.1728	53.33512
H	12.154232	-34.460153	42.577892	H	49.68719	-29.6867	56.78588
H	14.109127	-35.888251	43.133925	H	51.30154	-31.0008	57.78292
H	16.433047	-35.017579	42.868422	C	53.07627	-32.8476	57.91773

C	10.878947	-29.691001	40.552037	H	52.77856	-31.9662	58.49658
C	12.271425	-29.596054	40.610616	C	56.13925	-35.3981	59.36399
N	13.002081	-30.594811	41.119113	C	55.80524	-34.299	60.17035
C	12.445392	-31.72706	41.579497	C	54.81302	-33.3854	59.7842
C	11.057677	-31.882443	41.545582	C	54.15535	-33.6053	58.5739
C	10.266653	-30.851696	41.030942	N	54.52185	-34.6715	57.89277
H	10.285676	-28.881476	40.142193	C	55.45433	-35.5741	58.16023
H	12.756394	-28.706254	40.241333	H	56.91972	-36.0849	59.66656
H	10.592428	-32.792217	41.903543	H	56.32952	-34.1481	61.10701
H	9.188126	-30.955021	40.995049	H	54.5583	-32.536	60.40633
Zn	15.017345	-30.575279	41.280517	C	55.72865	-38.3434	54.96627
C	14.959365	-29.701097	44.180566	C	54.83756	-37.2677	54.98569
C	15.318461	-30.957296	44.694406	N	54.79906	-36.4375	56.03366
C	15.533204	-31.23729	46.054438	C	55.60214	-36.5852	57.09957
C	15.370491	-30.262519	47.044153	C	56.51473	-37.6417	57.13822
C	14.964013	-29.013227	46.572361	C	56.57639	-38.5303	56.0611
C	14.773334	-28.757792	45.21182	H	55.762	-39.0191	54.1192
H	15.514251	-31.769763	44.026384	H	54.17557	-37.1081	54.14859
H	15.857039	-32.230257	46.345527	H	57.1726	-37.7751	57.98781
H	14.832025	-28.193365	47.270971	H	57.27877	-39.356	56.07295
H	14.473895	-27.736736	45.027629	Zn	53.55501	-34.8527	56.18486
C	14.740387	-28.181471	42.508117	C	51.71046	-36.5839	57.87029
C	15.376855	-27.678282	38.349451	C	50.80917	-37.657	58.04399
C	15.186013	-26.508528	39.101033	C	50.39438	-38.1774	59.2771
C	14.949779	-26.561576	40.482618	C	50.83287	-37.6474	60.49149
C	14.925948	-27.815069	41.093459	C	51.72732	-36.5805	60.36544
N	15.155378	-28.857293	40.31951	C	52.1423	-36.0981	59.11423
C	15.344904	-28.908538	39.009156	H	50.38134	-38.1905	57.20787
H	15.517193	-27.623998	37.27727	H	49.68975	-39.0021	59.28949
H	15.20308	-25.546213	38.602323	H	52.10333	-36.1039	61.26404
H	14.795844	-25.659827	41.063094	H	52.79071	-35.2517	59.16188
C	15.411469	-32.942339	37.719628	C	51.47483	-36.4778	55.61209
C	15.231734	-32.556667	39.050208	C	53.02489	-34.6862	52.09289

N	15.244561	-31.263522	39.392863	C	51.93071	-35.5499	51.92946
C	15.414116	-30.284674	38.488803	C	51.33932	-36.1956	53.02587
C	15.596631	-30.607099	37.142029	C	51.86368	-35.9474	54.29438
C	15.600098	-31.949876	36.754784	N	52.87467	-35.1045	54.35346
H	15.403302	-33.990012	37.441067	C	53.51434	-34.4677	53.38206
H	15.081928	-33.313393	39.804012	H	53.4819	-34.2087	51.2353
H	15.725346	-29.830781	36.39829	H	51.54159	-35.7314	50.93418
H	15.738613	-32.219226	35.71387	H	50.5036	-36.8723	52.89537
N	14.865393	-29.375103	42.941949	C	56.73303	-32.2353	55.00089
H	14.642532	-27.307852	43.163708	C	55.86866	-33.0186	55.76944
C	41.026871	-44.488539	55.796036	N	54.86829	-33.6884	55.18808
C	39.767239	-45.104214	55.886873	C	54.64506	-33.6559	53.86463
C	38.568198	-44.614772	55.337892	C	55.47681	-32.8902	53.04463
C	38.476317	-43.409365	54.627806	C	56.52927	-32.1722	53.61986
C	39.706052	-42.745344	54.566389	H	57.54519	-31.6885	55.4662
C	40.890325	-43.26159	55.110814	H	56.00717	-33.0829	56.83795
N	42.166368	-44.914624	56.224	H	55.3162	-32.8512	51.9745
H	39.66617	-46.051233	56.370296	H	57.18543	-31.5732	52.99879
H	37.683065	-45.229388	55.449818	N	52.04057	-36.1467	56.70618
H	39.769621	-41.780104	54.07885	H	50.60726	-37.1486	55.57522
H	41.723881	-42.592872	54.957969	C	35.20985	-22.4589	44.08143
C	43.289462	-44.360578	55.978097	C	34.58943	-23.3269	43.16805
H	43.386799	-43.450402	55.374947	C	33.23237	-23.6944	43.17378
C	46.747983	-46.38617	57.372298	C	32.31284	-23.2425	44.13054
C	46.960974	-45.202895	56.64785	C	32.91414	-22.421	45.08975
C	45.888955	-44.450861	56.143477	C	34.26742	-22.0581	45.05325
C	44.594008	-44.90983	56.385087	N	36.42748	-22.0357	44.08702
N	44.492613	-46.023981	57.079295	H	35.14671	-23.7422	42.35728
C	45.434391	-46.80698	57.588092	H	32.88912	-24.336	42.3712
H	47.585324	-46.956206	57.754689	H	32.32311	-22.0345	45.91122
H	47.975375	-44.864017	56.47137	H	34.53116	-21.419	45.8823
H	46.057319	-43.542181	55.578467	C	36.88463	-21.1455	44.87884
C	43.644711	-50.083121	59.595184	H	36.27573	-20.6633	45.65228

C	42.919666	-49.085401	58.939201	C	40.89024	-19.9076	44.22825
N	43.556281	-48.082115	58.326588	C	40.19488	-19.2677	45.26615
C	44.894487	-47.976874	58.302502	C	38.86524	-19.599	45.56813
C	45.672643	-48.944651	58.94148	C	38.248	-20.5932	44.80882
C	45.040165	-50.007406	59.593235	N	38.98133	-21.1435	43.86341
H	43.135248	-50.899207	60.094743	C	40.23001	-20.8946	43.4941
H	41.840751	-49.121535	58.926782	H	41.9141	-19.6371	44.00292
H	46.75344	-48.877797	58.937224	H	40.69374	-18.4982	45.84391
H	45.627998	-50.76795	60.094521	H	38.3292	-19.0992	46.36566
Zn	42.625623	-46.593553	57.331882	C	41.28484	-23.3861	40.23231
C	42.859829	-44.890822	59.857036	C	40.03052	-23.4022	40.84698
C	44.177581	-44.450536	59.649149	N	39.77189	-22.5875	41.87519
C	44.923949	-43.637939	60.519362	C	40.67744	-21.7243	42.36215
C	44.419504	-43.153823	61.732345	C	41.94929	-21.6627	41.78848
C	43.095012	-43.539051	61.957321	C	42.25464	-22.5026	40.71359
C	42.37616	-44.352643	61.070324	H	41.50088	-24.0449	39.39891
H	44.71577	-44.749487	58.777883	H	39.2663	-24.0765	40.49154
H	45.947608	-43.407748	60.246694	H	42.69481	-20.9742	42.1663
H	42.590311	-43.201152	62.855307	H	43.23646	-22.4683	40.25523
H	41.375	-44.539936	61.427336	Zn	37.99932	-22.5076	42.83774
C	40.973957	-46.077654	59.398405	C	39.21131	-24.3633	44.94392
C	39.258368	-48.907942	56.743312	C	39.56956	-25.6326	45.45055
C	38.489402	-48.547092	57.860779	C	40.36058	-25.8805	46.58203
C	38.956553	-47.614765	58.799713	C	40.90051	-24.8652	47.3776
C	40.213137	-47.046269	58.591187	C	40.55255	-23.5894	46.91372
N	40.85593	-47.436789	57.51096	C	39.76091	-23.3704	45.77219
C	40.509962	-48.31221	56.57645	H	39.27407	-26.5502	44.96496
H	38.885191	-49.62964	56.027667	H	40.58086	-26.9143	46.82187
H	37.515795	-49.001454	58.004194	H	40.90039	-22.7195	47.4588
H	38.365329	-47.347008	59.666979	H	39.57757	-22.339	45.56584
C	43.572936	-48.582486	53.659247	C	37.99604	-25.1774	43.20041
C	43.643206	-47.756154	54.78336	C	36.01084	-24.1233	39.60088
N	42.635425	-47.725479	55.661285	C	36.06549	-25.4909	39.91223

C	41.526766	-48.469659	55.522029	C	36.69175	-25.9507	41.08081
C	41.399552	-49.315055	54.417708	C	37.2586	-25.0065	41.93695
C	42.433484	-49.371125	53.478486	N	37.14515	-23.7474	41.56993
H	44.385867	-48.611829	52.942673	C	36.5912	-23.2152	40.48851
H	44.513444	-47.138123	54.944787	H	35.52821	-23.7838	38.69307
H	40.512744	-49.921698	54.283674	H	35.62043	-26.2082	39.23227
H	42.35231	-50.021832	52.615136	H	36.74361	-27.0078	41.31135
N	42.150625	-45.672374	59.116081	C	37.00482	-18.998	40.59533
H	40.437145	-45.772405	60.303889	C	37.49685	-19.851	41.58615
C	22.931647	-41.395354	51.657202	N	37.32579	-21.1727	41.48298
C	23.805916	-41.454059	52.764177	C	36.69265	-21.7459	40.44699
C	25.20625	-41.418882	52.710749	C	36.17893	-20.9438	39.42563
C	25.926637	-41.302919	51.517395	C	36.33721	-19.557	39.5022
C	25.087937	-41.247854	50.395689	H	37.13851	-17.925	40.67272
C	23.685033	-41.297073	50.476179	H	38.01627	-19.4439	42.44055
N	21.650169	-41.432576	51.789716	H	35.66258	-21.3846	38.58205
H	23.445656	-41.564744	53.77592	H	35.94499	-18.9187	38.71855
H	25.739306	-41.505221	53.650126	N	38.50265	-24.2201	43.87536
H	25.529296	-41.141356	49.411806	H	38.07931	-26.2282	43.50127
H	23.189825	-41.212883	49.533732	C	38.66351	-29.0976	76.7
C	21.028787	-41.406321	52.903913	C	39.89396	-29.7083	76.99704
H	21.540936	-41.310981	53.86873	C	41.12502	-29.4078	76.38981
C	16.796947	-41.640864	52.718573	C	41.24372	-28.4532	75.37498
C	17.365643	-41.539114	53.997785	C	40.04114	-27.8308	75.03808
C	18.754887	-41.453455	54.175367	C	38.83361	-28.1413	75.67476
C	19.563437	-41.464411	53.038834	N	37.51489	-29.3199	77.23863
N	18.941023	-41.544032	51.881211	H	39.95571	-30.4642	77.74862
C	17.646684	-41.646931	51.610785	H	42.01264	-29.9444	76.70627
H	15.723618	-41.715591	52.597712	H	40.04087	-27.0696	74.26504
H	16.718913	-41.534126	54.867625	H	38.01629	-27.5599	75.27656
H	19.191705	-41.386304	55.164344	C	36.44569	-28.6738	76.97829
C	17.076173	-41.938973	47.420939	H	36.40391	-27.8479	76.25907
C	18.32988	-41.828127	48.027085	C	32.96377	-29.8356	79.10109

N	18.430337	-41.735704	49.35696	C	32.81931	-28.7787	78.18901
C	17.36121	-41.749568	50.169109	C	33.90737	-28.3087	77.43825
C	16.081414	-41.858793	49.620749	C	35.14366	-28.9295	77.61688
C	15.939194	-41.953423	48.233316	N	35.18123	-29.9228	78.48121
H	16.987414	-42.012462	46.343006	C	34.22183	-30.4233	79.24623
H	19.222663	-41.815232	47.420405	H	32.11835	-30.1775	79.68476
H	15.205439	-41.871536	50.257125	H	31.84916	-28.3108	78.06707
H	14.953627	-42.038985	47.790068	H	33.79491	-27.4902	76.73762
Zn	20.179636	-41.578787	50.351109	C	35.80079	-33.4849	81.71218
C	19.797775	-38.555399	50.427896	C	36.56306	-32.7573	80.79506
C	18.937433	-38.516524	51.539367	N	35.99454	-31.809	80.04254
C	18.397653	-37.354249	52.117441	C	34.68956	-31.5055	80.12898
C	18.703452	-36.076376	51.640504	C	33.8759	-32.1984	81.02811
C	19.570147	-36.066735	50.54755	C	34.43792	-33.1989	81.82607
C	20.073046	-37.24403	49.982161	H	36.2573	-34.2552	82.32319
H	18.61664	-39.42142	52.007588	H	37.61725	-32.9645	80.69341
H	17.731593	-37.444159	52.968479	H	32.82166	-31.9667	81.11361
H	19.856007	-35.114579	50.112606	H	33.82219	-33.7469	82.5301
H	20.732293	-37.034209	49.153822	Zn	36.98531	-30.6899	78.6836
C	20.972839	-39.556378	48.757305	C	35.99326	-32.5886	76.51311
C	22.304284	-43.288009	47.2469	C	36.01887	-33.8105	75.8051
C	22.639838	-42.150944	46.495536	C	35.06435	-34.2376	74.87249
C	22.245422	-40.866501	46.89941	C	33.95604	-33.4637	74.52252
C	21.512465	-40.747383	48.080203	C	33.89895	-32.236	75.19131
N	21.256124	-41.866315	48.726026	C	34.86365	-31.8432	76.13556
C	21.567059	-43.117806	48.42022	H	36.79195	-34.5492	75.95355
H	22.604818	-44.274188	46.916052	H	35.20232	-35.2028	74.39678
H	23.210295	-42.26742	45.581195	H	33.07348	-31.5657	74.97849
H	22.502663	-39.990263	46.31675	H	34.70125	-30.8722	76.54977
C	19.954763	-45.747212	51.325222	C	37.98107	-32.9317	77.5646
C	19.796127	-44.359792	51.361984	C	40.61152	-31.41	80.52429
N	20.340245	-43.591072	50.412541	C	41.01918	-32.5782	79.86146
C	21.04826	-44.097287	49.390244	C	40.22606	-33.1678	78.86538

C	21.240638	-45.477603	49.299405	C	39.016	-32.554	78.54179
C	20.689065	-46.309552	50.277863	N	38.72554	-31.4521	79.20213
H	19.516739	-46.374599	52.093141	C	39.3924	-30.8316	80.16581
H	19.232303	-43.906658	52.162902	H	41.2286	-30.9734	81.29945
H	21.807453	-45.905501	48.482077	H	41.96252	-33.0397	80.13007
H	20.827565	-47.383367	50.223608	H	40.53835	-34.0748	78.3622
N	20.29877	-39.586495	49.840499	C	37.1916	-27.4619	81.49358
H	21.183007	-38.632	48.207133	C	36.75301	-28.328	80.48909
C	29.094938	-37.016636	43.293316	N	37.50613	-29.3686	80.11805
C	28.40012	-37.379906	44.468215	C	38.69539	-29.6396	80.67931
C	28.967877	-37.930226	45.62519	C	39.18659	-28.8092	81.68912
C	30.335332	-38.187983	45.751995	C	38.42692	-27.7094	82.09816
C	31.060595	-37.845178	44.604189	H	36.58621	-26.6156	81.79765
C	30.462736	-37.29328	43.457546	H	35.8021	-28.1563	80.00815
N	28.495757	-36.516509	42.267224	H	40.14303	-29.0102	82.15531
H	27.331339	-37.26859	44.567569	H	38.79209	-27.054	82.88069
H	28.303135	-38.183024	46.4436	N	36.90868	-32.2706	77.36219
H	32.13376	-37.99867	44.598084	H	38.24279	-33.8306	76.99389
H	31.152652	-37.075998	42.672266	C	49.81591	-22.07	60.30158
C	27.244643	-36.275407	42.19547	C	50.45828	-22.8975	59.36483
H	26.550155	-36.463753	43.022038	C	49.84941	-23.5136	58.25647
C	25.822242	-34.682073	38.530647	C	48.48847	-23.3759	57.95168
C	24.866577	-34.895113	39.536362	C	47.81257	-22.5825	58.88325
C	25.226734	-35.41788	40.787673	C	48.44536	-21.9748	59.97563
C	26.570604	-35.717449	41.011165	N	50.32109	-21.4594	61.31841
N	27.396631	-35.471101	40.015988	H	51.50592	-23.0903	59.44846
C	27.155241	-34.997019	38.801247	H	50.47651	-24.1058	57.60036
H	25.528199	-34.284461	37.567481	H	46.7448	-22.4295	58.77934
H	23.827487	-34.656164	39.340998	H	47.74537	-21.4127	60.57451
H	24.486116	-35.588383	41.559572	C	49.68187	-20.6663	62.08736
C	30.7669	-34.802291	36.592963	H	48.62278	-20.4213	61.94866
C	30.685331	-35.270716	37.906545	C	51.88762	-18.8825	65.24067
N	29.509083	-35.299775	38.541665	C	50.53237	-18.5302	65.14422

C	28.365048	-34.895215	37.966954	C	49.70982	-19.0587	64.13784
C	28.383592	-34.416672	36.655035	C	50.27618	-19.9554	63.23177
C	29.597057	-34.369804	35.962744	N	51.55074	-20.2361	63.40725
H	31.717901	-34.775181	36.073222	C	52.40954	-19.7826	64.30975
H	31.576408	-35.609875	38.412555	H	52.5121	-18.4603	66.01791
H	27.472318	-34.084689	36.173557	H	50.1131	-17.8316	65.85916
H	29.630641	-34.001513	34.943678	H	48.6653	-18.7818	64.06285
Zn	29.257777	-35.960899	40.432451	C	56.23588	-21.4682	63.62237
C	29.414891	-33.088683	41.438281	C	55.11555	-21.7405	62.83354
C	28.071291	-32.754237	41.198016	N	53.93758	-21.1735	63.11414
C	27.468417	-31.519844	41.492299	C	53.76821	-20.3262	64.14186
C	28.169056	-30.472463	42.098023	C	54.85196	-20.015	64.96582
C	29.507652	-30.764798	42.363209	C	56.09717	-20.593	64.70287
C	30.082736	-32.000235	42.041428	H	57.19316	-21.9261	63.40067
H	27.412498	-33.45555	40.735925	H	55.20053	-22.4135	61.99401
H	26.419778	-31.377389	41.255494	H	54.73629	-19.3341	65.79979
H	30.130539	-30.006035	42.825018	H	56.94979	-20.3632	65.33176
H	31.121804	-32.041602	42.330418	Zn	52.24953	-21.4797	62.04985
C	31.26872	-34.40191	41.322577	C	51.38869	-23.5906	64.07279
C	32.69192	-38.248432	40.243636	C	50.74716	-22.7171	64.96766
C	33.647959	-37.333569	40.711761	C	50.10024	-23.0868	66.15919
C	33.288201	-36.032031	41.092847	C	50.01703	-24.4161	66.58608
C	31.944388	-35.669795	41.000045	C	50.65703	-25.3164	65.73163
N	31.117677	-36.597061	40.562566	C	51.29892	-24.9149	64.55386
C	31.359414	-37.838905	40.166705	H	50.7134	-21.6693	64.76922
H	32.986102	-39.246466	39.944214	H	49.62777	-22.316	66.75779
H	34.686706	-37.636707	40.775648	H	50.66955	-26.3706	65.98635
H	34.028363	-35.326336	41.450081	H	51.72899	-25.7557	64.03174
C	27.757195	-39.665522	38.884933	C	52.56282	-24.151	62.20454
C	27.836772	-38.352023	39.3537	C	54.39446	-22.6063	58.70491
N	29.009691	-37.843727	39.746324	C	54.49185	-23.9921	58.90535
C	30.152179	-38.547825	39.708707	C	53.92291	-24.6093	60.02969
C	30.135846	-39.866633	39.249296	C	53.247	-23.8052	60.94739

C	28.925728	-40.430003	38.834419	N	53.19382	-22.5193	60.66932
H	26.808631	-40.082723	38.566546	C	53.7082	-21.8432	59.65138
H	26.94569	-37.744928	39.400209	H	54.84642	-22.1426	57.8372
H	31.046304	-40.451451	39.210804	H	55.02293	-24.5976	58.17994
H	28.893672	-41.452062	38.474488	H	54.00579	-25.678	60.18592
N	30.018141	-34.195877	41.175313	C	52.8571	-17.7157	60.09361
H	31.964028	-33.633354	41.67951	C	52.45682	-18.6925	61.00857
C	30.348495	-36.6502	70.067584	N	52.75826	-19.9783	60.79949
C	30.293666	-35.390418	70.709564	C	53.45106	-20.3948	59.72755
C	29.146772	-34.747562	70.202261	C	53.87812	-19.4644	58.77759
C	28.497234	-35.649184	69.335422	C	53.57699	-18.112	58.96345
N	29.270308	-36.80701	69.187272	H	52.61494	-16.6719	60.25757
C	28.530255	-40.056019	66.382241	H	51.90018	-18.4077	61.88832
C	27.675711	-38.959657	66.557227	H	54.4376	-19.7794	57.90572
C	27.921616	-37.927893	67.472309	H	53.90119	-17.3753	58.23726
C	29.02116	-37.86346	68.344556	N	51.98627	-23.3059	62.96739
C	29.847098	-38.991093	68.178665	H	52.60499	-25.2243	62.42177
C	29.626566	-40.026852	67.25473	C	40.66023	-17.7038	54.03332
H	26.775225	-38.870884	65.962397	C	41.95627	-17.9771	54.50326
H	27.191378	-37.139148	67.439334	C	42.68663	-19.1537	54.26503
H	30.753364	-39.093954	68.751059	C	42.16953	-20.2218	53.52517
H	30.366568	-40.816462	67.202424	C	40.87906	-19.9956	53.04316
C	24.672143	-34.814266	67.67922	C	40.18386	-18.8034	53.28507
C	24.870593	-35.720344	68.730332	N	39.95585	-16.6387	54.2087
C	26.139153	-35.972054	69.271827	H	42.48864	-17.2518	55.07722
C	27.297772	-35.351887	68.78444	H	43.68276	-19.2453	54.68388
C	27.106389	-34.432033	67.746253	H	40.39661	-20.7672	52.45257
C	25.83255	-34.173814	67.215427	H	39.19961	-18.8211	52.84242
H	24.019569	-36.23743	69.158054	C	38.83328	-16.4051	53.64888
H	26.227277	-36.680476	70.08804	H	38.36094	-17.0997	52.94447
H	27.966264	-33.9173	67.334163	C	37.06934	-12.6316	54.45269
H	25.756967	-33.460096	66.403461	C	36.37614	-13.4745	53.56988
C	28.054979	-30.714825	70.901797	C	36.86638	-14.7463	53.23672

C	27.590009	-31.745057	71.73313	C	38.06828	-15.1577	53.81282
C	27.932941	-33.091128	71.521887	N	38.64194	-14.3045	54.63503
C	28.750285	-33.479948	70.453494	C	38.26857	-13.0866	55.00423
C	29.182437	-32.458162	69.599977	H	36.67854	-11.6527	54.70021
C	28.852914	-31.116109	69.822225	H	35.44374	-13.135	53.13366
H	26.973898	-31.49365	72.58865	H	36.33294	-15.3916	52.54925
H	27.570807	-33.848564	72.207298	C	41.09143	-11.4836	57.7293
H	29.811761	-32.707134	68.752606	C	41.20994	-12.746	57.14283
H	29.237881	-30.373487	69.132693	N	40.27791	-13.1832	56.28989
C	33.052724	-33.896856	73.610466	C	39.20251	-12.4541	55.95188
C	32.57111	-33.100177	72.563082	C	39.02979	-11.184	56.50652
C	31.668535	-33.587615	71.604379	C	39.98444	-10.6955	57.40323
C	31.168025	-34.898184	71.623671	H	41.84281	-11.123	58.42254
C	31.633575	-35.678095	72.691469	H	42.05629	-13.3729	57.37941
C	32.546283	-35.202841	73.642697	H	38.16807	-10.5804	56.25053
H	32.927792	-32.080914	72.468703	H	39.86682	-9.71193	57.84364
H	31.371466	-32.917191	70.809518	Zn	40.3437	-14.9748	55.36361
H	31.273054	-36.690928	72.810208	C	38.51901	-16.1167	57.52908
H	32.855714	-35.875565	74.43448	C	37.30327	-15.6292	57.01777
C	33.468172	-39.434226	70.769748	C	36.02981	-15.8443	57.57366
C	33.628332	-38.333674	69.915117	C	35.83259	-16.6152	58.7239
C	32.605471	-37.399431	69.695467	C	37.01523	-17.1306	59.25774
C	31.349729	-37.528901	70.300955	C	38.27106	-16.8846	58.68833
C	31.188997	-38.620242	71.166075	H	37.29216	-15.0117	56.14687
C	32.221278	-39.540616	71.400613	H	35.16534	-15.4026	57.09043
H	34.571721	-38.19545	69.3995	H	36.96697	-17.7316	60.15959
H	32.781829	-36.568671	69.021815	H	39.05825	-17.374	59.24126
H	30.234164	-38.763471	71.659332	C	40.78209	-16.3007	57.66756
H	32.031516	-40.377309	72.063041	C	44.4401	-15.1975	55.82397
C	30.078704	-44.718638	60.055336	C	44.53346	-15.8977	57.03692
C	29.188604	-44.675245	58.967955	C	43.38726	-16.3156	57.73018
C	29.482606	-44.196805	57.679298	C	42.14159	-16.0184	57.17732
C	30.735985	-43.677922	57.338793	N	42.15274	-15.3668	56.03363

C	31.652256	-43.702755	58.392324	C	43.17148	-14.9228	55.3097
C	31.331154	-44.197439	59.662676	H	45.33363	-14.8816	55.30041
N	29.866008	-45.154682	61.249509	H	45.51156	-16.1183	57.44875
H	28.192643	-45.045136	59.069425	H	43.46252	-16.8494	58.66984
H	28.706036	-44.220412	56.922849	C	41.72196	-12.9927	51.82579
H	32.660116	-43.339071	58.2231	C	40.89542	-13.5535	52.80244
H	32.168593	-44.12307	60.339398	N	41.42714	-14.1458	53.87655
C	30.751075	-45.249649	62.16398	C	42.75172	-14.2318	54.07814
H	31.800882	-44.973813	62.011878	C	43.62822	-13.6892	53.13596
C	29.483782	-46.893288	65.863899	C	43.10674	-13.0639	51.99967
C	30.838438	-46.539358	65.764559	H	41.29784	-12.5122	50.9515
C	31.364525	-45.983565	64.588385	H	39.82258	-13.5108	52.69037
C	30.499501	-45.785911	63.512129	H	44.70007	-13.7503	53.277
N	29.245335	-46.137593	63.701212	H	43.77196	-12.6375	51.25745
C	28.656399	-46.676409	64.760422	N	39.71244	-15.9282	57.07956
H	29.092399	-47.322453	66.777623	H	40.78476	-16.8213	58.63208
H	31.49316	-46.701819	66.613052	C	25.67711	-16.536	49.459
H	32.412222	-45.719031	64.512156	C	26.57982	-16.7066	48.39534
C	24.541916	-47.297093	63.954935	C	26.62453	-17.8036	47.51838
C	25.440492	-46.758622	63.030787	C	25.74596	-18.8863	47.62583
N	26.726151	-46.593078	63.357944	C	24.82066	-18.7501	48.66313
C	27.218698	-46.928635	64.560966	C	24.79843	-17.6398	49.51631
C	26.368025	-47.471255	65.526563	N	25.59835	-15.5565	50.29264
C	25.017103	-47.656971	65.218888	H	27.31818	-15.9653	48.1879
H	23.497977	-47.43351	63.69656	H	27.38174	-17.8206	46.74241
H	25.096613	-46.473514	62.048093	H	24.07989	-19.5272	48.81689
H	26.743509	-47.745859	66.504329	H	24.01624	-17.724	50.25498
H	24.342015	-48.077078	65.955771	C	24.72332	-15.4452	51.21488
Zn	28.124772	-45.847215	62.108833	H	23.93545	-16.1875	51.38633
C	27.7547	-43.086407	63.348712	C	24.91322	-11.9387	53.59397
C	28.524385	-43.293	64.505837	C	23.88336	-12.8448	53.89151
C	28.784847	-42.33482	65.501926	C	23.729	-14.0384	53.17011
C	28.308074	-41.017851	65.447865	C	24.63551	-14.3068	52.14459

C	27.571194	-40.771868	64.284286	N	25.57535	-13.4067	51.94663
C	27.310802	-41.747928	63.314421	C	25.79625	-12.2511	52.55866
H	28.934478	-44.257669	64.7132	H	25.01739	-11.0194	54.15646
H	29.368061	-42.649826	66.358878	H	23.18926	-12.6157	54.69193
H	27.183674	-39.779758	64.088521	H	22.92775	-14.7315	53.39588
H	26.740623	-41.35103	62.48784	C	29.1677	-10.3402	50.84263
C	26.614644	-43.705608	61.48096	C	28.66928	-11.5708	50.40847
C	25.9055	-46.911589	58.794548	N	27.60051	-12.1129	51.00118
C	25.170431	-45.735583	58.577841	C	26.95601	-11.5203	52.01902
C	25.326646	-44.612674	59.404707	C	27.40835	-10.2884	52.49684
C	26.241512	-44.693363	60.454575	C	28.52496	-9.69396	51.9019
N	26.890184	-45.832752	60.575359	H	30.03454	-9.89568	50.36703
C	26.808396	-46.945731	59.858914	H	29.14946	-12.0878	49.59156
H	25.773427	-47.770292	58.148416	H	26.90489	-9.79404	53.31814
H	24.465427	-45.69418	57.755556	H	28.88997	-8.73777	52.25915
H	24.754309	-43.708253	59.237829	Zn	26.77411	-13.8716	50.45549
C	29.49495	-49.841698	61.394837	C	28.35753	-15.28	52.65644
C	29.332407	-48.566992	61.942706	C	27.51606	-14.9388	53.72941
N	28.463294	-47.706863	61.402262	C	27.69533	-15.3312	55.06759
C	27.712026	-48.009011	60.331436	C	28.7495	-16.1547	55.47832
C	27.830969	-49.268608	59.740154	C	29.59396	-16.5415	54.43677
C	28.731311	-50.19269	60.278327	C	29.4005	-16.1157	53.11577
H	30.198621	-50.543936	61.827187	H	26.67564	-14.2975	53.57389
H	29.911246	-48.272747	62.805109	H	26.98837	-14.9841	55.81309
H	27.236783	-49.532373	58.874188	H	30.44376	-17.1776	54.66099
H	28.837095	-51.175142	59.832459	H	30.15013	-16.523	52.45441
N	27.451127	-43.925317	62.419307	C	29.15054	-15.1423	50.53067
H	26.049792	-42.770061	61.392157	C	28.37688	-13.588	46.66055
C	20.698443	-33.958263	66.137376	C	29.59389	-14.2654	46.83406
C	21.194359	-35.243663	66.414353	C	29.95202	-14.8192	48.07255
C	22.47455	-35.543882	66.911349	C	29.05872	-14.6819	49.13503
C	23.438419	-34.5636	67.175135	N	27.93918	-14.0385	48.87683
C	22.997302	-33.271657	66.881315	C	27.51598	-13.4745	47.75379

C	21.70788	-33.003663	66.398898	H	28.1157	-13.1631	45.69953
N	19.52073	-33.626153	65.72887	H	30.27289	-14.3609	45.99458
H	20.576156	-36.102621	66.274986	H	30.89342	-15.3385	48.20473
H	22.72479	-36.582634	67.096494	C	23.7146	-11.7081	48.37811
H	23.66738	-32.437299	67.058155	C	24.43525	-12.37	49.37517
H	21.557845	-31.946527	66.241455	N	25.63042	-12.904	49.10261
C	19.108891	-32.425711	65.594672	C	26.19592	-12.8343	47.88686
H	19.73739	-31.551757	65.800751	C	25.52307	-12.1844	46.84981
C	15.029964	-31.924908	64.537832	C	24.2704	-11.6165	47.09925
C	15.773801	-30.753322	64.748293	H	22.74451	-11.2741	48.59209
C	17.132093	-30.802173	65.097193	H	24.02529	-12.4526	50.37037
C	17.731879	-32.054939	65.226846	H	25.96059	-12.1183	45.86158
N	16.95814	-33.09612	65.001788	H	23.73344	-11.1086	46.30622
C	15.673047	-33.155993	64.679407	N	28.26558	-14.9189	51.42246
H	13.982144	-31.871421	64.270325	H	30.09473	-15.6574	50.7427

8. References

1. Wu, K., Ronson, T.K., Su, P., Chen, Z., Goh, L., Heard, A.W., Li, X., Klautzsch, F., Schalley, C.A., Vinković, M., and Nitschke, J.R. (2023). Systematic construction of progressively larger capsules from a fivefold linking pyrrole-based subcomponent. *Nat. Synth.*, DOI: 10.1038/s44160-023-00276-9..
2. Geometry optimized structures were modelled using the MM3 force field on SCIGRESS software (Fujitsu Limited, Tokyo, Japan, 2013) version FJ 2.6 (EU 3.1.9) Build 5996.8255.20141202.
3. Qu, H., Huang, Z., Dong, X., Wang, X., Tang, X., Li, Z., Gao, W., Liu, H., Huang, R., Zhao, Z., et al. (2020). Truncated face-rotating polyhedra constructed from pentagonal pentaphenylpyrrole through graph theory. J. Am. Chem. Soc. *142*, 16223–16228.
4. Evans, G., and Pettifer, R.F. (2001). CHOOCH: a program for deriving anomalous-scattering factors from X-ray fluorescence spectra. J. Appl. Cryst. *34*, 82–86.
5. Vonrhein, C., Flensburg, C., Keller, P., Sharff, A., Smart, O., Paciorek, W., Womack, T., and Bricogne, G. (2011). Data processing and analysis with the autoPROC toolbox. Acta Crystallogr. Sect. D *67*, 293–302.

6. Tickle, I.J., Flensburg, C., Keller, P., Paciorek, W., Sharff, A., Vonrhein, C., and Bricogne, G. (2018–2021). STARANISO. Cambridge, United Kingdom: Global Phasing Ltd.
7. Kabsch, W. (2010). XDS. Acta Crystallogr. Sect. D *66*, 125–132.
8. Evans, P.R., and Murshudov, G.N. (2013). How good are my data and what is the resolution? Acta Crystallogr. Sect. D *69*, 1204–1214.
9. Winn, M.D., Ballard, C.C., Cowtan, K.D., Dodson, E.J., Emsley, P., Evans, P.R., Keegan, R.M., Krissinel, E.B., Leslie, A.G.W., McCoy, A., et al. (2011). Overview of the CCP4 suite and current developments. Acta Crystallogr. Sect. D *67*, 235–242.
10. Sheldrick, G. (2008). A short history of SHELX. Acta Crystallogr. Sect. A *64*, 112–122.
11. Schneider, T.R., and Sheldrick, G.M. (2002). Substructure solution with SHELXD. Acta Crystallogr. Sect. D *58*, 1772–1779.
12. Usón, I., and Sheldrick, G.M. (1999). Advances in direct methods for protein crystallography. Curr. Opin. Struct. Biol. *9*, 643–648.
13. Sheldrick, G.M. (2002). Macromolecular phasing with SHELXE. Z. Kristallogr. *217*, 644–650.
14. Emsley, P., Lohkamp, B., Scott, W.G., and Cowtan, K. (2010). Features and development of Coot. Acta Crystallogr. Sect. D *66*, 486–501.
15. Schrödinger Release 2022: Maestro, Schrödinger, LLC, New York, NY, 2021.
16. Murshudov, G.N., Skubak, P., Lebedev, A.A., Pannu, N.S., Steiner, R.A., Nicholls, R.A., Winn, M.D., Long, F., and Vagin, A.A. (2011). REFMAC5 for the refinement of macromolecular crystal structures. Acta Crystallogr. Sect. D *67*, 355–367.
17. Murshudov, G.N., Vagin, A.A., and Dodson, E.J. (1997). Refinement of Macromolecular Structures by the Maximum-Likelihood Method. Acta Crystallogr. Sect. D *53*, 240–255.
18. Bricogne, G.B., E.; Brandle, M.; Flensburg, C.; Keller, P.; Paciorek, W.; Roversi, P.; Sharff, A.; Smart, O. S.; Vonrhein, C.; Womack, T. O (2011). BUSTER; 2.11.2 ed.; Global Phasing Ltd.: Cambridge, United Kingdom.
19. Hubschle, C.B., Sheldrick, G.M., and Dittrich, B. (2011). ShelXle: a Qt graphical user interface for SHELXL. J. Appl. Crystallogr. *44*, 1281–1284.

20. Sheldrick, G.M. (2015). Crystal structure refinement with SHELXL. Acta Crystallogr. Sect. C *71*, 3–8.
21. van der Sluis, P., and Spek, A.L. (1990). BYPASS: an effective method for the refinement of crystal structures containing disordered solvent regions. Acta Crystallogr. Sect. A *46*, 194–201.
22. Spek, A.L. (2015). PLATON SQUEEZE: a tool for the calculation of the disordered solvent contribution to the calculated structure factors. Acta Crystallogr. Sect. C *71*, 9–18.
23. McCoy, A.J., Grosse-Kunstleve, R.W., Adams, P.D., Winn, M.D., Storoni, L.C., and Read, R.J. (2007). Phaser crystallographic software. J. Appl. Cryst. *40*, 658–674.
24. Cowtan, K. (2010). Recent developments in classical density modification. Acta Crystallogr. Sect. D *66*, 470–478.
